# Cost-effectiveness of routine and campaign use of typhoid Vi-conjugate vaccine in Gavi-eligible countries: a modelling study

**DOI:** 10.1016/S1473-3099(18)30804-1

**Published:** 2019-07

**Authors:** Joke Bilcke, Marina Antillón, Zoë Pieters, Elise Kuylen, Linda Abboud, Kathleen M Neuzil, Andrew J Pollard, A David Paltiel, Virginia E Pitzer

**Affiliations:** a Centre for Health Economics Research and Modeling Infectious Diseases, Vaccine and Infectious Disease Institute, University of Antwerp, Antwerp, Belgium; b Department of Epidemiology of Microbial Diseases, Yale School of Public Health, Yale University, New Haven, CT, USA; c Department of Health Policy and Management, Yale School of Public Health, Yale University, New Haven, CT, USA; d Center for Statistics, I-Biostat, Hasselt University, Diepenbeek, Belgium; e Center for Vaccine Development and Global Health, University of Maryland School of Medicine, Baltimore, MD, USA; f Oxford Vaccine Group, Department of Paediatrics, University of Oxford and the National Institute for Health Research Oxford Biomedical Research Centre, Oxford, UK

## Abstract

**Background:**

Typhoid fever is a major cause of morbidity and mortality in low-income and middle-income countries. In 2017, WHO recommended the programmatic use of typhoid Vi-conjugate vaccine (TCV) in endemic settings, and Gavi, The Vaccine Alliance, has pledged support for vaccine introduction in these countries. Country-level health economic evaluations are now needed to inform decision-making.

**Methods:**

In this modelling study, we compared four strategies: no vaccination, routine immunisation at 9 months, and routine immunisation at 9 months with catch-up campaigns to either age 5 years or 15 years. For each of the 54 countries eligible for Gavi support, output from an age-structured transmission-dynamic model was combined with country-specific treatment and vaccine-related costs, treatment outcomes, and disability weights to estimate the reduction in typhoid burden, identify the strategy that maximised average net benefit (ie, the optimal strategy) across a range of country-specific willingness-to-pay (WTP) values, estimate and investigate the uncertainties surrounding our findings, and identify the epidemiological conditions under which vaccination is optimal.

**Findings:**

The optimal strategy was either no vaccination or TCV immunisation including a catch-up campaign. Routine vaccination with a catch-up campaign to 15 years of age was optimal in 38 countries, assuming a WTP value of at least US$200 per disability-adjusted life-year (DALY) averted, or assuming a WTP value of at least 25% of each country's gross domestic product (GDP) per capita per DALY averted, at a vaccine price of $1·50 per dose (but excluding Gavi's contribution according to each country's transition phase). This vaccination strategy was also optimal in 48 countries assuming a WTP of at least $500 per DALY averted, in 51 with assumed WTP values of at least $1000, in 47 countries assuming a WTP value of at least 50% of GDP per capita per DALY averted, and in 49 assuming a minimum of 100%. Vaccination was likely to be cost-effective in countries with 300 or more typhoid cases per 100 000 person-years. Uncertainty about the probability of hospital admission (and typhoid incidence and mortality) had the greatest influence on the optimal strategy.

**Interpretation:**

Countries should establish their own WTP threshold and consider routine TCV introduction, including a catch-up campaign when vaccination is optimal on the basis of this threshold. Obtaining improved estimates of the probability of hospital admission would be valuable whenever the optimal strategy is uncertain.

**Funding:**

Bill & Melinda Gates Foundation, Research Foundation–Flanders, and the Belgian–American Education Foundation.

## Introduction

Typhoid fever is estimated to cause 11·9–17·8 million cases globally each year, leading to approximately 130 000 deaths annually, mainly in low-income and middle-income countries (LMICs).^1^, ^2^, ^3^ Safe water and sanitation interventions are crucial to prevent the spread of typhoid fever, but such infrastructure is underdeveloped in LMICs.^4^ During the past two decades, the global incidence of typhoid fever has slowly declined.^1^ However, outbreaks of antimicrobial-resistant strains lasting several years threaten to undermine this progress.^5^

New developments might offer an opportunity to decrease the substantial burden posed by typhoid fever. Typhoid Vi-conjugate vaccines (TCVs) have been shown to be safe and immunogenic in infants as young as 6 months and hence can be added into existing childhood immunisation programmes in LMICs.^6^ Furthermore, TCVs are expected to have considerably higher efficacy and duration of protection than previous vaccines.^7^, ^8^, ^9^ In October 2017, WHO's Strategic Advisory Group of Experts recommended TCVs for routine use in countries where typhoid fever is endemic.^10^ Shortly thereafter, Gavi, The Vaccine Alliance pledged US$85 million to support the introduction of TCVs during the 2019–20 funding window.^11^ Finally, in January 2018, WHO announced the prequalification of the first Vi-conjugate vaccine against typhoid fever (Typbar-TCV, Bharat Biotech, India).^12^

Country-level policy makers are faced with the decision of whether to apply for Gavi support to implement a TCV programme. We used a prospective, model-based approach to evaluate the cost-effectiveness of universal vaccination of infants against typhoid fever with and without a catch-up campaign for 54 countries eligible for Gavi funding. We used country-specific data, where available, and evaluated uncertainties, including the impact of antimicrobial resistance on disease burden, treatment costs, and case-fatality rates. Our assessment provides practical guidance to clinicians and policy makers on the value and optimal design of a national TCV programme, as well as the performance standards necessary for TCVs to be cost-effective.

**Research in context**

**Evidence before this study**

Typhoid fever is a major cause of morbidity and mortality in low-income and middle-income countries. Newly developed typhoid Vi-conjugate vaccines (TCVs) are expected to have considerably higher efficacy and duration of protection than previous vaccines and are compatible with existing childhood immunisation programmes. Two health economic evaluations of TCVs found that routine immunisation, including a catch-up campaign, is likely to be cost-effective in settings with high incidence of typhoid fever. However, the optimal vaccination strategies differed for settings with medium incidence, and neither study was based on the most up-to-date information about vaccine costs and Gavi support, nor did it evaluate the cost-effectiveness of TCVs for individual countries.

**Added value of this study**

To our knowledge, ours is the first study to evaluate the cost-effectiveness of TCV strategies at the country level for all Gavi-eligible countries. We identify the optimal strategy (defined as the strategy that maximises the average net benefit) for each country and for a wide range of willingness-to-pay WTP values. We thereby provide specific information needed for each country to decide if and how to introduce a TCV vaccination programme. Rather than relying on a single or a few studies, we collate and describe in detail (using meta-analysis where appropriate) all available evidence on typhoid disease and economic burden for Gavi-eligible countries, including available evidence on hospital admission rates, typhoid treatment costs, vaccine delivery costs, length of stay in hospital, and duration of illness. For each country, we estimate uncertainty about the optimal strategy. We identify the typhoid hospital admission rate as a key area for further research to inform decisions on vaccine introduction. We identify the minimum typhoid incidence, case fatality, and hospital admission rate for any TCV strategy to be optimal when compared with no vaccination in each country, which can be used to guide policy decisions as additional evidence becomes available.

**Implications of all the available evidence**

The introduction of routine immunisation with TCV along with a catch-up campaign in children aged less than 15 years could be a cost-effective solution to combat the burden of typhoid fever, especially in countries with high typhoid incidence receiving Gavi support. TCV introduction should be considered when it is optimal on the basis of the country-specific WTP threshold. Gathering additional information on the probability of hospital admission (and on typhoid incidence and case-fatality rate) could help to resolve uncertainty in the optimal strategy.

## Methods

### Study design

For each of the 54 countries eligible for Gavi funding in 2016, we combined outputs from an age-structured transmission-dynamic model^13^ with data on use of economic resources to predict the incremental costs and disability-adjusted life-years (DALYs) averted by different TCV strategies compared with no vaccination.

We evaluated three TCV delivery strategies based on WHO's recommendation:^12^ routine vaccination alone with one dose of TCV at 9 months of age or combined with a catch-up campaign to either age 5 years or age 15 years. We considered a 10-year time horizon (2019–28) for our primary analysis, which incorporates the main impact of vaccination, and a 30-year time horizon in a sensitivity analysis. Costs and health outcomes were discounted at a rate of 3% for the economic analysis, according to the Bill & Melinda Gates Foundation Reference Case.^14^

Our main goal for informing policy was to identify the optimal strategy in terms of cost-effectiveness (ie, the strategy with highest average net benefit) for each country (appendix p 42). Because of the absence of a generally agreed-on willingness-to-pay (WTP) threshold to define whether an intervention is optimal in a given country,^15^ we identified for each country the optimal strategy for a range of WTP values (appendix p 42) and identified the minimum WTP value for which any vaccination strategy becomes optimal. Additionally, we investigated reasons for differences in the optimal strategy between countries and estimated and investigated the uncertainties surrounding our findings.

### Transmission-dynamic model

We used a previously published dynamic model^13^ of typhoid transmission to predict country-specific and age-specific numbers of typhoid cases with and without vaccination. By modelling both the incidence of clinical disease and transmission of infection, the model captures both the direct and indirect effects of vaccination, while accounting for important features of typhoid natural history, including the development of immunity and the chronic carrier state (table 1, appendix p 10). On the basis of each country's demographic profile, we sampled from transmission parameters to generate age-specific incidence profiles that reflect the uncertainty in typhoid incidence and the average age of patients, on the basis of the two most recently available estimates^1^, ^3^ (appendix p 14). We then simulated the annual number of symptomatic typhoid cases for six age groups (0 to <9 months, 9 months to <2 years, 2 to <5 years, 5 to <15 years, 15 to <25 years, and ≥25 years) in the absence of vaccination and for each of the three TCV delivery strategies. For each country and intervention strategy, we simulated the incidence 2000 times, while sampling from the posterior distributions of the model parameters to account for uncertainty in typhoid incidence and vaccine protection (table 1, appendix p 21).^1^, ^3^, ^18^ For typhoid natural history parameters (eg, duration of infectiousness and immunity), TCV efficacy and waning, we used the same estimates for all countries, as these parameters are unlikely to differ substantially between countries.

**Table 1:** Input parameters of the transmission-dynamic model

	**Level**	**Mean value and uncertainty distribution**	**Source**
**Demographic parameters**			
Crude birth rate (births per year)	Country-specific	15–36 livebirths per 1000 person-years (fixed, but varies by country)	Based on the demographic profile of the population (appendix pp 12–13)
Crude death rate (deaths per year)	Country-specific	Adjusted to maintain a constant population size	Based on the demographic profile of the population (appendix pp 12–13)
**Fixed disease parameters**			
Duration of infectiousness (1/δ)	Common	4 weeks, exponential	Based on Hornick et al (1970)^16^ (appendix p 10)
Fraction infected who become chronic carriers (θ)	Common	Fixed, but varies by age: <25 years=0·003 and ≥25 years=0·021	Based on Ames and Robins (1943)^17^ (appendix p 10)
Duration of immunity (1/ω)	Common	104 weeks, exponential	Based on Hornick et al (1970)^16^ (appendix p 10)
**Disease parameters with a-priori distributions**			
Relative transmission rate for children aged 0–2 years (β_1_/β_0_)	Common	0·37, beta (a=0·79, b=1·36)	A random sample from the parameters for the five sites modelled in Antillón et al (2017)^18^
Relative transmission rate for children aged 2–5 years (β_2_/β_0_)	Common	0·68, beta (a=1·55, b=0·72)	A random sample from the parameters for the five sites modelled Antillón et al (2017)^18^
Relative infectiousness of chronic carriers (r)	Common	0·25, beta (a=6·34, b=19·4)	Estimated to reproduce the indirect protection observed in a cluster-randomised trial of Vi-polysaccharide vaccine, as in Antillón et al (2017)^18^
**Vaccine-related characteristics**			
Initial efficacy of TCV (ν)	Common	87·5%, uniform (80–95%)	Based on Jin et al (2017)^7^ and Voysey et al (2018)^8^ (appendix p 21)
Duration of vaccine-induced immunity (ω_V_)	Common	15 years, uniform (10–20 years)	Re-analysis of Vi-rEPA data from Mai et al (2003)^9^ and half the estimated duration as a lower bound (appendix p 21)
Vaccine coverage for routine immunisation (κ_r_) and catch-up campaigns (κ_c_)	Country-specific	Fixed (appendix pp 18–21)	Gavi demand forecasts under the assumption of unconstrained supplies (appendix pp 16–17)

For each parameter are listed: (1) the level at which it is estimated (country-specific or common for all countries), (2) its estimated mean and uncertainty distribution, and (3) the source data or information used. TCV=typhoid Vi-conjugate vaccine. Vi-rEPA=Vi capsular polysaccharide conjugated to recombinant exoprotein A from *Pseudomonas aeruginosa*.

### Economic model input and assumptions

The economic model then multiplied the predicted number of typhoid cases with and without vaccination by estimates of the unit costs of treatment and vaccination and by estimates of the case-fatality rate and disability weights to calculate the incremental costs, number of deaths, years of life lived with disability (YLL), years of life lost due to death from typhoid fever (YLD), and DALYs over the time horizon of the analysis. All input parameters, their estimated values and uncertainty, and references to the data and statistical models on which they are based are listed in table 2 and detailed in the appendix (p 22). We did our economic evaluation from the health-care provider perspective, including only direct medical costs. Costs were denominated in 2016 US$. In the primary analysis, we included the vaccine price and delivery costs to be paid by each country on the basis of their expected graduation from Gavi support (appendix pp 35–39). In the scenario analysis, we included the full vaccine price of $1·50.^12^ Estimates of country-specific vaccine coverage were based on Gavi's demand forecast (appendix pp 18–20). We identified country-specific data on case-fatality rates (eight countries),^19^ hospital admission rates (four countries),^20^ and costs of vaccine delivery for routine (four countries) and campaign doses (13 countries).^26^, ^27^, ^28^ For countries without such data, we did random-effects meta-analyses, using all these available studies to inform common estimates (appendix p 22). Country-specific data on typhoid treatment costs were available for three countries;^21^, ^22^, ^23^ for all other countries, we estimated the treatment cost (per patient with typhoid fever) on the basis of WHO-CHOICE data (table 2, appendix pp 30–35).^24^ We used the same estimates for all countries for the parameters used to calculate DALYs. The probability of seeking professional medical care was informed by a model-based estimate^3^ of relative incidence from passive versus active surveillance.

**Table 2:** Input parameters of the economic model

	**Level of estimation and value median (mean) [95% CI] or assumption**	**Source or assumptions**
**Typhoid incidence and age distribution (**appendix p 10**)**		
Annual number of symptomatic typhoid fever cases per 100 000 people	By country (appendix p 22)	Based on Vos et al (2016),^1^ Antillon et al (2017),^3^ and Pitzer et al (2014);^13^ output from the transmission-dynamic model-fit to incidence of typhoid cases and mean age of patients
Average age of patients with typhoid infection	By country (appendix p 15)	Based on Vos et al (2016),^1^ Antillon et al (2017),^3^ and Pitzer et al (2014)^13^
**Typhoid mortality (**appendix p 24**)**		
Probability of death if patients are admitted to hospital for typhoid infection	By country for eight countries* (appendix p 24); common for 46 countries, 0·059 (0·044) [0·008–0·20]	Based on Pieters et al (2018);^19^ common estimate and prediction interval based on random-effects meta-analysis including 21 data points; country-specific estimates based on a random-effects meta-analysis including only data from those countries; SE was doubled for countries with only one study†
Proportion of deaths from typhoid infection occurring in patients not hospitalised for typhoid	Common for all countries, 0·38 (0·38) [0·022–0·73]	Assuming that on average about one of three deaths occur outside the hospital setting
Average age at death from typhoid infection	By country	Assuming age distribution of deaths is the same as the age distribution of patients with typhoid
**Antimicrobial resistance**		
Proportion of patients with typhoid infection with an AMR strain	Common for all countries, 0·5 (0·5) [0·024–0·98]	Assuming that 50% of cases are caused by infection with an AMR strain^3^
Burden of AMR cases relative to antimicrobial-sensitive cases	Common for all countries, 2 (2) [1–3]	Assumption; multiplication factor applied to average treatment cost, average years of life lived with disability, and average probability of death for hospitalised patients
**Health-care use (**appendix pp 25–29**)**		
Probability of infected patients seeking health care	Common for all countries, 0·57 (0·58) [0·42–0·77]	Based on Antillon et al (2017);^3^ based on estimate of relative incidence for passive *vs* active surveillance
Probability that infected patients are admited to hospital	By country for four countries‡ (appendix p 28); common for 50 countries, 0·038 (0·061) [0·004–0·25]	Based on Mogasale et al (2016);^20^ common estimate and prediction interval based on random-effects meta-analysis including nine data points
Length of stay in hospital (days)	By country for India, 7 (7) [4–11]; and Tanzania, 5 (5) [2–8]; common for 52 countries, 6 (6) [3–9]	Based on Sur et al (2009)^21^ and Riewpaiboon et al (2014);^22^ common estimate based on a random-effects meta-analysis; SE was doubled when extrapolating from a single setting to the country (for India and Tanzania)†
Number of visits to a medical doctor by inpatients and outpatients	Common for all countries=1	Fixed, assuming that the costs of a clinical visit are low and therefore unlikely to significantly affect the results
**Treatment costs (2016 US$;**appendix p 30**)**		
Cost of inpatient treatment	By country (appendix p 33)	India, pooled mean and SE based on two studies;^21^, ^23^ Tanzania^22^ and Pakistan,^23^ SE was doubled because of extrapolating from a single setting to the country;† for the other 51 other countries cost equals the cost per bed-day × length of stay in hospital + cost of laboratory tests per inpatient + cost of drugs per inpatient + cost outpatient visit × adjustment factor (appendix p 30)
Cost of outpatient treatment	By country (appendix p 33)	India, pooled mean and SE based on two studies;^21^, ^23^ Tanzania,^22^ SE was doubled because of extrapolating from a single setting to the country;† Pakistan;^23^ for the other 51 countries cost equals the cost of drugs per outpatient + cost of outpatient visit × adjustment factor (appendix p 30)
Cost of treatment for a patient not seeking professional medical care	By country for India, $1·4 (1·4) [0·9–2·1]; common for 53 countries, $0·81 (0·68) [0·039–2·28]	India,^21^ SE doubled because of extrapolating from a single setting to the country and from a country to another country;† for the 53 other countries equivalent to cost of drugs per outpatient
Unit cost per bed-day for inpatients and unit cost per outpatient visit	By country (appendix p 31)	WHO-CHOICE 2010,^24^ primary-level hospital for inpatients and health-care centre (no beds) for outpatients; SE was doubled because we use the unit cost for any disease as a proxy for the unit cost for typhoid fever†
Relative adjustment factor for overestimation of the unit cost per outpatient visit	Common for 51 countries, 0·63 (0·63) [0·25–1]	Assumption based on comparison with published data (appendix p 32)
Cost of drugs per inpatient	Common for 51 countries, $8·3 (12·6) [0·3–50·8]	Based on Sur et al (2009);^21^ SE was quadrupled because we extrapolate from one setting to the country and from one country to all other countries†
Costs of laboratory tests per inpatient	Common for 51 countries, $0·2 (6·9) [0–60·0]	Based on Sur et al (2009);^21^ SE was quadrupled because we extrapolate from one setting to the country and from one country to all other countries†
Cost of drugs per outpatient	Common for 51 countries, $0·81 (0·68) [0·039–2·28]	Based on Sur et al (2009)^21^ and Poulos et al (2011);^23^ SE was quadrupled because we extrapolate from one setting to the country and from one country to all other countries;† we then multiplied by a uniform distribution between 0 and 1 on the basis of Poulos et al (2011)^23^ who reported no costs of outpatient treatment (appendix p 32)
Costs of laboratory tests per inpatient	Common for 51 countries, $0·2 (6·9) [0–60·0]	Based on Sur et al (2009);^21^ SE was quadrupled because we extrapolate from one setting to the country and from one country to all other countries†
Costs of laboratory tests per outpatient	Common for 51 countries, $0	Based on Sur et al (2009);^21^ none of the 67 outpatients had laboratory tests reported
**Vaccine-related costs (per dose, 2016 US$) (**appendix p 35**)**		
Vaccine procurement	By country; $1·5 minus Gavi support (appendix p 35)	Based on Bharat price announcement and personal communication with Gavi
Injection and safety equipment	Common for all countries, $0·23 (0·23) [0·21–0·24]	Based on Portnoy et al (2015);^25^ assuming reported minimum ($0·21) and maximum ($0·24) cost reflect 95% confidence limits of the gamma distribution and distribution is symmetric around the mean
Routine vaccine delivery cost per dose	By country for four countries (Benin, Ghana, Zambia, and Rwanda; appendix p 36) and by WHO region for 50 countries; Africa: $1·61 (1·76) [0·36–4·23]; southeast Asia: $1·40 (1·53) [0·31–3·68]; western Pacific: $0·72 (0·79) [0·16–1·90]; Europe: $3·52 (3·86) [0·78–9·27]; WHO Region of the Americas: $2·13 (1·95) [0·43–5·12]; east Mediterranean: $2·09 (2·29) [0·47–5·50]; GAVI support is subtracted from the estimated delivery cost (appendix pp 36–40)	Benin, Ghana, Zambia (EPIC studies, one data point by country), and Rwanda^26^ (two data points); to account for the uncertainty of using the costs of other vaccines (pneumococcal, rotavirus, and measles) as proxies for the Vi-conjugate typhoid vaccine, a SE of $0·45 is used, which equals the SE of the mean of the five data points; other 50 countries: mean of the five data points multiplied by adjustment factor by WHO region (EPIC studies, Ngabo et al [2015],^26^ and Atherly et al [2012]^27^); SE equals twice the SE assumed for Benin, Ghana, Zambia, and Rwanda because we extrapolated to other countries† (appendix p 36)
Number of years during which start-up costs of vaccine delivery programme are incurred	Common for all countries, 2 (2) [1–3]	Assumption beacause of unavailability of data
Routine vacine delivery costs (%)	By country for Benin, Ghana, Zambia, and Rwanda (appendix p 36); common for 50 countries, 64% (64) [48–78]	Benin, Ghana, Zambia (EPIC studies, one data point by country), and Rwanda^26^ (two data points); other 50 countries, mean of the five data points with SE of the mean doubled because we extrapolated to other countries†
Campaign vaccine delivery cost per dose	By country for 13 countries§ (appendix p 40); common for 41 countries, $0·40 (0·41) [0·23–0·62]; GAVI support is subtracted from the estimated delivery cost (appendix pp 36–40)	Based on Gandhi et al (2014);^28^ common for all 41 countries; SE was doubled because we extrapolated from the mean of the 13 countries to other countries†
**Disability-adjusted life-years (**appendix p 40**)**		
Disability weights from 0 (perfect health) to 1 (death)	Common for all countries; severe illness, 0·21 (0·21) [0·14–0·29]; moderate illness, 0·052 (0·053) [0·031–0·079]; mild illness, 0·005 (0·005) [0·002–0·011]	Based on Salomon et al (2012)^29^
Relationship between disability weights for mild, moderate, and severe illness and outcomes on health-care use	Common for all countries (appendix pp 40–41)	Assumption justified in appendix pp 40–41
Duration of illness in inpatients and outpatients (days)	Common for all countries, 16 (16) [12–20]	Based on Sur et al (2009),^21^ Riewpaiboon et al (2014),^22^ and Poulos et al (2011);^23^ SE based on the prediction interval of random-effects meta-analysis
Relative duration of illness for patients not seeking medical care (*vs* inpatients and outpatients)	Common for all countries, 0·5 (0·5) [0·02–0·98]	Assuming that individuals with typhoid fever not seeking care had an average duration of illness of 8 days (ie, half the length of illness of people who sought medical care), which varied between 0 and 16 days
Life expectancy at birth (years)	By country (appendix p 41)	World Bank 2014 datasheet

For each characteristic, we list: (1) the level at which a parameter is estimated (country, WHO region, common [ie, a single estimate for all countries]); (2) its estimated median, mean, and 95% credible interval; and (3) the source data or information that was used for the estimate. For the uncertainty distributions and how they were determined, see the appendix section starting at p 22. AMR=antimicrobial-resistant.

* Countries with typhoid mortality data: Bangladesh, Ethiopia, Côte d'Ivoire, Laos, and Zimbabwe (one study each); India (five studies); Nigeria (two studies); and Senegal (two studies). All references available in the appendix.

† The doubling or quadrupling of the SE was a pragmatic choice and was done to ensure that those parameters for which little evidence was available (eg, no country-specific data) were characterised by more uncertainty than the parameters for which ample evidence was available.

‡ Countries with data on hospital admission of patients with typhoid infection: Bangladesh, India, Kenya, and Pakistan.

§ Countries with vaccine delivery cost data for campaigns: Afghanistan, Burkina Faso, Côte d'Ivoire, Ethiopia, Guinea, Laos, Nigeria, Pakistan, Rwanda, Senegal, Uganda, Tanzania, and Zambia.

Uncertainty in the parameters of the economic model was quantified by assigning probability distributions to each input parameter. Parameters for which more information was available (eg, if there were several studies for a single country, information was available from the country itself, or information was available on the parameter of interest rather than a proxy) were characterised by less uncertainty than the parameters for which less information was available (table 2, appendix p 22). We used wide uncertainty ranges for the presence and burden of antimicrobial-resistant strains and the case-fatality rate outside a hospital setting because of the lack of sufficient (country-specific) data.

### Uncertainty analysis

We used a variety of methods to understand the robustness of our findings in the face of uncertainty in the underlying input data. First, we did a probabilistic sensitivity analysis to generate cost-effectiveness results by drawing 2000 random samples independently from the probability distributions of each uncertain input parameter (appendix p 42). Each random sample was combined with one of the 2000 samples from the transmission-dynamic model and then inputted in the economic model to calculate 2000 net monetary benefit values for each of the three vaccination strategies and for a range of WTP values. We quantified uncertainty about the optimal strategy in each country according to the percentage of samples for which the strategy yielded the highest net benefit at a given WTP (appendix p 42–43).

Second, we identified the most important drivers of the uncertainty surrounding the optimal vaccination strategy in each country: for each input parameter, we estimated the expected value of partially perfect information (EVPPI), an upper-bound measure on what countries might be willing to pay for research that would resolve all uncertainty surrounding a given variable (appendix p 43). Third, for each country, we used threshold analysis to determine the tipping point (ie, the value of typhoid incidence, probability of hospital admission, and case-fatality rate) at which the optimal strategy for a country would change from no vaccination to one of the three vaccination strategies considered. Finally, we conducted scenario analyses to investigate the influence of our assumptions regarding the timeframe, vaccine price, cost of vaccine delivery for routine versus campaign doses, and stability of typhoid transmission rates.

The transmission-dynamic model was done in MATLAB 2014b (version 8.4.0, MathWorks, Natick, MA, USA), and the economic model in R (version 3.0.2; appendix pp 43–44).^30^

### Role of the funding source

The funder had no role in study design, data collection, data analysis, data interpretation, or writing of the report. The corresponding author had full access to all the data in the study and had final responsibility for the decision to submit for publication.

## Results

**Figure 1:**
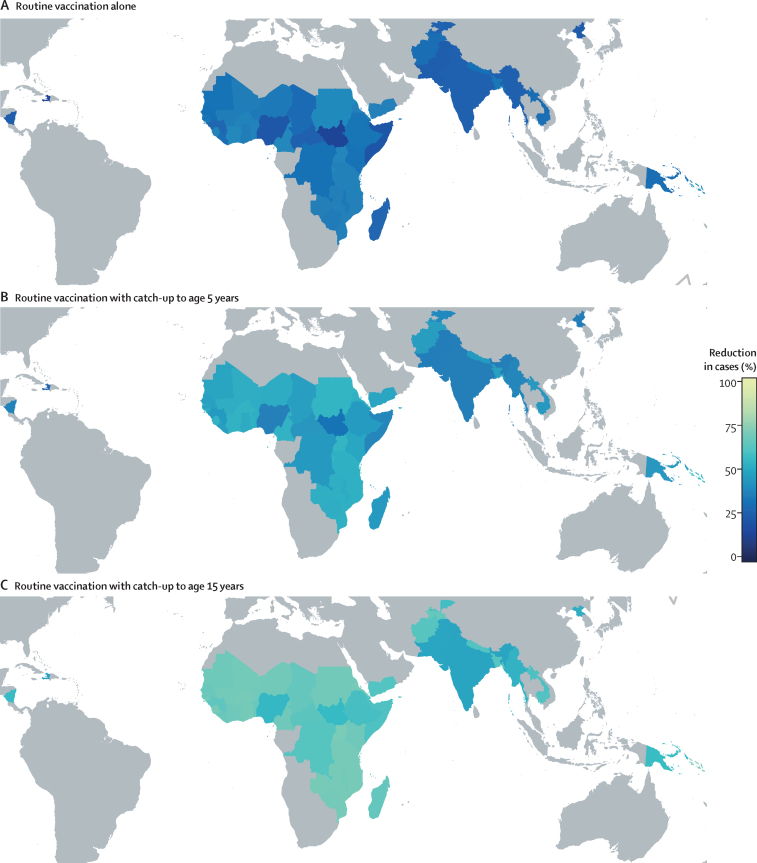
Predicted impact of typhoid Vi-conjugate vaccine use in Gavi-eligible countries Predicted percentage reduction in symptomatic typhoid cases over 10 years in 54 Gavi-eligible countries when introducing (A) routine vaccination at 9 months of age alone, (B) a routine vaccination programme with a catch-up campaign up to the age of 5 years, and (C) a routine vaccination programme with a catch-up campaign up to the age of 15 years, compared with no vaccination. Vaccine coverage in each country is based on the Gavi demand forecast. Results shown are not discounted.

The estimated number of typhoid cases, deaths, DALYs, and treatment costs in the absence of vaccination for each country are shown in the appendix (p 45). Routine vaccination alone was predicted to avert 33 million (95% prediction interval [PI] 16–59) cases of typhoid fever over 10 years, which represents a 30% (95% PI 15–43) decrease in incidence (figure 1). Routine vaccination with a catch-up campaign for children aged under 5 years was predicted to avert 47 million (95% PI 22–73) cases, whereas extending catch-up to under-15 year olds was predicted to avert 63 million (31–94) cases of typhoid fever over 10 years. The deaths, DALYs, and treatment costs averted, as well as the expected cost of implementing the vaccination programmes, are presented in the appendix (pp 48–55).

**Figure 2:**
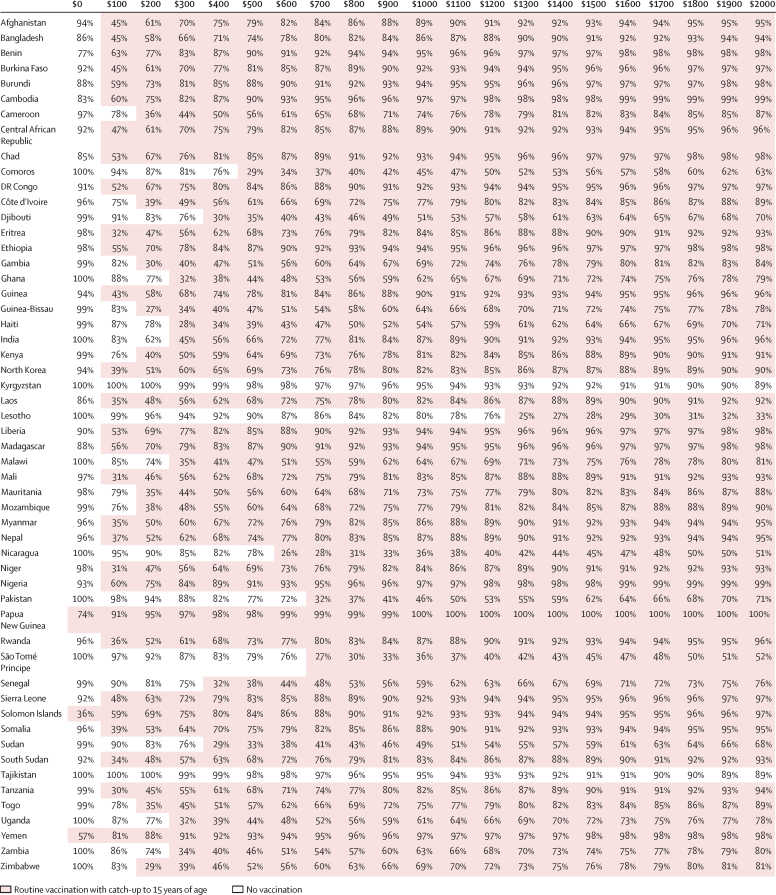
Optimal intervention strategy and its estimated certainty for each country for a range of willingness-to-pay values per disability-adjusted life-year averted The optimal strategy is defined as the strategy that yields the highest average net monetary benefit and hence is preferable over the three other strategies on the basis of cost-effectiveness alone. Shading shows the preferred strategy: no vaccination (white) or routine immunisation with a catch-up campaign up to age 15 years (shaded). The percentages indicate certainty about the optimal strategy, estimated by the percentage of parameter samples in which the strategy yielded the highest net benefit. The degree of uncertainty influences the value of obtaining more evidence to make a future decision but should not influence the choice of strategy given the current evidence.

**Figure 3:**
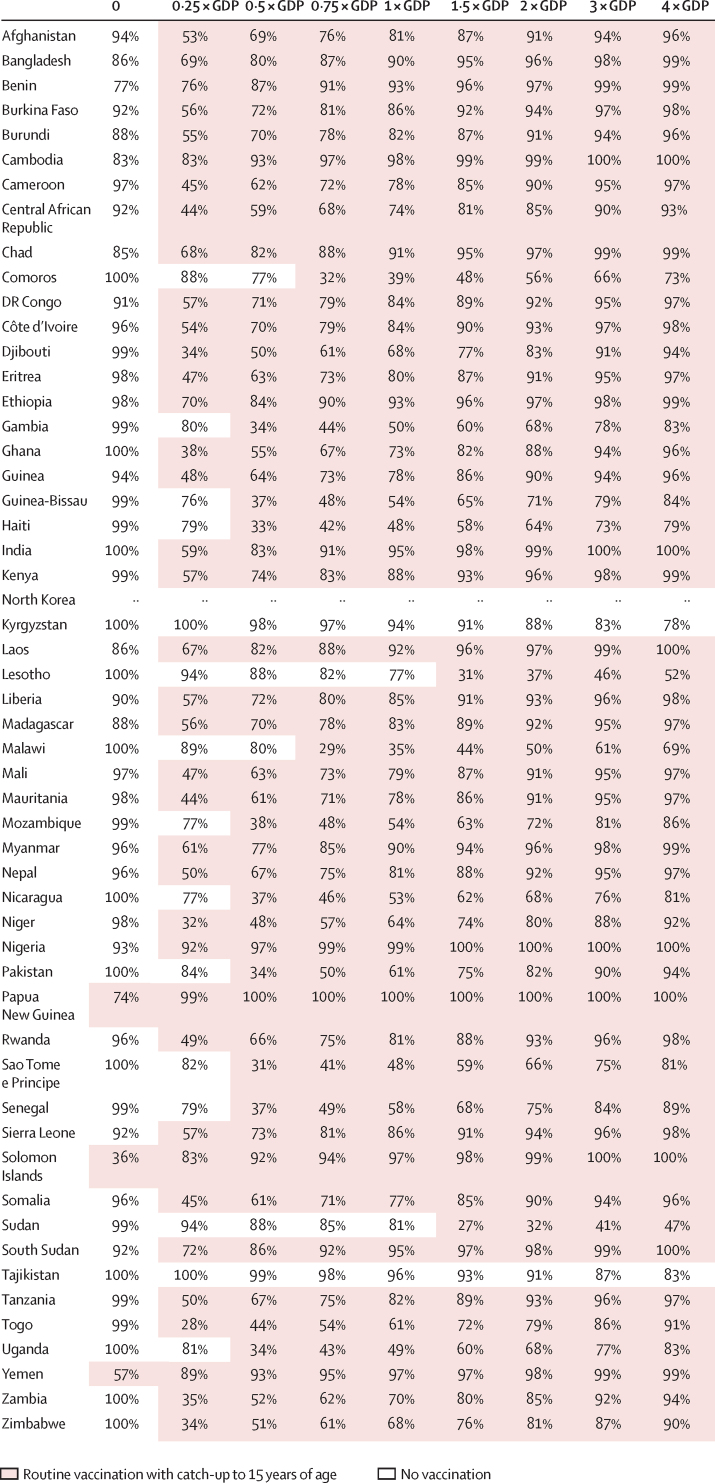
Optimal intervention strategy and its estimated certainty for each country for a range of willingness-to-pay values (0–4 times GDP per capita per disability-adjusted life-year averted) The optimal strategy is defined as the strategy that yields the highest average net monetary benefit and hence is preferable over the three other strategies on the basis of cost-effectiveness alone. Shading shows the preferred strategy: no vaccination (white) or routine immunisation with a catch-up campaign up to age 15 years (shaded). Certainty is indicated by the percentage of parameter samples in which the strategy yielded the highest net benefit. The degree of uncertainty influences the value of obtaining more evidence to make a future decision but should not influence the choice of strategy given the current evidence. No gross domestic product per capita was available for North Korea. GDP=gross domestic product.

Our results indicated that TCV introduction with an initial catch-up campaign was always optimal when compared with routine immunisation alone (Figure 2, Figure 3, and appendix pp 67–80). Routine vaccination with catch-up for under-15 year olds was optimal in 38 countries when the WTP was equal to or higher than $200 per DALY averted, in 48 countries at a WTP of at least $500, and in 51 countries at a WTP of $1000 or more (figure 2). For two countries (Kyrgyzstan and Tajikistan), none of the vaccination programmes were optimal compared with no vaccination at WTP values of less than $5500 per DALY averted. In terms of gross domestic product (GDP) per capita, routine vaccination with a catch-up campaign for children under 15 years of age was optimal in 38 countries at WTP values of at least 25% of GDP per capita per DALY averted, in 47 countries at a WTP of 50% or more, and in 49 countries at a WTP of 100% or more (figure 3).

Countries for which TCV introduction was probably the optimal strategy had a high typhoid incidence (≥300 cases per 100 000 person-years; appendix p 57). Some of these countries also had either very high treatment costs (≥$115 per case for inpatients and ≥$2·50 per case for outpatients in the Solomon Islands and Papua New Guinea; appendix p 33) or an exceptionally high case-fatality rate in hospitalised patients (CFR_hosp_) based on data from those countries (0·16 for Nigeria and 0·15 in Ethiopia; appendix p 24). Nevertheless, when we assumed the common estimate of CFR_hosp_ for Nigeria and Ethiopia, the minimum WTP value at which vaccination becomes optimal did not change (appendix p 82).

TCV introduction is unlikely to be optimal in countries with low estimated incidence of typhoid fever (appendix p 57); for example, in Kyrgyzstan, Lesotho, and Tajikistan, there were fewer than 30 symptomatic cases per 100 000 person-years (appendix p 15). Kyrgyzstan and Tajikistan also had a high estimated average age of infection (≥16 years, appendix p 15) and a high vaccine delivery cost for routine immunisation ($3·86 per dose; appendix p 37).

We estimated that routine vaccination including a catch-up for children aged up to 15 years was at least 50% certain to be the optimal strategy in 22 countries when assuming a WTP value of $200 per DALY averted, 38 when assuming a WTP of $500, and 46 when WTP was assumed to be $1000. This strategy was at least 80% certain to be optimal in two countries with assumed WTP of $200 per DALY averted, 14 when the WTP was $500, and 31 when WTP was $1000 (figure 2). In terms of GDP per capita, there was at least 50% certainty that it was optimal in 22 countries when assuming the WTP value to be 25% of GDP, in 35 when the WTP was 50%, and in 43 when WTP was equal to 100% of the GDP per capita per DALY averted (figure 3).

Uncertainty about the probability of hospital admission for typhoid contributes the most to the uncertainty about the optimal vaccination strategy in 52 countries (appendix p 57). In 14 of these 52 countries, uncertainty about the incidence of symptomatic typhoid cases was the second most important contributor to the uncertainty in the optimal vaccination strategy, whereas in 35 countries it was the absence of precise case-fatality rate estimates (appendix p 57). In India, the uncertainty about typhoid incidence contributed most to the uncertainty in choosing the optimal strategy, whereas in Pakistan it was the uncertainty around the CFR_hosp_. Appendix pp 61–66 show the tipping point values for the probability of hospital admission, incidence of typhoid, and CFR_hosp_, for which any vaccination strategy becomes optimal when compared with no vaccination.

In our scenario analyses, routine vaccination with catch-up for children up to 15 years of age remained the best strategy whenever vaccination was optimal, as suggested by the primary analysis, for 34 countries when all costs were included (ie, country contribution plus contribution from Gavi, appendix p 67). Routine vaccination with catch-up for children aged up to 5 years was optimal compared with routine vaccination alone and routine vaccination with catch-up for those aged up to 15 years for a small range of WTP values in 45 countries when delivery costs for campaign doses were assumed to be on average equal to those for routine doses (appendix p 71). Our results did not change substantially when assuming a 25% decline in the typhoid transmission rate over 10 years without vaccination (appendix p 75), nor when including costs and health benefits for 30 years instead of 10 years (appendix p 79).

## Discussion

Our results consistently showed that the best strategy when implementing routine TCV vaccination of infants is to include a catch-up campaign to age 15 years. At WTP thresholds of 25–100% of a country's GDP per capita per DALY averted, we found that TCV introduction was optimal in 38–49 countries, when including only direct medical costs and the country's funding contribution to vaccine implementation in countries eligible for support from Gavi (ie, $0·20–1·5 per dose, depending each country's transition phase).

For most countries, substantial uncertainty exists about the optimal vaccination strategy. Nevertheless, this uncertainty should not preclude the introduction of TCV. Decisions need to be made in the context of uncertainty, and the strategy of choice in terms of cost-effectiveness should be the one resulting in the highest expected net benefit (what we refer to as the optimal strategy), given a country's WTP value. Each country would benefit from establishing its own WTP threshold, reflecting local preferences. The choice of a WTP threshold is related to budget restrictions and local value judgments on efficiency–equity trade-offs and might be guided by the cost-effectiveness of previously funded and non-funded health-care technology to ensure consistency in policy making. Specific guidance on the use of our results to inform country-level decision-making is discussed in the appendix (pp 82–83).

By accounting for uncertainty in our analysis, we were able to identify the key parameters used in the model for which investing in obtaining more information could be valuable. Although we accounted for uncertainty in 23 variables, including very wide uncertainty ranges when little or no information was available, uncertainty around the probability of hospital admission for typhoid affected the optimal strategy more than any other variable in 52 countries. The probability of hospital admission was informed by data from seven sites in four countries; there was considerable heterogeneity between the sites that could not be explained by national, subnational, or site-specific differences. The incidence of symptomatic typhoid cases and the case-fatality rate among inpatients also contributed to uncertainty on the preferred strategy in 45 countries for the WTP values we considered. The incidence estimates we obtained are extrapolated from a small number of population-based studies and adjusted for the probability of seeking care and the poor sensitivity of blood culture to detect typhoid infection.^3^, ^31^

Improving the precision of estimates of typhoid incidence, probability of hospital admission, and case-fatality rate is not straightforward. Symptoms of typhoid fever are non-specific and can be confused with numerous other febrile illnesses. Proper diagnosis relies on blood culture, which requires laboratory facilities that are not available in many LMICs. Commonly used serological tests (eg, Widal test) have poor specificity. These difficulties underscore the need for better diagnostics, as well as potential proxies for the incidence of typhoid fever, such as environmental surveillance and cross-sectional serosurveys.^32^ Furthermore, typhoid incidence varies over both space and time. The probability for hospital admission and case fatality are also very heterogeneous^19^ and are difficult to assess without confirming cases through laboratory culture. Because precise incidence, hospital admission, and mortality rate estimates are difficult to obtain, we identified the minimum values at which TCV vaccination becomes optimal compared with no vaccination in each country, which could be helpful in identifying priority regions to target for vaccination.

The large uncertainty in the prevalence and relative burden of antimicrobial-resistant infections had only a minor effect on the optimal strategy. The insensitivity of our results to the uncertainty in these parameters might be because the prevalence and burden of antimicrobial-resistant typhoid fever were modelled as conditional on overall incidence and mortality. However, the emergence of antimicrobial-resistant strains, which are potentially more transmissible, could lead to higher incidence, probability of hospital admission, and case fatality, and thereby influence the cost-effectiveness of TCVs.^33^ The threat of antimicrobial-resistant typhoid fever, such as the outbreak of an extensively drug-resistant strain in Pakistan in November 2016,^5^ is another powerful impetus for those trying to control the epidemic and prevent the spread of resistance genes to other pathogens.

The efficacy and duration of protection of vaccines also did not contribute substantially to uncertainty around the optimal vaccination strategy. In the absence of effectiveness data for Typbar-TCV, we assumed efficacy to be equal to the seroefficacy from an immunogenicity trial^8^ with Typbar-TCV (80–95%), which was similar to the efficacy of a previous TCV candidate.^9^ Further supporting our assumption is a human challenge study,^7^ in which efficacy of the vaccine against primary infection was only 55%, whereas the efficacy against a more clinically relevant endpoint (fever ≥38°C followed by a positive blood culture) was around 85%. The results of ongoing field trials will show if we have potentially underestimated the uncertainty and overestimated the average efficacy and duration of protection for TCVs.

Our results are generally consistent with previous health economic evaluations of TCV delivery strategies.^18^, ^34^ However, Lo and colleagues^34^ found that routine vaccination alone was a more cost-effective strategy than routine vaccination with a catch-up campaign in school-aged children in medium-incidence settings. The discrepancy with our conclusion might be due to several reasons, including differences in the analytical approach, the delivery strategies assessed, and assumptions about delivery costs for routine versus campaign doses (appendix p 83). Perhaps most importantly, Lo and colleagues^34^ might have overestimated the incidence of typhoid fever in children younger than 5 years in medium-incidence settings. This error would make routine vaccination more attractive. We found that in most medium-incidence settings there were fewer cases in this age group,^35^, ^36^ and therefore estimated a lower relative risk for children younger than 2 years and those aged 2 to less than 5 years.

Our study has several limitations. First, our analysis focused only on vaccination strategies against typhoid fever. We were not able to compare these directly to other interventions (eg, safe water and sanitation) because such analysis would require a different type of model, and input on the costs and impact of safe water and sanitation interventions on typhoid fever outcomes are unavailable. Nevertheless, TCV programmes should be integrated with interventions aimed at providing safe water and sanitation, as these are likely to have benefits that extend beyond possible reductions in typhoid incidence. Furthermore, we did not consider targeted vaccination of high-risk groups because the feasibility of such a strategy is questionable and could be perceived as less equitable. However, our analysis of the minimum incidence of typhoid fever at which any TCV strategy becomes optimal in each country could be used in combination with sub-national data on heterogeneity of incidence to guide spatially targeted vaccination strategies.

Second, for many countries, data on typhoid disease and economic burden were unavailable; hence, we used model-derived estimates of typhoid incidence and treatment costs and directly estimated case-fatality rates and vaccine delivery costs for a subset of countries. Also, we accounted for uncertainty in ways that acknowledge that more evidence is available for some countries, and hence the optimal vaccination strategy can be established with varying degrees of certainty.

Third, we evaluated the cost-effectiveness of TCVs from the health-care provider perspective. When including additional benefits from the societal perspective, such as preventing the loss of school hours or work in patients with typhoid fever and their caregivers, our results would probably be better in favour of TCV programmes.

WHO's policy recommendation and financial support from Gavi provide resource-limited countries with a realistic opportunity to control typhoid fever through vaccination. Policy makers now need to decide whether to invest in a TCV programme in their country. Budgetary constraints will need to be considered, along with the feasibility and acceptability of incorporating TCVs into existing immunisation programmes. Nevertheless, our analysis shows that for many of the populations most severely affected by typhoid, Vi-conjugate vaccines offer a cost-effective solution.

## Data sharing

The data for this study can be accessed on the Cost-effectiveness of typhoid fever vaccination website https://ceatyphoid.uantwerpen.be/home/
