# Correction to *Lancet Infect Dis* 2019; 19: 728–39

**DOI:** 10.1016/S1473-3099(20)30259-0

**Published:** 2020-05

*Bilcke J, Antillón M, Pieters Z, et al. Cost-effectiveness of routine and campaign use of typhoid Vi-conjugate vaccine in Gavi-eligible countries: a modelling study.* Lancet Infect Dis *2019;* 19: *728–39*—In this Article, the penultimate row of table 1 has been corrected to reflect that the mean value and uncertainty distribution of waning of vaccine-induced immunity (ωV) are 0·0672 per year, gamma (a=1·38, b=0·0481). The Appendix of the Article has also been corrected. These corrections have been made to the online version as of March 30, 2020.

