# Supplementary material for: Cost-effectiveness of routine and campaign use of typhoid Vi-conjugate vaccine in Gavi-eligible countries: a modelling study
Source: Lancet Infect Dis. 2019 Jul;19(7):728–39. doi: 10.1016/S1473-3099(18)30804-1 (PMC6595249; doi:10.1016/S1473-3099(18)30804-1)
Supplement: Supplementary appendix [file mmc1.pdf]

THE LANCET Infectious Diseases

Supplementary webappendix

This webappendix formed part of the original submission and has been peer reviewed. We post it as supplied by the authors.

Supplement to: Bilcke J, Antillón M, Pieters Z, et al. Cost-effectiveness of routine and campaign use of typhoid Vi-conjugate vaccine in Gavi-eligible countries: a modelling study. *Lancet Infect Dis* 2019; published online May 23. http://dx.doi.org/10.1016/S1473-3099(18)30804-1.

Cost-effectiveness of routine and campaign use of typhoid Vi-conjugate vaccine in Gavi-eligible countries: a modelling study

Bilcke J (PhD)[1], Antillón M (PhD)[1,2], Pieters Z (MS)[1,3], Kuylen E (MS)[1], Abboud L (MS)[1], Prof Neuzil KM (MD)[4], Prof Pollard AJ (FMedSci)[5], Prof Paltiel AD (PhD)[6], & Pitzer VE (ScD)[2]

[1]Centre for Health Economics Research and Modeling Infectious Diseases (CHERMID), Vaccine and Infectious Disease Institute (VAXINFECTIO), University of Antwerp, Universiteitsplein 1, 2610 Wilrijk, Belgium

[2]Department of Epidemiology of Microbial Diseases, Yale School of Public Health, Yale University, P.O. Box 208034, 60 College St, New Haven, CT 06520-8034

[3]Center for Statistics, I-Biostat, Hasselt University, Agoralaan, 3590 Diepenbeek, Belgium

[4]Center for Vaccine Development and Global Health, University of Maryland School of Medicine, 655 W. Baltimore St, Baltimore, MD 21201

[5]Oxford Vaccine Group, Department of Pediatrics, University of Oxford and the NIHR Oxford Biomedical Research Centre, Oxford, UK OX3 7LE

[6]Department of Health Policy & Management, Yale School of Public Health, Yale University, P.O. Box 208034, 60 College St, New Haven, CT 06520-8034

Corresponding author:
Joke Bilcke
D.R2.11
University of Antwerp
Universiteitsplein 1
2610 Wilrijk (Antwerp)
Belgium
joke.bilcke@uantwerp.be
+32/32652895

1 Table of Contents

1 TABLE OF CONTENTS 2

1.1 LIST OF TABLES 4
1.2 LIST OF FIGURES 8

2 METHODS 10

2.1 TRANSMISSION-DYNAMIC MODEL 10
2.1.1 DESCRIPTION OF THE DYNAMIC MODEL 10
2.1.2 BASIC REPRODUCTION NUMBER (R_0) 12
2.1.3 VACCINATION 12
2.1.4 ESTIMATING COUNTRY-SPECIFIC PARAMETERS FOR THE DYNAMIC MODEL 12
2.1.5 SIMULATING TYPHOID INCIDENCE IN THE CONTEXT OF VACCINATION 16
2.2 INPUT PARAMETERS FOR COST-EFFECTIVENESS ANALYSIS 22
2.2.1 OUTPUT FROM TRANSMISSION-DYNAMIC MODEL 22
2.2.2 TYPHOID MORTALITY 24
2.2.3 HEALTHCARE USE 25
2.2.4 ANTIMICROBIAL RESISTANCE 29
2.2.5 COSTS CONVERSION AND INFLATION 30
2.2.6 TREATMENT COSTS 30
2.2.7 VACCINE-RELATED COSTS 35
2.2.8 DISABILITY-ADJUSTED LIFE-YEARS 40
2.3 COST-EFFECTIVENESS ANALYSIS 42
2.3.1 DETERMINING THE OPTIMAL STRATEGY FOR EACH COUNTRY 42
2.3.2 PROBABILISTIC SENSITIVITY ANALYSIS ('PSA') 42
2.3.3 DETERMINING THE UNCERTAINTY AROUND THE OPTIMAL STRATEGY FOR EACH COUNTRY 42
2.3.4 IDENTIFYING THE KEY DRIVERS OF UNCERTAINTY WITH EVPPI 43
2.3.5 IDENTIFYING MINIMUM CONDITIONS FOR VACCINATION TO BE OPTIMAL WITH THRESHOLD ANALYSIS 43

3 RESULTS 45

3.1 PREDICTED DISEASE AND ECONOMIC BURDEN IN THE ABSENCE OF VACCINATION 45
3.2 PREDICTED IMPACT OF VACCINATION 48
3.3 COST-EFFECTIVENESS OF VACCINATION 57
3.3.1 WHAT MAKES VACCINATION OPTIMAL IN SOME COUNTRIES BUT NOT IN OTHERS? 57
3.3.2 WHAT ARE THE KEY DRIVERS OF UNCERTAINTY (EVPPI)? 57
3.3.3 MINIMUM CONDITIONS UNDER WHICH VACCINATION IS OPTIMAL (THRESHOLD ANALYSIS) 59
3.4 SCENARIO ANALYSES 67
3.4.1 VACCINE COST: INCLUDING GAVI'S SHARE 67
3.4.2 VACCINE COST: INCLUDING GAVI'S SHARE AND ASSUMING THE DELIVERY COST OF CAMPAIGN DOSES IS THE SAME AS ROUTINE DOSES. 71
3.4.3 TYPHOID INCIDENCE: DECLINING OVER TIME WITHOUT VACCINATION. 75
3.4.4 TIME HORIZON: 30 YEARS 79
3.4.5 CASE FATALITY RATE IN HOSPITAL FOR NIGERIA AND ETHIOPIA. 82

4 INTERPRETATION OF RESULTS FOR POLICY-MAKING 82

5 DISCUSSION 83

5.1 COMPARISON WITH PREVIOUS HEALTH ECONOMIC EVALUATIONS 83

6 CHEERS CHECKLIST 85

1.1 List of Tables

Appendix Table 1: Age distribution of three demographic profiles according to the UN World Population Prospects database. 13
Appendix Table 2: Age distribution of three demographic profiles according to the UN World Population Prospects database, adjusted for the age groups from our model. 13
Appendix Table 3: Assumed country-specific vaccine coverage over time for routine immunisation when assuming unconstrained supplies. Coverage estimates for the years 11 up to 30 are available from the authors. 18
Appendix Table 4: Assumed country-specific vaccine coverage over time for campaign when assuming unconstrained supplies. 20
Appendix Table 5: Vaccine-related parameters used in the transmission-dynamic model. 21
Appendix Table 6: Country-specific baseline typhoid incidence (i.e. no vaccination) used in the economic model. Median and 95% credible interval are shown. 22
Appendix Table 7: Country-specific case fatality rates for hospitalized patients with typhoid fever used in our cost-effectiveness analysis. 24
Appendix Table 8: Description of studies included in the meta-analysis of the probability of hospitalization for typhoid fever (reprinted from Abboud Linda, University of Antwerp Master's thesis, 2017 [25]). 26
Appendix Table 9: Factors potentially associated with typhoid hospitalization rate (reprinted from Abboud Linda, University of Antwerp Master's thesis, 2017 [25]). .. 27
Appendix Table 10: Country-specific probability of hospitalization for typhoid fever used in our cost-effectiveness analysis. 28
Appendix Table 11: Cost per bed day and outpatient visit for *any disease* in a district/provincial or primary referral hospital. Comparison of costs based on the WHO-CHOICE prediction model and recent country-specific publications. Costs are presented in USD2010. 31
Appendix Table 12: Treatment costs for a typhoid fever episode in an inpatient and outpatient setting. Comparison of costs based on the WHO-CHOICE prediction model (for any disease) and typhoid-specific cost studies. Costs are presented in USD2010. 32
Appendix Table 13: Treatment costs for a complete typhoid fever episode in an inpatient and outpatient setting including the adjustment factor for the treatment cost of a typhoid outpatient. Comparison of costs based on the WHO-CHOICE prediction mode (for any disease) and typhoid-specific cost studies. Costs are presented in USD2010. 33
Appendix Table 14: Average treatment cost for a typhoid inpatient and outpatient for each Gavi-eligible country. We list the mean, median, and 95% credible interval (UI, lower and upper bound) of the treatment cost distributions used as input in our model. Costs presented in USD2016. 33
Appendix Table 15: Overview of the studies used to estimate the average delivery cost per dose for adding a new vaccine to an existing routine immunization program. Costs that are ongoing (as opposed to start-up) are presented separately. Costs are presented in USD2016. 36
Appendix Table 16: Vaccine delivery cost per dose for routine immunization and for a one-time catch-up campaign. Mean, median, and 95% credible intervals (UI, lower and upper bounds) for each country used as input in our model are presented. Costs presented in USD2016. 37
Appendix Table 17: Uncertainty distributions for country-specific vaccine delivery cost, as used in our cost-effectiveness analysis 40

Appendix Table 18: Median [95% credible interval] discounted burden, costs and DALYs in the absence of vaccination, based on a 10-year time horizon. Costs are presented in USD2016. Output is discounted at a rate of 3% per year. 45
Appendix Table 19: Median [95% credible interval] discounted avoided burden, incremental costs and DALYs averted for routine vaccination against typhoid fever compared to no vaccination, based on a 10-year time horizon. Costs are presented in USD2016. Output is discounted at a rate of 3% per year. 48
Appendix Table 20: Median [95% credible interval] discounted avoided burden, incremental costs and DALYs averted for routine vaccination against typhoid fever with a catch-up campaign up to 5 years of age compared to no vaccination, based on a 10-year time horizon. Costs are presented in USD2016. Output is discounted at a rate of 3% per year. 51
Appendix Table 21: Median [95% credible interval]discounted avoided burden, incremental costs and DALYs averted for routine vaccination against typhoid fever with a catch-up campaign up to 15 years of age compared to no vaccination, based on a 10-year time horizon. Costs are presented in USD2016. Output is discounted at a rate of 3% per year. 54
Appendix Table 22: Minimum probability of hospitalisation for typhoid fever for which vaccination is optimal for a range of willingness-to-pay (WTP) values. Threshold values are only shown for countries and willingness-to-pay values for which the uncertainty around typhoid incidence influences the optimal intervention strategy (either no vaccination 'No' or routine vaccination + catch-up campaign up to age 15 years 'RC15'). For countries and WTP values for which no values are shown, the optimal strategy is the same for each value of the typhoid hospitalisation rate considered for that country in our analysis. 61
Appendix Table 23: Minimum typhoid incidence rate for which vaccination is optimal for a range of willingness-to-pay (WTP) values. Threshold values are only shown for countries and willingness-to-pay values for which the uncertainty around typhoid incidence (cases per 100,000 person-years) influences the optimal intervention strategy (either no vaccination 'No' or routine vaccination + catch-up campaign up to age 15 years 'RC15'). For countries and WTP values for which no values are shown, the optimal strategy is the same for each value of typhoid incidence considered for that country in our analysis. 63
Appendix Table 24: Minimum case fatality rate among hospitalised patients (CFR_{hosp}) for which vaccination is optimal for a range of willingness-to-pay (WTP) values. Threshold values are only shown for countries and willingness-to-pay values for which the uncertainty around CFR_{hosp} influences the optimal intervention strategy (either no vaccination 'No' or routine vaccination + catch-up campaign up to age 15 years 'RC15'). For countries and WTP values for which no values are shown, the optimal strategy is the same for each value of typhoid CFR_{hosp} considered for that country in our analysis. 65
Appendix Table 25: Cost-effectiveness results when including Gavi's share for each country for a range of willingness-to-pay values (US$0-US$2000 per DALY averted). The optimal strategy (i.e. strategy that yields the highest *average* net monetary benefit) is indicated by the colour-shading: no vaccination (white), routine childhood immunisation (green), routine immunisation with a catch-up campaign up to age 5 years (purple) and routine immunisation with a catch-up campaign up to age 15 years (orange). How certain we are about the optimal strategy is indicated by the percentage of parameter samples in which the optimal strategy yielded the highest net benefit. 68
Appendix Table 26: Cost-effectiveness results when including Gavi's share for each country for a range of willingness-to-pay values (0 up to 4 times a country's GDP per capita). The optimal strategy (i.e. strategy that yields the highest *average* net monetary benefit) is indicated by the colour-shading: no vaccination (white),

routine childhood immunisation (green), routine immunisation with a catch-up campaign up to age 5 years (purple) and routine immunisation with a catch-up campaign up to age 15 years (orange). How certain we are about the optimal strategy is indicated by the percentage of parameter samples in which the optimal strategy yielded the highest net benefit. 70
Appendix Table 27: Cost-effectiveness results when including Gavi's share and assuming the delivery cost of campaign doses is the same as routine doses, for each country for a range of willingness-to-pay values (US$0-US$2000 per DALY averted). The optimal strategy (i.e. strategy that yields the highest *average* net monetary benefit) is indicated by the colour-shading: no vaccination (white), routine childhood immunisation (green), routine immunisation with a catch-up campaign up to age 5 years (purple) and routine immunisation with a catch-up campaign up to age 15 years (orange). How certain we are about the optimal strategy is indicated by the percentage of parameter samples in which the optimal strategy yielded the highest net benefit. 72
Appendix Table 28: Cost-effectiveness results when including Gavi's share and assuming the delivery cost of campaign doses is the same as routine doses, for each country for a range of willingness-to-pay values (0 up to 4 times a country's GDP per capita). The optimal strategy (i.e. strategy that yields the highest *average* net monetary benefit) is indicated by the colour-shading: no vaccination (white), routine childhood immunisation (green), routine immunisation with a catch-up campaign up to age 5 years (purple) and routine immunisation with a catch-up campaign up to age 15 years (orange). How certain we are about the optimal strategy is indicated by the percentage of parameter samples in which the optimal strategy yielded the highest net benefit. 74
Appendix Table 29: Cost-effectiveness results when assuming decreasing typhoid transmission over time, for each country for a range of willingness-to-pay values (US$0-US$2000 per DALY averted). The optimal strategy (i.e. strategy that yields the highest *average* net monetary benefit) is indicated by the colour-shading: no vaccination (white) and routine immunisation with a catch-up campaign up to age 15 years (orange). How certain we are about the optimal strategy is indicated by the percentage of parameter samples in which the optimal strategy yielded the highest net benefit. 76
Appendix Table 30: Cost-effectiveness results when assuming decreasing typhoid transmission over time, for each country for a range of willingness-to-pay values (0 up to 4 times a country's GDP per capita). The optimal strategy (i.e. strategy that yields the highest *average* net monetary benefit) is indicated by the colour-shading: no vaccination (white) and routine immunisation with a catch-up campaign up to age 15 years (orange). How certain we are about the optimal strategy is indicated by the percentage of parameter samples in which the optimal strategy yielded the highest net benefit. 78
Appendix Table 31:Cost-effectiveness results when including costs and health benefits for 30 years, for a range of willingness-to-pay values (US$0-US$2000 per DALY averted). The optimal strategy (i.e. strategy that yields the highest *average* net monetary benefit) is indicated by the colour-shading: no vaccination (white) and routine immunisation with a catch-up campaign up to age 15 years (orange). How certain we are about the optimal strategy is indicated by the percentage of parameter samples in which the optimal strategy yielded the highest net benefit. Results are only shown for the countries for which we could derive the Gavi contribution for 2029-2048, i.e. the countries assumed to not receive any additional support for 2029-2048. 80
Appendix Table 32: Cost-effectiveness results when including costs and health benefits for 30 years, for a range of willingness-to-pay values (0 up to 4 times a country's GDP per capita). The optimal strategy (i.e. strategy that yields the highest *average*

net monetary benefit) is indicated by the colour-shading: no vaccination (white) and routine immunisation with a catch-up campaign up to age 15 years (orange). How certain we are about the optimal strategy is indicated by the percentage of parameter samples in which the optimal strategy yielded the highest net benefit. Results are only shown for the countries for which we could derive the Gavi contribution for 2029-2048, i.e. the countries assumed to not receive any additional support for 2029-2048. 81

1.2 List of Figures

Appendix Figure 1: Compartmental diagram of dynamic model of typhoid transmission and vaccination. Black boxes correspond to the infection process in the absence of vaccination and red boxes correspond to vaccinated states. Orange lines depict the infection process, blue lines depict the recovery process, green lines depict the process by which individuals become chronic carriers, purple lines depict waning immunity (either natural or vaccine-derived immunity), and red lines depict the vaccination process. Red dashed lines represent vaccination of individuals who do not benefit from the vaccine; we assume the response to vaccination is "all-or-nothing" with probability *v*. Model parameters describe the time-varying rates at which the transitions between states occur. For clarity, the diagram does not depict the underlying age structure. 11

Appendix Figure 2: Comparison of estimates of typhoid incidence and mean age of typhoid fever cases from two models. The estimated typhoid fever incidence (per 100,000 person-years) for each country is plotted in the left panel, while the mean age of infection is plotted in the right panel for the "Yale model"[10] (black) and "IHME model"[14] (red). The dots represent the median estimate, while the lines represent the 95% credible intervals. The mean age of infection was calculated by multiplying the midpoint of each age group by the number of cases in that age group (equal to the age-specific incidence times the size of the population in that age group), then dividing by the total number of cases in the population. For our analysis, we sampled from the range of values between the means of the two models........ 15

Appendix Figure 3: Cost-effectiveness acceptability frontier for India, showing for a range of willingness-to-pay values (X-axis), the optimal strategy (colour) and how certain we are about the optimal strategy (Y-axis). The vertical black line shows the GDP per capita for India. 43

Appendix Figure 4: Relationship between typhoid fever incidence and the minimum willingness-to-pay value at which any vaccination strategy becomes optimal when compared to no vaccination. The annual incidence of symptomatic cases per 100,000 person-years (mean and 95% credible interval) used as an input in our health economic evaluation is plotted on the X-axis for 51 Gavi-eligible countries. The minimum WTP value at which vaccination becomes optimal when compared to no vaccination is plotted on the Y-axis for 51 Gavi-eligible countries. Lesotho, Kyrgyz Republic and Tajikistan are not shown on this plot as the minimum WTP value at which vaccination becomes optimal in these countries exceeds USD1200 per DALY averted. 57

Appendix Figure 5: Expected value of partial perfect information for three representative countries. The expected value of partial perfect information (EVPPI) for each uncertain parameter is plotted on the Y-axis for a range of willingness-to-pay values (X-axis) for Zimbabwe, Guinea Bissau and Myanmar. Vertical black bars represent the country's GDP per capita. Results are based on 2,000 parameter samples. Costs are presented in US$2016. We assume a vaccine price of US$1·50 per dose, but exclude Gavi's contribution depending on each country's expected graduation from Gavi support. See also https://ceatyphoid.uantwerpen.be/home/. 58

Appendix Figure 6: Expected value of partial perfect information for India and Pakistan. The expected value of partial perfect information (EVPPI) for each uncertain parameter is plotted on the Y-axis for a range of willingness-to-pay values (X-axis). See Appendix Figure 5 for legend of the coloured lines. Vertical black bars represent the country's GDP per capita. Results are based on 2,000 parameter samples. Costs are presented in US$2016. We assume a vaccine price of US$1·50

per dose, but exclude Gavi's contribution depending on each country's expected graduation from Gavi support. See also https://ceatyphoid.uantwerpen.be/home/. 59

2 Methods

2.1 Transmission-dynamic model

2.1.1 Description of the dynamic model

The compartmental model is illustrated in Appendix Figure 1. We assume individuals are born into the susceptible compartment (***S***) of the lowest age-stratum at a rate μN, where N is the total population size. In the absence of immunizations, the susceptible population is infected and enters the $\boldsymbol{I_M}$ compartment (infectious, with a possibility of symptoms) at a rate $\boldsymbol{\lambda}$ (also known as the force of infection). Following primary infection (average duration equal to $1/\delta$), some infectious individuals become life-long chronic carriers (and enter compartment ***C***) with an age-specific probability $\boldsymbol{\theta_a}$. Most cases, however, recover and gain temporary immunity to reinfection (compartment ***R***). Individuals lose immunity at a rate ω and enter compartment $\boldsymbol{S_R}$, and they are again susceptible to subclinical ($\boldsymbol{I_Q}$) infection occurring at a rate $\boldsymbol{\lambda}$. We assume that waning of immunity to symptomatic reinfection is zero, since previous efforts to estimate the rate of waning immunity resulted in estimates that were not significantly different from 0.[1] Again, individuals who experience re-infection are all assumed to recover and enter the ***R*** compartment, where they are temporarily immune to reinfection. The ordinary differential equations (ODEs) describing the model are as follows (for simplicity, we have excluded the underlying age structure):

Without vaccination

$$\frac{dS}{dt} = bN - \lambda S$$

$$\frac{dI_M}{dt} = \lambda S - \delta I_M$$

$$\frac{dR}{dt} = \delta(1 - \theta - \alpha)I_M + \delta I_Q - \omega R$$

$$\frac{dC}{dt} = \delta\theta I_M$$

$$\frac{dS_R}{dt} = \omega R - \lambda S_R$$

$$\frac{dI_Q}{dt} = \lambda S_R - \delta I_Q$$

With vaccination

$$\frac{dS}{dt} = bN - \lambda S - \kappa\nu S + \omega V_1$$

$$\frac{dI_M}{dt} = \lambda S - \delta I_M$$

$$\frac{dR}{dt} = \delta(1 - \theta - \alpha)I_M + \delta I_Q - \omega R - \kappa\nu R$$

$$\frac{dC}{dt} = \delta\theta I_M$$

$$\frac{dS_R}{dt} = \omega R - \lambda S_R - \kappa\nu S_R$$

$$\frac{dI_Q}{dt} = \lambda S_R - \delta I_Q$$

$$\frac{dV_1}{dt} = \kappa\nu S - \omega_V V_1$$

$$\frac{dV_2}{dt} = \kappa\nu(S_R + R) - \omega_V V_2$$

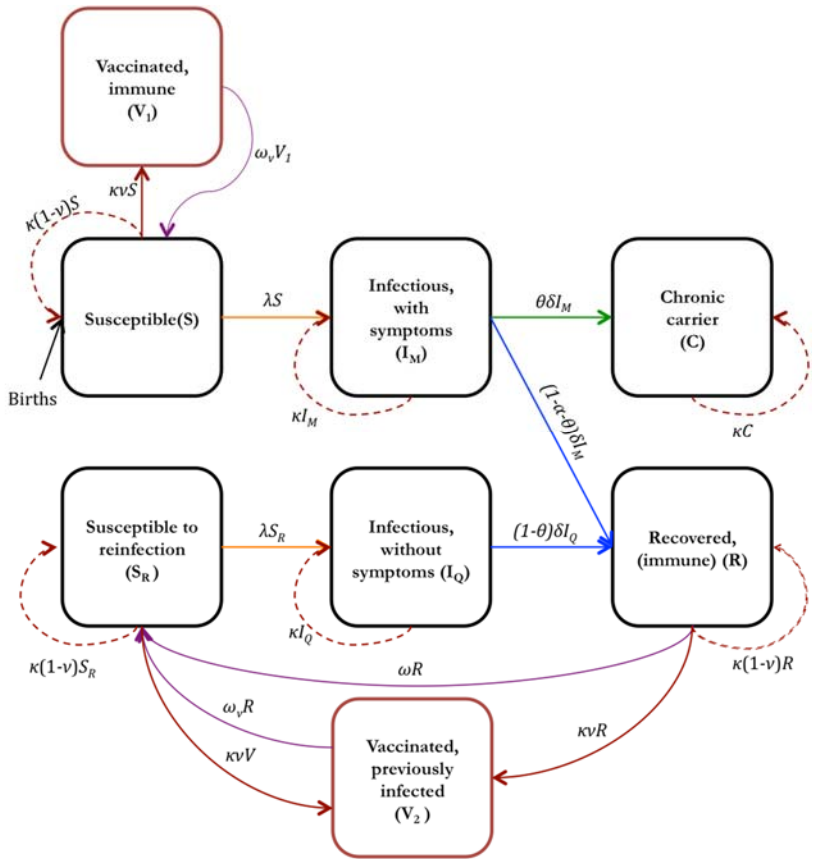

Appendix Figure 1: Compartmental diagram of dynamic model of typhoid transmission and vaccination. Black boxes correspond to the infection process in the absence of vaccination and red boxes correspond to vaccinated states. Orange lines depict the infection process, blue lines depict the recovery process, green lines depict the process by which individuals become chronic carriers, purple lines depict waning immunity (either natural or vaccine-derived immunity), and red lines depict the vaccination process. Red dashed lines represent vaccination of individuals who do not benefit from the vaccine; we assume the response to vaccination is "all-or-nothing" with probability *v*. Model parameters describe the time-varying rates at which the transitions between states occur. For clarity, the diagram does not depict the underlying age structure.

The force of infection varies by age ($\boldsymbol{\lambda_a}$) and is a function of symptomatic and subclinical infectious individuals ($\boldsymbol{I_M}$ and $\boldsymbol{I_Q}$) and chronic carriers ($\boldsymbol{C}$). We assume chronic carriers contribute to the force of infection at a fraction $\boldsymbol{r}$ of the rate of acute infectious individuals, but remain infectious for the rest of their lives. We assume frequency-dependent transmission, such that the force of infection is the product of the age-specific transmission rate, $\boldsymbol{\beta_a}$, and the weighted sum of individuals in compartments $\boldsymbol{I_M}$, $\boldsymbol{I_Q}$, and $\boldsymbol{C}$ divided by the total population size $\boldsymbol{N}$:

$$\boldsymbol{\lambda_a = \frac{\beta_a}{N} \sum_{\text{All ages}} (I_M + I_Q + rC)}$$

We allow for a lower transmission rate among individuals in the two youngest age groups (infants 0-<2 years of age and children 2-<5 years of age), as this has been shown to provide the best fit to the available age-specific incidence data.[2, 3] The rate of transmission was equal to $m_1\beta_0$ for 0-<2 year olds and $m_2\beta_0$ for 2-<5 year olds, and β_0 for all other age groups.

We do not distinguish between short- versus long-cycle transmission (the latter occurring at a slight delay via broader environmental contamination of food and water sources) because these two transmission routes are not individually identifiable from

the annual incidence estimates used for model fitting. Furthermore, *S.* Typhi bacteria are relatively short-lived in the environment (on the order of weeks to months).[4] Therefore, we assumed all transmission is directly proportional to the number of currently infectious individuals. This assumption is unlikely to affect our conclusions regarding the impact of vaccination, since as with previous models, we assume the source of exposure does not affect the probability of infection.

2.1.2 Basic reproduction number (R_0)

The basic reproduction number (R_0) is defined as the expected number of secondary infections produced by an infectious individual in a fully susceptible population. It provides a summary of the relationship between the transmission rate, demographic characteristics, the natural history of infection, and disease dynamics. This measure will serve us to transform estimated incidence and average age of infection into dynamic model parameters that will reproduce the incidence in each country. In our dynamic model of transmission, R_0 is given by the equation:

$$R_0 = \frac{\bar{\beta}}{\delta + \mu}\left(1 + \frac{r\delta\bar{\theta}}{\mu}\right)$$

where $\bar{\beta}$ is the overall transmission parameter and $\bar{\theta}$ is the average probability of becoming a chronic carrier, and μ is the average death rate over time (equal to the average crude birth rate, since we assume a constant population size); other parameters are described above and in Table 1 of the main text. The values of $\bar{\beta}$ and $\bar{\theta}$ used to calculate R_0 are equal to the sum of the respective age-specific parameters weighted by the proportion of the population in each age group.

We estimated R_0 for each country based on the annual incidence rate and average age of infection. A transmission rate that results in R_0<1 would result in a simulation in which the disease would eventually go extinct, so back-calculating the transmission rate from R_0 values greater than 1 ensured that we would select transmission parameter values that were consistent with sustained transmission in the absence of vaccination.

2.1.3 Vaccination

In order to simulate the outcome of vaccine interventions, two additional compartments were added to the model: ***V_1*** denotes persons who are immunologically naïve to typhoid fever (i.e. never infected) and successfully mount a protective immunological response to vaccination. ***V_2*** denotes successfully immunized individuals who are already partially immune to clinical infection; we assume vaccination protects them from subclinical infection and precludes them from contributing to transmission. We assume the duration of vaccine protection follows an exponential distribution with rate parameter for immunity loss equal to the inverse of the **mean** duration of vaccine-induced immunity. This characterization of the duration of protection is typical of differential-equation-based models and is more consistent with observed antibody responses than assuming a fixed duration of immunity.[5-7]

2.1.4 Estimating country-specific parameters for the dynamic model

2.1.4.1 Background demography: birth rate and population age distribution

In order to find transmission rate values that reproduce the incidence and average age of typhoid infection in each country, it is necessary to condition the parameter

estimation process on the demographic profile of the population in each country, since the transmission rate in a population and the concomitant disease dynamics (characterized by the age-specific incidence and the mean age of infection) are intimately related to the demographic profile of the population (as parameterized by the birth rate and the underlying age distribution of a population).[1] Because simulating incidence while accounting for country-specific birth rates and age-distributions would represent an unfeasible computational effort, we simplified our task by grouping countries into three 'standard' demographic profiles. We selected the 'standard' demographic profiles according to estimates for all low-income countries, lower-middle-income countries, and upper- middle-income countries as listed in the UN World Population Prospects (WPP) database.[8] We refer to these sets of estimates as very young, young, and average populations, respectively. We assigned each country to a standard demographic profile according to the proportion of the population under 5 that most closely matched the population <5 year of age in each standard demographic profile: "very young" (16%), "young" (10%), and "average" (7%). The full age distribution of each demographic profile as provided by the WPP is listed in Appendix Table 1. Appendix Table 2 shows the data from Appendix Table 1, but adjusted to the age groups that would be necessary for our simulations.

Appendix Table 1: Age distribution of three demographic profiles according to the UN World Population Prospects database.

Age group (years)	Very young population	Young population	Average population
**0-<5**	0.16	0.11	0.07
**5-<10**	0.14	0.10	0.07
**10-<15**	0.12	0.10	0.07
**15-<20**	0.11	0.09	0.07
**20-<25**	0.09	0.09	0.08
**≥25**	0.38	0.51	0.64

Appendix Table 2: Age distribution of three demographic profiles according to the UN World Population Prospects database, adjusted for the age groups from our model.

	Very young population	Young population	Average population
**Birth rates (per week)**	6.9×10^{-4}	4.5×10^{-4}	2.9×10^{-4}
**Proportion of population in each age group***			
**Ages 0-≤9 mos**	0.0318	0.0216	0.0144
**Ages 9 mos-<2 yrs**	0.0318	0.0216	0.0144
**Ages 2 yrs - <5 yrs**	0.0953	0.0648	0.0433
**Ages 5-15 yrs**	0.2660	0.2022	0.1358
**Ages 15–25 yrs**	0.2002	0.1848	0.1484
**Ages ≥25 yrs**	0.3749	0.5050	0.6436
**Death rates by age group (per week)****			
**Ages 0-9 mos**	-0.0039	-0.0049	-0.0058
**Ages 9 mos-<2 yrs**	0.0103	0.0103	0.0103
**Ages 2 yrs - <5 yrs**	-0.0013	-0.0013	-0.0013
**Ages 5-15 yrs**	0.0004	0.0001	0.0001
**Ages 15–25 yrs**	0.0006	0.0002	-0.0002
**Ages ≥25 yrs**	0.0010	0.0007	0.0004

*Because child mortality is higher in 0-9m than in 9 mos-2 yrs, we assigned half of children <2 to the 0-9m category and the other half to the larger, but older, category of 9 mos - <2 yrs.

**Calculated to keep the size of the age group constant in light of the number of births and the number of people aging in and out of each age group.

2.1.4.2 Disease transmission parameters

Our model was previously fit by maximum likelihood to a 12-year time series of age-specific hospitalization data from Vellore, India, and subsequently fit to age-specific incidence data from five more settings to conduct an in-depth study of disease dynamics and cost-effectiveness of vaccination with typhoid Vi-conjugate vaccine (TCV).[1, 2] The parameter estimates from the five low- and middle-income settings provided us with distributions for the relative risk of transmission for children <5 years of age (m_1 and m_2) and for the relative contribution of chronic carriers to the force of infection (r) (see Table 1 for specifications). All other model parameters were derived from the literature (see Table 1 in the main text) with the exception of the country-specific demographic parameters (birth rate and age distribution), the overall transmission rate $(\bar{\beta})$, and the reporting probability (the probability that a case will be symptomatic, s), which we estimated for each of the 54 Gavi-eligible countries as detailed below. Table 1 in main text shows the parameter values in the model.

2.1.4.2.1 Overall typhoid incidence

Relatively few studies have been conducted to measure the population-based incidence of typhoid fever. Existing estimates of typhoid burden are based on data from only 22 sites in 14 countries.[9] Thus, the incidence of typhoid fever in most countries is highly uncertain, and model-based estimates of typhoid incidence have exhibited considerable country-to-country differences.[10-13] We considered the predicted burden of disease from two sources: a previously published and validated model from Yale University[10] and the estimates published by the Institute of Health Metrics and Evaluation (IHME).[14] We performed a comparison of the country-by-country incidence estimates (Appendix Figure 2). We found that, in many countries, the variance between models was greater than the variance within models. We therefore refrained from selecting one model, as we had no basis to do so, and because selecting one model over the other poses various risks to our conclusions: we believe the IHME estimates underrepresent the size of the uncertainty in typhoid incidence (when compared to the Yale model, which accounts for uncertainty in the observation process), and the Yale and IHME models disagree on the average incidence in some countries, thus risking over- or under-stating the case for vaccination in those countries. Therefore, our approach detailed below takes into account the between-model variance of incidence and average of infection.

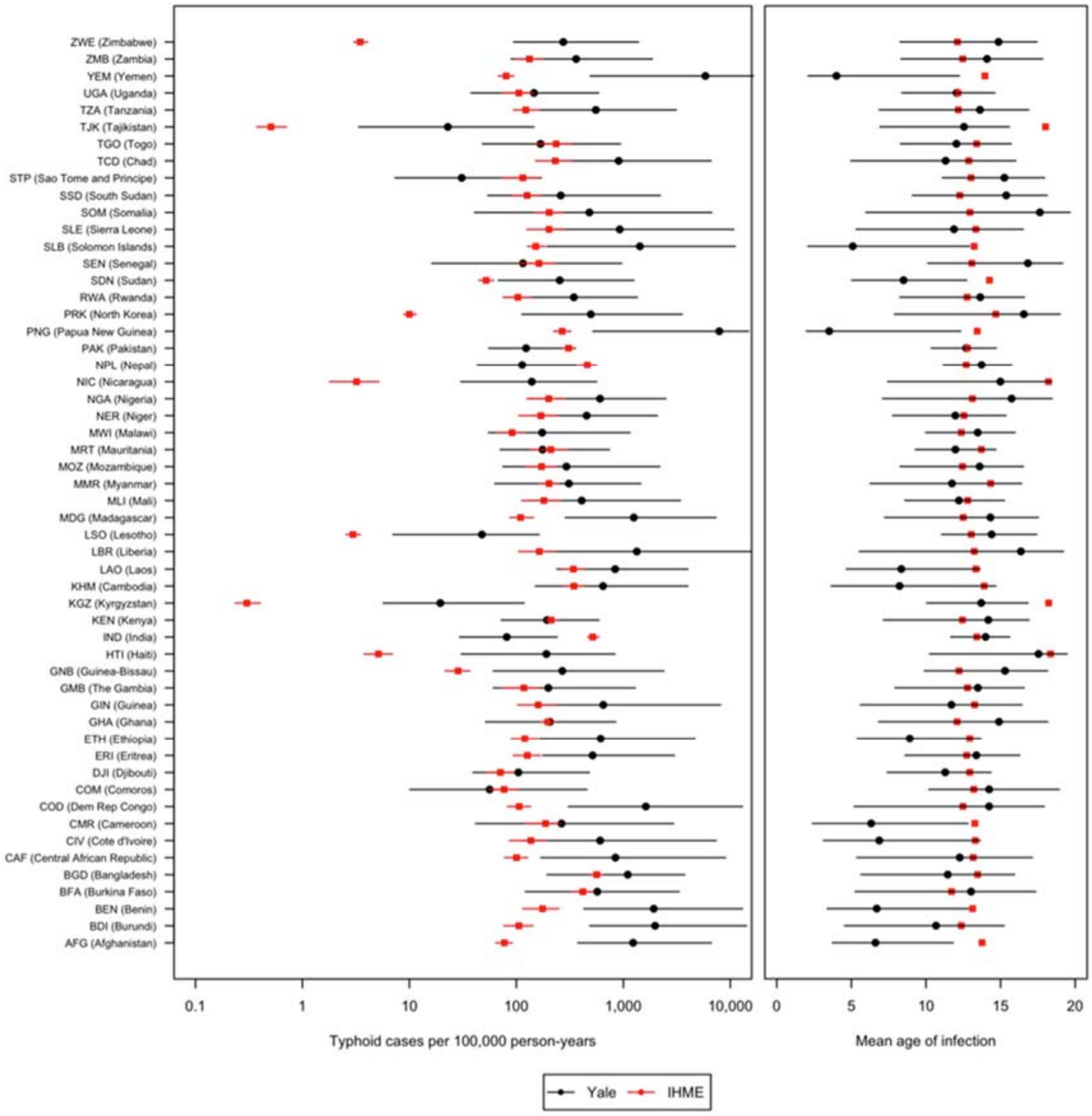

Appendix Figure 2: Comparison of estimates of typhoid incidence and mean age of typhoid fever cases from two models. The estimated typhoid fever incidence (per 100,000 person-years) for each country is plotted in the left panel, while the mean age of infection is plotted in the right panel for the "Yale model"[10] (black) and "IHME model"[14] (red). The dots represent the median estimate, while the lines represent the 95% credible intervals. The mean age of infection was calculated by multiplying the midpoint of each age group by the number of cases in that age group (equal to the age-specific incidence times the size of the population in that age group), then dividing by the total number of cases in the population. For our analysis, we sampled from the range of values between the means of the two models.

2.1.4.2.2 Age-specific transmission rate, and reporting probability.

Estimating transmission parameters via traditional fitting algorithms (e.g. Nelder-Mead, quasi-Newton methods) or sampling algorithms (e.g. MCMC, Hamiltonian Monte Carlo) is computationally prohibitive for an analysis of 54 countries. Instead, we simulated incidence and the average age of infection for each standard demographic profile from a set of pre-selected values of R_0 (and the corresponding transmission rate conditional on the birth rate for each demographic profile). We then approximated the relationship between transmission, incidence, and the average age of infection for all values of R_0 by constructing cubic interpolators based on the simulated incidence and average age of infection from the dynamic model at the pre-determined range of R_0 values.

We then used the average age of infection from the Yale and IHME burden models to estimate the R_0 for each country (along with the concomitant uncertainty). By extension, we decomposed the uncertainty in R_0 into the uncertainty in the transmission rate, the transmission rate among children <5 years of age (m_1, m_2), and the relative infectiousness of chronic carriers (r). Below is a detailed outline of the algorithm we employed:

(1) Using the system of ODEs that describes the compartmental model of typhoid transmission, we used a numeric solver (based on a Runge-Kutta algorithm) to simulate incidence and average age of infection across a range of R_0 values for each of the three standard demographic profiles (extremely young, very young, and young populations).
(2) We used cubic interpolators to approximate the relationship between R_0 and the average age of infection (interpolator #1) and R_0 and disease incidence (interpolator #2).
(3) Based on interpolator #1, we identified the value of R_0 that most closely reproduced the average age of infection (Appendix Figure 2, right panel) given the country's standard demographic profile.
(4) Based on interpolator #2, we calculated the "reporting rate" (or rate of symptomatic disease, s) by comparing the model-simulated incidence at a transmission level calculated in (3) to the estimated incidence from the burden models (Appendix Figure 2, left panel), such that the reporting rate = (overall incidence per year according to the burden estimate)/(ODE model-predicted incidence of infection per year|R_0).
(5) To account for the uncertainty in R_0 that arises from the difference in the average age of infection according to the Yale and IHME burden models, and to integrate the uncertainty in select disease parameters (m_1, m_2, r), we devised the following algorithm. For each iteration i in 1:2000, and for each country:
    a) Take a sample z(i) from a uniform distribution bound by (0,1).
    b) Draw a new value for R_0 and for incidence of symptomatic disease such that the new $R_0(i)$ and incidence is between the estimates from both burden models (based on the average age of infection):
    $$R_0(i) = \min(R_{0,\text{Yale}}, R_{0,\text{IHME}}) + z(i)*\text{abs}(R_{0,\text{Yale}} - R_{0,\text{IHME}})$$
    $$\text{Incidence of symptomatic infection}(i) = \min(Incidence_{\text{Yale}}, Incidence_{\text{IHME}}) + z(i)*\text{abs}(Incidence_{\text{Yale}} - Incidence_{\text{IHME}})$$
    c) Using interpolator #2, calculate the new hypothetically observed incidence at $R_0(i)$ and, by extension, the new reporting rate, s:
    $$s(i) = \frac{\text{Incidence of symptomatic infection}(i)}{\text{ODE model-predicted incidence}|R_0(i)}$$
    d) Back-calculate $\beta_0(i)$ given $R_0(i)$ and random samples from the distributions for m_1, m_2, and r.

2.1.5 Simulating typhoid incidence in the context of vaccination

2.1.5.1 Vaccine coverage

Vaccine coverage was estimated by Gavi based on demand forecasting for introduction between 2019 and 2030 under the assumptions of "constrained" supplies of typhoid Vi-conjugate vaccine (TCV) and "unconstrained" (or unlimited) supplies of TCV. The forecast assuming constrained supplies assumes that some countries will introduce vaccination at a later date or at a lower coverage than the forecast assuming unconstrained supplies. The coverage estimates under the constrained and

unconstrained scenarios were identical except for nine countries (Afghanistan, Bangladesh, India, Malawi, Myanmar, Pakistan, Rwanda, Senegal, and Zimbabwe). Introduction is expected to occur in different years in four countries (Myanmar, Rwanda, Senegal, and Zimbabwe), but uptake trends are expected to be the same. However, in the other five countries (Afghanistan, Bangladesh, India, Malawi, Pakistan) coverage trends differed between the two forecasts. For our analysis, we assumed vaccine uptake would follow the unconstrained scenario (Appendix Table 3 and Appendix Table 4), as we wanted to model vaccine uptake in each country independent of the other countries. Nevertheless, we verified that our results were not sensitive to this assumption, as the differences in the forecasts are minimal.

Although Gavi forecasts that countries will introduce TCVs in different years, this information is uncertain and considered confidential. We assumed that all countries would introduce TCV at the same time (i.e. in 2019), and evaluated cost-effectiveness per country over the first ten years following introduction. We believe that this approach is sensible because decision-makers consider questions of cost-effectiveness in their own country independent of implementation in other countries.

Appendix Table 3: Assumed country-specific vaccine coverage over time for routine immunisation when assuming unconstrained supplies. Coverage estimates for the years 11 up to 30 are available from the authors.

country	Year_01	Year_02	Year_03	Year_04	Year_05	Year_06	Year_07	Year_08	Year_09	Year_10
**Afghanistan**	0.68	0.69	0.7	0.71	0.72	0.73	0.74	0.75	0.76	0.76
**Bangladesh**	0.95	0.95	0.95	0.95	0.95	0.95	0.95	0.95	0.95	0.95
**Benin**	0.85	0.86	0.87	0.88	0.88	0.88	0.88	0.88	0.88	0.88
**Burkina Faso**	0.95	0.95	0.95	0.95	0.95	0.95	0.95	0.95	0.95	0.95
**Burundi**	0.95	0.95	0.95	0.95	0.95	0.95	0.95	0.95	0.95	0.95
**Cambodia**	0.88	0.89	0.9	0.91	0.92	0.93	0.94	0.95	0.95	0.95
**Cameroon**	0.89	0.9	0.91	0.92	0.92	0.92	0.92	0.92	0.92	0.92
**Central African Republic**	0.6	0.61	0.62	0.63	0.63	0.63	0.63	0.63	0.63	0.63
**Chad**	0.7	0.71	0.72	0.72	0.72	0.72	0.72	0.72	0.72	0.72
**Comoros**	0.99	0.99	0.99	0.99	0.99	0.99	0.99	0.99	0.99	0.99
**Cote dIvoire**	0.91	0.91	0.91	0.91	0.91	0.91	0.91	0.91	0.91	0.91
**Dem Republic of Congo**	0.44	0.89	0.9	0.91	0.91	0.91	0.91	0.91	0.91	0.91
**Djibouti**	0.85	0.86	0.87	0.88	0.89	0.89	0.89	0.89	0.89	0.89
**DPR Korea**	0.98	0.98	0.98	0.98	0.98	0.98	0.98	0.98	0.98	0.98
**Eritrea**	0.95	0.95	0.95	0.95	0.95	0.95	0.95	0.95	0.95	0.95
**Ethiopia**	0.4	0.8	0.81	0.82	0.83	0.84	0.84	0.84	0.84	0.84
**Gambia**	0.97	0.97	0.97	0.97	0.97	0.97	0.97	0.97	0.97	0.97
**Ghana**	0.95	0.95	0.95	0.95	0.95	0.95	0.95	0.95	0.95	0.95
**Guinea**	0.68	0.68	0.68	0.68	0.68	0.68	0.68	0.68	0.68	0.68
**Guinea Bissau**	0.9	0.91	0.92	0.93	0.94	0.95	0.95	0.95	0.95	0.95
**Haiti**	0.65	0.66	0.67	0.67	0.67	0.67	0.67	0.67	0.67	0.67
**India**	0.26	0.56	0.8	0.95	0.95	0.95	0.95	0.95	0.95	0.95
**Kenya**	0.82	0.83	0.84	0.85	0.86	0.87	0.88	0.89	0.89	0.89
**Kyrgyz Republic**	0.97	0.97	0.97	0.97	0.97	0.97	0.97	0.97	0.97	0.97
**Lao PDR**	0.82	0.83	0.84	0.85	0.86	0.87	0.88	0.89	0.9	0.9
**Lesotho**	0.95	0.95	0.95	0.95	0.95	0.95	0.95	0.95	0.95	0.95
**Liberia**	0.89	0.9	0.91	0.92	0.93	0.94	0.94	0.94	0.94	0.94

**Madagascar**	0.68	0.69	0.7	0.71	0.72	0.72	0.72	0.72	0.72	0.72
**Malawi**	0.85	0.86	0.87	0.88	0.89	0.9	0.91	0.92	0.93	0.94
**Mali**	0.88	0.89	0.89	0.89	0.89	0.89	0.89	0.89	0.89	0.89
**Mauritania**	0.79	0.8	0.81	0.82	0.83	0.84	0.84	0.84	0.84	0.84
**Mozambique**	0.95	0.95	0.95	0.95	0.95	0.95	0.95	0.95	0.95	0.95
**Myanmar**	0.95	0.95	0.95	0.95	0.95	0.95	0.95	0.95	0.95	0.95
**Nepal**	0.86	0.87	0.88	0.89	0.9	0.91	0.92	0.93	0.94	0.95
**Nicaragua**	0.99	0.99	0.99	0.99	0.99	0.99	0.99	0.99	0.99	0.99
**Niger**	0.84	0.85	0.86	0.87	0.88	0.88	0.88	0.88	0.88	0.88
**Nigeria**	0.34	0.61	0.62	0.63	0.64	0.65	0.65	0.65	0.65	0.65
**Pakistan**	0.44	0.67	0.68	0.69	0.7	0.71	0.72	0.73	0.74	0.75
**Papua New Guinea**	0.77	0.78	0.79	0.8	0.81	0.82	0.83	0.84	0.84	0.84
**Republic of Sudan**	0.94	0.95	0.95	0.95	0.95	0.95	0.95	0.95	0.95	0.95
**Rwanda**	0.95	0.95	0.95	0.95	0.95	0.95	0.95	0.95	0.95	0.95
**Sao Tome e Principe**	0.95	0.95	0.95	0.95	0.95	0.95	0.95	0.95	0.95	0.95
**Senegal**	0.95	0.95	0.95	0.95	0.95	0.95	0.95	0.95	0.95	0.95
**Sierra Leone**	0.95	0.95	0.95	0.95	0.95	0.95	0.95	0.95	0.95	0.95
**Solomon Islands**	0.99	0.99	0.99	0.99	0.99	0.99	0.99	0.99	0.99	0.99
**Somalia**	0.59	0.6	0.6	0.6	0.6	0.6	0.6	0.6	0.6	0.6
**South Sudan**	0.3	0.31	0.32	0.33	0.34	0.34	0.34	0.34	0.34	0.34
**Tajikistan**	0.97	0.97	0.97	0.97	0.97	0.97	0.97	0.97	0.97	0.97
**Tanzania**	0.95	0.95	0.95	0.95	0.95	0.95	0.95	0.95	0.95	0.95
**Togo**	0.95	0.95	0.95	0.95	0.95	0.95	0.95	0.95	0.95	0.95
**Uganda**	0.94	0.95	0.95	0.95	0.95	0.95	0.95	0.95	0.95	0.95
**Yemen**	0.8	0.81	0.82	0.83	0.84	0.84	0.84	0.84	0.84	0.84
**Zambia**	0.95	0.95	0.95	0.95	0.95	0.95	0.95	0.95	0.95	0.95
**Zimbabwe**	0.95	0.95	0.95	0.95	0.95	0.95	0.95	0.95	0.95	0.95

Appendix Table 4: Assumed country-specific vaccine coverage over time for campaign when assuming unconstrained supplies.

country	Year_01	Year_02	Year_03	Year_04
**Afghanistan**	0.9	0	0	0
**Bangladesh**	0.9	0	0	0
**Benin**	0.9	0	0	0
**Burkina Faso**	0.9	0	0	0
**Burundi**	0.9	0	0	0
**Cambodia**	0.9	0	0	0
**Cameroon**	0.9	0	0	0
**Central African Republic**	0.9	0	0	0
**Chad**	0.9	0	0	0
**Comoros**	0.9	0	0	0
**Cote dIvoire**	0.9	0	0	0
**Dem Republic of Congo**	0.45	0.44	0	0
**Djibouti**	0.9	0	0	0
**DPR Korea**	0.9	0	0	0
**Eritrea**	0.9	0	0	0
**Ethiopia**	0.45	0.45	0	0
**Gambia**	0.9	0	0	0
**Ghana**	0.9	0	0	0
**Guinea**	0.9	0	0	0
**Guinea Bissau**	0.9	0	0	0
**Haiti**	0.9	0	0	0
**India**	0.22	0.22	0.22	0.22
**Kenya**	0.9	0	0	0
**Kyrgyz Republic**	0.9	0	0	0
**Lao PDR**	0.9	0	0	0
**Lesotho**	0.9	0	0	0
**Liberia**	0.9	0	0	0
**Madagascar**	0.9	0	0	0
**Malawi**	0.9	0	0	0
**Mali**	0.9	0	0	0
**Mauritania**	0.9	0	0	0
**Mozambique**	0.9	0	0	0
**Myanmar**	0.9	0	0	0
**Nepal**	0.9	0	0	0
**Nicaragua**	0.9	0	0	0
**Niger**	0.9	0	0	0
**Nigeria**	0.45	0.44	0	0
**Pakistan**	0.45	0.45	0	0
**Papua New Guinea**	0.9	0	0	0
**Republic of Sudan**	0.9	0	0	0
**Rwanda**	0.9	0	0	0
**Sao Tome e Principe**	0.9	0	0	0
**Senegal**	0.9	0	0	0
**Sierra Leone**	0.9	0	0	0
**Solomon Islands**	0.9	0	0	0
**Somalia**	0.9	0	0	0

**South Sudan**	0.9	0	0	0
**Tajikistan**	0.9	0	0	0
**Tanzania**	0.9	0	0	0
**Togo**	0.9	0	0	0
**Uganda**	0.9	0	0	0
**Yemen**	0.9	0	0	0
**Zambia**	0.9	0	0	0
**Zimbabwe**	0.9	0	0	0

2.1.5.2 Vaccine-related parameters

Appendix Table 5 shows the assumed vaccine characteristics (also describe in Table 1 of the main text).

Appendix Table 5: Vaccine-related parameters used in the transmission-dynamic model.

Characteristic	Value	Source
**Age groups vaccinated**<br>**routine**<br>**catch-up campaign**	 9 mo<br>9 mo to <5 yrs<br>9 mo to <15 yrs	Based on the WHO recommendation[15]
**Initial efficacy of TCV (ν)**	Uniform(0.80, 0.95)	Based on vaccine efficacy of Typbar-TCV against a clinically-relevant endpoint in a human challenge model, and seroefficacy of Typbar-TCV estimated from immunogenicity data[6, 16]
**Duration of vaccine-induced immunity ($1/\omega_V$) (years)**	Uniform(10, 20)	Upper bound is based on a re-analysis of Vi-rEPA data fitting exponential waning rate to vaccine effectiveness over 1-4 years of follow-up, and half the estimated duration as a lower bound.[1, 2]
**Vaccine coverage**<br>**routine (κ_r)**<br>**campaign (κ_c)**	Varies by country	Gavi demand forecasts under the assumption of unconstrained supply (Appendix Table 3 and Appendix Table 4)
**Number of doses**	1	WHO recommendation and immunogenicity data suggesting a robust immune response following 1 dose[15, 17]

The only currently available and WHO pre-qualified TCV is the Typbar-TCV (Bharat Biotech). This vaccine was licensed based on immunogenicity data and efficacy data from a controlled human challenge study of naïve volunteers.[6, 16, 17] Vaccine effectiveness trials are currently ongoing.[18] Thus, the only currently available effectiveness data from a field study for a TCV is for the Vi-rEPA vaccine. We used the data on vaccine effectiveness over 1-4 years of follow-up from this trial to inform our estimates of the upper-bound on the initial efficacy of TCV (ν) and the average duration of vaccine-induced immunity ($1/\omega_V$), as detailed in Antillón et al.[2] We based our lower-bound estimates of the initial efficacy of TCV on the estimated "seroefficacy" of Typbar-TCV[6] and the similar estimate of vaccine efficacy against a clinical-relevant definition of typhoid fever in the human challenge study.[16] For the lower bound on the duration of vaccine-induced immunity, we assumed a value of 10 years, equal to approximately half of our estimate of the average duration for the Vi-rEPA vaccine.

2.1.5.3 Simulation of typhoid incidence with and without vaccination

The population-level incidence of typhoid was simulated by running the dynamic model until it reached equilibrium (500-year burn-in period). Then, we simulated different vaccine intervention strategies and their impact on disease incidence over the 30 years following vaccine introduction. The models were run 2000 times for each country, each time sampling from the distributions for the estimated disease and vaccination parameters (Table 1 of the main text, Appendix 2.1.4.2.2 above). The simulation output included incidence by country, age group, year since vaccination, and parameter sample (2000 in total), as well as the corresponding number of routine and campaign vaccine in a population of constant size.

We also explored the sensitivity of our results for the cost-effectiveness of vaccination to an exogenous decrease in the typhoid transmission rate, e.g. due to ongoing improvements in water and sanitation. We assumed a linear decrease in the transmission rate of typhoid $(\bar{\beta})$ such that after 10 years, the transmission rate would be 75% of its estimated level at year 0.[19] Results of this sensitivity analysis are presented in section 3.3.3.

2.2 Input parameters for cost-effectiveness analysis

2.2.1 Output from transmission-dynamic model

The uncertainty around the estimated disease transmission parameters was propagated through the transmission-dynamic model, reflected in the uncertainty around the output of the dynamic model, i.e. the annual number of symptomatic typhoid infections by age and over time in the context of no intervention and for each of the three vaccination strategies (2000 samples each). The latter was used directly as an input in the economic model; hence, the uncertainty in the cost-effectiveness results incorporates the uncertainty of the parameters in the transmission-dynamic model.

It was not possible to consider the influence of uncertainty in the estimated transmission parameters separately due to the way the dynamic model was fit (see section 2.1.2). Instead, we considered the uncertainty of the two main variables used to fit the dynamic model, i.e. the mean annual number of symptomatic typhoid cases (Appendix Table 6) and the average age of typhoid cases (in the absence of vaccination). These two variables are more easily interpretable and can be directly measured in the field.

Appendix Table 6: Country-specific average annual typhoid incidence (cases per 100,000 personyears) in the absence of vaccination, used as input in the economic model. Median and 95% credible interval are shown.

	median	lower limit	upper limit
**Afghanistan**	706	103	1322
**Bangladesh**	875	592	1153
**Benin**	1131	229	2042
**Burkina Faso**	538	440	650
**Burundi**	1134	165	2120
**Cambodia**	519	368	681
**Cameroon**	239	194	289
**Central African Republic**	510	126	928
**Chad**	615	271	986
**Comoros**	74	59	92
**Cote dIvoire**	403	160	645

**Dem Republic of Congo**	913	154	1808
**Djibouti**	91	73	110
**DPR Korea**	261	20	504
**Eritrea**	353	151	572
**Ethiopia**	385	143	643
**Gambia**	174	126	229
**Ghana**	224	193	262
**Guinea**	435	184	705
**Guinea Bissau**	173	38	310
**Haiti**	111	11	229
**India**	315	98	531
**Kenya**	225	193	257
**Kyrgyz Republic**	11	1	21
**Lao PDR**	614	362	871
**Lesotho**	26	4	49
**Liberia**	867	220	1584
**Madagascar**	787	154	1405
**Malawi**	146	99	195
**Mali**	318	200	453
**Mauritania**	212	179	246
**Mozambique**	255	185	337
**Myanmar**	261	206	314
**Nepal**	297	128	472
**Nicaragua**	77	7	157
**Niger**	327	188	498
**Nigeria**	464	236	724
**Pakistan**	223	133	319
**Papua New Guinea**	1995	471	2188
**Republic of Sudan**	164	60	274
**Rwanda**	246	119	386
**Sao Tome e Principe**	84	37	135
**Senegal**	160	122	218
**Sierra Leone**	624	237	1013
**Solomon Islands**	842	194	1506
**Somalia**	399	234	639
**South Sudan**	215	138	304
**Tajikistan**	14	1	29
**Tanzania**	366	145	613
**Togo**	221	177	269
**Uganda**	136	111	164
**Yemen**	2735	224	3302
**Zambia**	266	152	410
**Zimbabwe**	150	10	311

2.2.2 Typhoid mortality

2.2.2.1 General

A systematic review and meta-analysis was performed to estimate the case fatality rate of typhoid fever.[20] Based on 44 outcomes reported in 40 studies, the case fatality rate for enteric fever in persons seeking healthcare was estimated to be 0·0249 with 95% prediction interval [0·0025-0·2086]. If we limit the analysis to Gavi-eligible countries, the estimate becomes 0·0267 with 95% prediction interval [0·0018-0·298], suggesting the case fatality rate does not differ significantly between Gavi-eligible and non-eligible countries. Therefore, we used all studies to include as much information as is available.

Twenty-one of the reported outcomes were from studies based on inpatients only, which resulted in a case fatality rate of 0·0445 with 95% prediction interval [0·0084-0·2038]. Two reported outcomes were based on studies that included outpatients only, with reported case fatality rates of 0·074 (Butler 1982) and 0·000 (Lin, 2000).[20] Eleven reported outcomes were based on studies that included both inpatients and outpatients, which resulted in a case fatality rate of 0·0056 with 95% prediction interval [0·0001-0·3024]; however, the proportion of inpatients versus outpatients was not reported in most of these studies. For the remaining ten reported outcomes, it was unclear if both inpatients and outpatients were included. The overall case fatality rate for typhoid fever based on three studies with active surveillance was 0·0028 with 95% prediction interval [0-0·8387].

2.2.2.2 Typhoid case fatality rate among inpatients

For our cost-effectiveness analysis, we used the case fatality rate based on the reported outcomes from inpatients only as input for the probability of death from typhoid fever among hospitalized cases. For eight countries (Bangladesh, Ethiopia, Ivory Coast, Lao, Zimbabwe, Nigeria, Senegal and India), country-specific estimates were available (Appendix Table 7):

Appendix Table 7: Country-specific case fatality rates for hospitalized patients with typhoid fever used in our cost-effectiveness analysis.

	**Value (median (mean) [95% CI])**	**Uncertainty distribution**	**Source/Assumptions**
Bangladesh	0.031 (0.063) [0.002-0.329]	$logit^{-1}$ (normal(mean=-3.45, se=0.72*2))	[20]; standard error (SE)*2 because for each study we are extrapolating from a single setting to country
Ethiopia	0.121 (0.150) [0.024-0.434]	$logit^{-1}$ (normal(mean=-1.99, se=0.44*2))	
Côte d'Ivoire	0.037 (0.046) [0.009-0.133]	$logit^{-1}$ (normal(mean=-3.24, se=0.36*2))	
Lao	0.014 (0.025) [0.002-0.118]	$logit^{-1}$ (normal(mean=-4.27, se=0.58*2))	
Zimbabwe	0.069 (0.076) [0.026-0.172]	$logit^{-1}$ (normal(mean=-2.59, se=0.25*2))	
India	0.057 (0.061) [0.027-0.117]	$logit^{-1}$ (normal(mean=-2.80, se=0.38))	[20]; common estimate and confidence interval random effects meta-analysis including 5/2/2 data points (India/Nigeria/Senegal)
Nigeria	0.157 (0.160) [0.096-0.242]	$logit^{-1}$ (normal(mean=-1.69, se=0.28))	
Senegal	0.032 (0.035) [0.012-0.079]	$logit^{-1}$ (normal(mean=-3.41, se=0.51))	

For all other countries, we use the common estimate of 0·0445 (see section 2.2.2.1). Uncertainty around the probability of death from typhoid among inpatients was characterized by the inverse logit of a normal distribution with mean equal to the

common estimate from the random-effects meta-analysis (-3·07) and standard error equal to the standard error of the prediction interval around the common estimate (0·87), both on the logit scale. This resulted in an average case fatality rate of 0·059 (median: 0·044, 95% credible interval: [0·008 - 0·196]). Thus, we captured the strong positive skew in the prediction interval from the random-effects meta-analyses.

2.2.2.3 Proportion of typhoid-related deaths occurring outside the hospital

Because we do not have enough evidence to inform an estimate for the overall case fatality rate and/or case fatality rate among outpatients and those who do not seek formal medical care, we assumed the proportion of typhoid deaths occurring among non-hospitalized patients (as opposed to hospitalized patients) varies between 0% and 75% (uniform distribution), implying that on average, approximately one out of three deaths (38%) occur outside the hospital setting (i.e. among outpatients or those not seeking formal medical care).

2.2.2.4 Case fatality rate among people infected with an antimicrobial resistant strain

We made a distinction between the case fatality rate among people infected with an antimicrobial resistant versus sensitive strain (see 'Antimicrobial resistance' section below).

2.2.2.5 Average age of death from typhoid fever

We assumed the same case fatality rate and healthcare use for all ages, based on the results of our meta-analysis.[20] Thus, the average age of death from typhoid equals the average age of typhoid fever cases. This can be derived directly from the output of the transmission-dynamic model, i.e. from the age-specific incidence of typhoid cases in the absence of vaccination. Specifically, the average age of death from typhoid fever for each country was calculated by summing of the midpoints of each of the 6 age groups multiplied by the proportion of typhoid cases in that age group. For the eldest age group, the midpoint between age 25 and the country-specific life expectancy was used. The country with lowest average age of death from typhoid is Yemen (mean: 10 months, median: 11 months, 95% credible interval: [7-43]). The country with the highest average age of death from typhoid is Tajikistan (mean and median: 37 years, 95% credible interval: [35-37] years).

2.2.3 Healthcare use

2.2.3.1 Probability of seeking professional medical care

Numerous studies have attempted to estimate the probability of seeking care for typhoid fever through the use of healthcare utilization surveys, e.g.[21-23] However, these surveys typically ask about hypothetical behavior and/or care-seeking for fever in general, and the responses can be very heterogeneous and difficult to interpret. Instead, we based our estimate of the probability of seeking care for typhoid fever on the posterior distribution obtained by fitting a model to incidence data from passive versus active surveillance.[10] Furthermore, this approach is more consistent with the estimates of typhoid fever incidence that we use. The model estimates the difference in the reported number of typhoid cases between active and passive surveillance, assuming that active surveillance captures all cases of typhoid fever. The estimated probability of seeking care was 0·57 (95% credible interval: 0·42-0·77), which we used for all countries. To account for the uncertainty of this parameter, we sampled directly from its posterior distribution.

2.2.3.2 Probability of hospitalization

Eleven studies identified in a systematic review by Mogasale et al[24] reported on the probability of hospitalization among those with typhoid fever. To estimate the typhoid hospitalization rate for our cost-effectiveness analysis, we obtained the typhoid hospitalization rate from each of these studies as the number of patients hospitalized among blood-culture confirmed typhoid cases during the surveillance period (Appendix Table 8). To account for differences in surveillance method (active versus passive surveillance), the number of confirmed typhoid cases in studies with passive surveillance was augmented according to the probability of seeking professional medical care (see previous section), which resulted in the 'adjusted cases' in Appendix Table 8. We sought to explain the large heterogeneity between the studies by exploring relationships between country-specific and within-country differences in the reported hospitalization rates (see Appendix Table 9 below for the complete list of factors explored). However, none of the included variables explained the heterogeneity in a significant way (for details, see Abboud[25]).

Appendix Table 8: Description of studies included in the meta-analysis of the probability of hospitalization for typhoid fever (reprinted from Abboud Linda, University of Antwerp Master's thesis, 2017 [25]).

Country	Location	Article [source]	Year	Cases*	Hosp-italized	Adjusted cases**
Bangladesh	Kamalapur, Dhaka	Brooks (2005) [26]	Dec 2000-Oct 2001	49	0	49
		Naheed (2010) [27]	Jan 2003-Jan 2004	40	4	40
China	Hechi, Guangxi Zhuang	Ochiai (2008) [28]	Aug 2001-Jul 2002	15	6	26
Egypt	Bilbeis district	Crump (2003) [29]	Jul 2011-Oct 2011#	19##	6	33
	Fayoum Governorate	Srikantiah (2006) [30]	Jun 2002-Oct 2002	90	30	158
Indonesia	Tanjung Priok & Koja, North Jakarta	Punjabi§ (2013) [31]	Aug 2001-Jul 2003	221	4	388
India	Kalkaji	Sinha (1999) [32]	Nov 1995-Oct 1996	63	6	63
		Bahl (2004) [33]	Nov 1995-Feb 1997	35	5§§	61
	Kolkata	Sur° (2006) [34]	Jan 2004-Dec 2004	95	2	95
Kenya	Kibera	Breiman (2012) [35]	Mar 2007-Feb 2009	135	2	135
	Lwak	Breiman (2012) [35]	Oct 2007-Sep 2009	22	5	22
Pakistan	Hijrat, Sultanabad, and Bilal colonies in Karachi	Ochiai°° (2008) [28]	Aug 2002-Jul 2004	189	3	189
	Rehri Goth| Coastal Karachi	Owais (2010) [36]	Feb 2007-May 2008	16	0	16
Vietnam	Hue	Ochai (2008) [28]	Jun 2002-Jun 2003	18	5	32

*Blood-culture confirmed typhoid cases
**Adjusted total number of cases for surveillance type (multiplication factor for passive surveillance

studies=0.56981)
#In Crump (2003), the time of the survey is indicated as Aug 2000-Jan 2001, during which four months of surveillance was conducted. However, the exact dates of the surveillance are not specified. As such, the date in Mogasale (2016) is assumed as the correct period.
##In this article, the authors adjusted for healthcare provider sampling. For this analysis, the unadjusted total was used.
§For North Jakarta, the information listed in Punjabi (2013) was chosen because it covers a larger period than the article in Mogasale (2016).
§§The result was calculated according to the assumption that Sinha (1999) is representing and equal to the active surveillance arm included in Bahl (2004). Given that from the total of 98 cases, 11.1% were hospitalized, we calculated that there were 11 hospitalized cases. Removing the six hospitalized cases mentioned in Sinha (1999), five hospitalizations remain for the passive surveillance arm of the study.
°Data extraction from original article Sur (2006).
°°Data extraction from original article Ochiai (2008).
ǀThe specific name of the coastal site was not mentioned in the text and was extracted from Kotloff (2012).

Appendix Table 9: Factors potentially associated with typhoid hospitalization rate (reprinted from Abboud Linda, University of Antwerp Master's thesis, 2017 [25]).

Factor	Definition
**National factors***	
National health access factors	
Out-of-pocket (% of health expenditure)	Any direct outlay by households, including gratuities and in-kind payments, to health practitioners and suppliers of pharmaceuticals, therapeutic appliances, and other goods and services whose primary intent is to contribute to the restoration or enhancement of the health status of individuals or population groups. It is a part of private health expenditure.
Nurses and midwives (per 1,000 people)	Include professional nurses, professional midwives, auxiliary nurses, auxiliary midwives, enrolled nurses, enrolled midwives and other associated personnel, such as dental nurses and primary care nurses.
Physicians (per 1,000 people)	Include generalist and specialist medical practitioners.
Health expenditure per capita, PPP	The sum of public and private health expenditures as a ratio of total population. It covers the provision of health services (preventive and curative), family planning activities, nutrition activities, and emergency aid designated for health but does not include provision of water and sanitation. Data are in international dollars converted using 2011 purchasing power parity (PPP) rates.
Health expenditure, total (% of GDP)	The sum of public and private health expenditure. It covers the provision of health services (preventive and curative), family planning activities, nutrition activities, and emergency aid designated for health but does not include provision of water and sanitation.
Hospital beds (per 1,000 people)	Include inpatient beds available in public, private, general, and specialized hospitals and rehabilitation centres. In most cases beds for both acute and chronic care are included.
Other national factors	
HIV prevalence	The percentage of people ages 15-49 who are infected with HIV.
Gini index	Measures the extent to which the distribution of income (or, in some cases, consumption expenditure) among individuals or households within an economy deviates from a perfectly equal distribution. A Gini index of 0 represents perfect equality, while an index of 100 implies perfect inequality.
**Subnational factors****	
Development factors	
Population density	Population density (persons per kilometer).
Poverty_iwi50	% of households with an International Wealth Index (IWI) value under 50. An IWI is an asset based wealth index. An IWI of 0 represents no

	assets, while an IWI of 100 represents all assets.
GDP per capita	Gross domestic product (GDP) per capita (adjusted to international dollars based on PPP).
Flush toilets	% of households with flush toilet in region.
Child factors	
Stunting prevalence	Percentage of children aged 0-59 months who are below minus two standard deviations (SD) from median height-for-age of the WHO Child Growth Standards.
Underweight prevalence	Percentage of children aged 0-59 months who are below minus two SD from median weight-for-age of the WHO Child Growth Standards.
Wasting prevalence	Percentage of children aged 0-59 months who are below minus two SD from median weight-for-height of the WHO Child Growth Standards.
Other subnational factors	
Women education 20+	Mean years education of women aged 20+ in region.
Piped water	% of households with piped water in region.
Under 5 mortality rate	Number of children dying under five year of age, per 1000 live births in a given year.
**Site-specific factors**	
Km2 of site area	The area calculated for the study location.
Number of health facilities	The number of health facilities included in the study.
Healthcare density	The density of health facilities in the study site.
Population density in site	The manually calculated population density of the study site, dividing the total population by site area Km^2 (The number total population is reported by the authors from census).
Adjusted incidence	Typhoid incidence of the site, adjusted for surveillance type.

*All national data and definitions were extracted from World Bank database (World Development Indicators [37])

**All sub-national data and definitions were extracted from GDL website [38]

Therefore, we decided to use the country-specific hospitalization rates for the four Gavi-eligible countries for which information was available (Appendix Table 10, estimates are based on a random-effects model including all studies for each country):

Appendix Table 10: Country-specific probability of hospitalization for typhoid fever used in our cost-effectiveness analysis.

	Value (median (mean) [95% CI])	Uncertainty distribution	Source
**Bangladesh**	0.024 (0.062)[3e-4-0.65]	$logit^{-1}$ (normal(mean=-3.72, se=1.62))	[26, 27]
**India**	0.058 (0.062) [0.021-0.15]	$logit^{-1}$ (normal(mean=-2.78, se=0.37))	[32-34]
**Kenya**	0.059 (0.090) [0.002-0.67]	$logit^{-1}$ (normal(mean=-2.77, se=1.09))	[35]
**Pakistan**	0.015 (0.016) [0.005-0.04]	$logit^{-1}$ (normal(mean=-4.21, se=0.58))	[28, 36]

For countries for which no data was available, we used a common estimate of 0·038 with 95% prediction interval [0·004-0·260], based on a random-effects meta-analysis including only the studies conducted in Gavi-eligible countries (Bangladesh, India, Kenya and Pakistan); the resulting estimate was slightly lower than when including all studies shown in Appendix Table 8 above (0·06 [0·00-0·48]). Uncertainty around the probability of hospitalization for typhoid was characterized using the inverse logit of a normal distribution with mean equal to the common estimate from the random-effects meta-analysis, and standard error equal to the standard error based on the prediction interval around the common estimate, both on the logit scale. This resulted in an average probability of hospitalization for typhoid of 0·038 (0·038) [0·004-0·249] (median (mean) [95% credible interval]. Thus, we captured the strong positive skew in the prediction interval from the random-effects meta-analyses.

2.2.3.3 Length of stay in the hospital

Three studies reported on the length of stay in the hospital due to typhoid fever. We combined the estimates of Sur et al[39] (India) and Riewpaiboon et al[40] (Tanzania) in a random effects meta-analysis to estimate the mean length of stay in the hospital for countries without data. We did not include the estimate of Shahunja et al[41] (Bangladesh) as it gave no information on the standard error of the mean; however, our estimate of 6 days (95% prediction interval [3-9] days) is the same as the reported median of 6 days for Bangladesh.[41] Uncertainty around the average length of stay in the hospital for typhoid was characterized by a gamma distribution with mean equal to the common estimate from the random-effects meta-analysis and standard error equal to the standard error based on the prediction interval around the common estimate. For India and Tanzania, we used the country-specific estimates, but multiplied the standard error by two to acknowledge the additional uncertainty of extrapolating information from a single setting to the country as a whole.

2.2.3.4 Number of visits to a medical doctor

We assumed a single visit to a medical doctor during an episode of typhoid fever, for both inpatients and outpatients. Although this assumption is not based on any evidence, we did not account for its uncertainty, as the costs associated with a visit to a medical doctor are low and therefore highly unlikely to impact the results significantly. Indeed, Drummond et al [42] stated (p.57) that it is not worth investing a great deal of time and effort considering costs that, because they are small, are unlikely to make any difference to the study results.

2.2.4 Antimicrobial resistance

2.2.4.1 Proportion of typhoid infections that are resistant to antimicrobial treatment

We assumed that the proportion of typhoid fever cases that are caused by an infection with an antimicrobial resistant (AMR) strain is on average 50% and varies uniformly between 0% (i.e. no cases infected by a resistant strain) and 100% (i.e. all typhoid cases due to AMR strains). The large uncertainty range was chosen to reflect the absence on information on this for most countries and its tendency to change rapidly over time.[43] Thus, we acknowledge and investigate the importance of the uncertainty around the proportion of typhoid cases caused by an AMR strain. The same assumption was applied to all countries.

2.2.4.2 Burden of antimicrobial resistant relative to sensitive cases

In the absence of studies that compare disease burden and treatment costs for antimicrobial resistant and sensitive cases, we assumed that for an AMR case, the probability of death, the average years of life lived with disability, and the average treatment cost is on average twice that of an antimicrobial sensitive case, and varies uniformly between 1 and 3 times the burden of sensitive cases. Thus, to calculate the life-years lost due to premature death, the years of life lived with disability, and the treatment cost for an average AMR case, we multiplied the probability of death, years of life lived with disability, and the treatment cost for an average antimicrobial sensitive strain by a uniformly distributed random variable, $X \sim \text{Uniform}(1,3)$. As such, our assumption for case fatality rate is in line with what was found by Pieters et al[20]: i.e. the odds of dying from infection with an AMR strain were almost twice as high compared to the odds of dying from infection with a susceptible strain (OR: 1·73, 95% CI: 0·69-4·33, p=0·1840, n=6/44), although this was not statistically significant. We applied this multiplication factor to the average treatment cost rather than the duration of hospitalization or the cost per bed-day in the hospital, as applying it to both would

result in quadrupling the average hospitalization cost of an antimicrobial resistant versus sensitive case.

2.2.5 Costs conversion and inflation

All costs were converted to a common year price level (i.e. 2016) in 2 stages: first, costs in their original currencies were converted to US dollars by using WHO exchange rates[44]. Then costs were inflated based on the consumer price index to the 2016 price level (https://inflationdata.com/Inflation/Consumer_Price_Index/HistoricalCPI.aspx?reloaded=true#Table?reloaded=true).

2.2.6 Treatment costs

2.2.6.1 Estimates for India, Tanzania, and Pakistan

The average direct medical costs for treating an inpatient and outpatient for typhoid fever from the healthcare provider's perspective is available from sites in India,[39, 45] Tanzania,[40] and Pakistan.[45] Bahl et al[33] also reported on treatment costs for typhoid fever for India, but the reported cost per bed-day in the hospital was very high (2000 INR in 1996=US$78 in 2010) compared to the average cost per bed-day for any disease in India (US$7 in a private teaching hospital, US$8 in a district hospital, US$12 in a tertiary care hospital, US$38 in a charitable hospital, and US$135 in a private hospital, in 2010 USD[46]). Thus, we surmised that the patients from the Bahl et al study[33] were referred to an expensive private hospital and therefore did not include it.

From Sur et al,[39] we used the unadjusted costs for all ages combined, which included the costs for drugs and lab tests (Table 4 in [39]). Although Riewpaiboon et al[40] stated that they used the societal (patient) perspective (which includes direct medical costs, direct non-medical costs, and productivity loss), they also stated that they used treatment costs from the hospital's perspective to estimate the direct medical costs, which corresponds to the healthcare provider's perspective. For the outpatient costs, we considered only the costs that were reported to be incurred before hospitalization. From Poulos et al,[45] we used the provider costs except for Pakistan, for which only net public costs (i.e. provider minus patients' payments) were available. Note that there was almost no reported difference between the provider and net public costs.[45]

To characterize the uncertainty in the inpatient and outpatient treatment costs, we doubled the standard error around the average cost for inpatient and outpatient treatment for Tanzania to account for the additional uncertainty that comes from extrapolating data from a single setting to the whole country. For Pakistan, Poulos et al[45] reported no costs for outpatient treatment; thus, we assumed the outpatient treatment costs were fixed at US$0. For India, the final estimate was based on a fixed effects model including both studies,[39, 45] with the uncertainty range based on the 95% confidence interval around the common estimate.

For India, we used the average cost of drugs for treating outpatients with typhoid fever as a proxy for the treatment cost of typhoid patients not seeking professional medical care ($1·43, 95% UI: [0·93-2·04] in 2016 USD).[39] For Tanzania and Pakistan, we use the same average cost for typhoid patients not seeking professional medical care as for the other countries (see next section).

2.2.6.2 Estimates for other countries

For all other Gavi-eligible countries, we estimated country-specific treatment costs for typhoid fever as follows:

treatment cost for inpatients with typhoid fever

= cost per bed-day * length of stay in hospital + cost lab tests inpatient + cost drugs inpatient + adjustment factor*cost outpatient visit

treatment cost for outpatients with typhoid fever
= cost drugs outpatient + adjustment factor*cost outpatient visit

treatment cost for patients with typhoid fever not seeking formal medical care
= cost drugs outpatient

Our estimates for the length of stay in hospital and the number of outpatient visits for an inpatient and outpatient are detailed above (sections 2.2.3.3 and 2.2.3.4). The next sections detail how we estimated the other components of the treatment cost for inpatients and outpatients with typhoid fever.

2.2.6.2.1 Average cost per bed-day and average cost per outpatient visit

We calculated the average cost per bed-day and per outpatient visit for each Gavi-eligible country based on the estimates from the WHO-CHOICE 2010 prediction model, and validated our estimates by comparing them to published data not used for the WHO-CHOICE 2010 prediction model. The WHO-CHOICE 2010 prediction model estimates the average cost per bed-day in a primary level hospital for *any disease* and the average cost per outpatient visit in a healthcare center (no beds) for *any disease* for all countries [44].

a) No estimates were available for Somalia and Zimbabwe; for these countries, we conservatively assumed the same cost as the neighboring country with the lowest price (i.e. Ethiopia for Somalia and Mozambique for Zimbabwe). For the Democratic People's Republic (DPR) of Korea, no estimates were available; for this country, we conservatively assumed the same costs as the country with the lower treatment costs. To characterize the uncertainty in treatment costs for Somalia, Zimbabwe and DPR Korea, we doubled the standard errors of the average cost per bed-day and per outpatient visit for these countries, since we are extrapolating from data for other countries.
b) For four countries, we identified separate publications (i.e. not used to build the WHO-CHOICE prediction model) on the average cost per bed-day and per outpatient visit for *any disease* in a district/provincial or primary referral hospital: Vietnam [47], Tanzania [48], India [46], and Kenya [49] (only per bed-day cost). We used these four studies to validate the estimates of the WHO-CHOICE 2010 prediction model. Appendix Table 11 shows that the WHO-CHOICE 2010 cost estimates were slightly lower but comparable to the published costs for India, Kenya, Tanzania and Vietnam.
c) To characterize the uncertainty in the costs per bed-day and per outpatient visit, we doubled the standard errors to acknowledge the additional uncertainty of using the unit costs per bed day and outpatient visit for *any disease* as a proxy for the unit costs for *typhoid fever*.
d) For the adjustment factor applied to the average treatment cost for an outpatient, see below.

Appendix Table 11: Cost per bed day and outpatient visit for *any disease* in a district/provincial or primary referral hospital. **Comparison of costs based on the WHO-CHOICE prediction model and recent country-specific publications. Costs are presented in USD2010.**

Country (source)	cost per bed day<br>WHO-CHOICE 2010 estimate[44]	recent publication	cost per outpatient visit<br>WHO-CHOICE 2010 estimate[44]	recent publication
**Kenya[49]**	$5 [2-12]	$11	$1.1 [3.4-0.2]	NA
**Tanzania[48]**	$4 [2-9]	$8	$0.9 [0.2-2.7]	$3.6
**Vietnam[47]**	$9 [4-20]	$16	$2 [0.3-4.7]	$4.1
**India[46]**	$11 [4-24]	$9	$1.5 [0.4-5.2]	$2.1

2.2.6.2.2 Average cost for drugs and lab tests

Next, we obtained cost estimates for drugs and lab tests *for typhoid fever* in inpatient and outpatient settings. Only one study[39] reported costs for drugs (US$9·71, standard deviation (SD): US$10·42 for inpatients and US$1·30, SD: US$1·11 for outpatients in 2004) and lab tests (US$5·21, SD: US$14·14 for inpatients and US$0, SD: US$0 for outpatients in 2004). We used these estimates for all countries, but multiplied the standard errors by four to acknowledge the uncertainty from (i) extrapolating data from a setting to the whole country and (ii) extrapolating data from one country to another country. Based on Sur et al,[39] we assumed no lab test for outpatients. Additionally, Poulos et al[45] reported no costs for outpatients with typhoid fever in Pakistan (meaning no costs for drugs or lab tests). Therefore, we assumed that the cost for drugs for outpatients varies between 0% and 100% of the cost for drugs measured by Sur et al[39] for India.

2.2.6.2.3 Adjustment factor for cost of an outpatient visit

We added an adjustment factor to the average cost of an outpatient visit for typhoid fever, based on a validation exercise we did. To validate our estimates of the average treatment costs for inpatients and outpatients with typhoid fever in other countries, we compared these average treatment cost estimates calculated for India, Tanzania, and Pakistan to those of Sur et al[39], Riewpaiboon et al[40], and Poulos et al[45] (see 'Estimates for India, Tanzania and Pakistan' above). Additionally, we included the estimates of two non-Gavi-eligible countries that are available from Poulos et al[45] (Indonesia and Vietnam). Appendix Table 12 shows that our estimated costs for inpatients with typhoid fever based on the WHO-CHOICE 2010 prediction model seem to underestimate the average treatment cost of a typhoid inpatient for Indonesia, India, Vietnam, and to overestimate the cost for Pakistan and Tanzania. However, all confidence intervals overlap with the ones of the typhoid-specific studies. Our estimated treatment costs for typhoid outpatients based on the WHO-CHOICE 2010 prediction model seem to overestimate the average cost of typhoid fever in an outpatient setting, except for Indonesia; however, the confidence intervals overlap for all countries except Indonesia and Pakistan.

Appendix Table 12: Treatment costs for a typhoid fever episode in an inpatient and outpatient setting. Comparison of costs based on the WHO-CHOICE prediction model (for any disease) and typhoid-specific cost studies. Costs are presented in USD2010.

Country (source)	cost of inpatient treatment<br>WHO-CHOICE 2010 based estimate	typhoid-specific study	cost of outpatient treatment<br>WHO-CHOICE 2010 based estimate	typhoid-specific study
**Tanzania[40]**	$33 [9-92]	$20 [5-53]	$1.5 [0.4-3.5]	$0.8 [0.1-3.0]
**Vietnam[45]**	$66 [28-145]	$132 [77-207]	$2.2 [0.8-4.6]	$1.1 [0.6-1.8]
**Pakistan[45]**	$48 [18-114]	$19 [11-30]	$1.7 [0.5-3.9]	$0
**India[39]**	$88 [39-170]	$113 [96-132]	$2.4 [0.9-5.0]	$2.3 [2.0-2.6]
**Indonesia[45]**	$108 [53-217]	$198 [115-314]	$3.2 [1.4-5.9]	$14.1 [8.3-22.0]

To further explore the reasons for the difference in treatment costs in an outpatient setting, we compared the average costs for an outpatient visit for *any* disease (WHO-CHOICE 2010 prediction model) with the average cost for an outpatient visit for *typhoid fever* based on data for India and Tanzania. We found that the average costs per outpatient visit for *any disease* were higher than the reported average costs for an outpatient visit for *typhoid fever*. In India, the cost per outpatient visit for typhoid fever was $0·69,[39] compared to $5 per outpatient visit for any disease estimated by WHO-CHOICE. In Tanzania, the cost per outpatient visit was $1·1,[40] compared to $3·3 per outpatient visit for any disease estimated by WHO-CHOICE.

Based on this comparison, we included an adjustment factor to account for the possibility that the costs per outpatient visit for any disease estimated by the WHO-CHOICE prediction model overestimate the actual cost of treating a typhoid case in an outpatient setting. We assumed that the average cost for an outpatient visit for typhoid (from a healthcare provider's perspective) varies between 25% and 100% of that predicted by the WHO-CHOICE model. The impact of applying this adjustment factor for the data from the five countries with typhoid-specific data is shown in Appendix Table 13 below.

Appendix Table 13: Treatment costs for a complete typhoid fever episode in an inpatient and outpatient setting including the adjustment factor for the treatment cost of a typhoid outpatient. Comparison of costs based on the WHO-CHOICE prediction mode (for any disease) and typhoid-specific cost studies. Costs are presented in USD2010.

**Country (source)**	**cost inpatient** WHO-CHOICE 2010 based estimate	typhoid-specific study	**cost outpatient** WHO-CHOICE 2010 based estimate (adjusted)	typhoid-specific study
**Tanzania[40]**	$31 [9-99]	$20 [5-53]	$1.2 [0.3-3.0]	$0.8 [0.1-3.0]
**Vietnam[45]**	$65 [26-146]	$132 [77-207]	$1.6 [0.4-3.9]	$1.1 [0.6-1.8]
**Pakistan[45]**	$49 [16-127]	$19 [11-30]	$1.4 [0.3-3.4]	$0
**India[39]**	$86 [39-173]	$113 [96-132]	$1.7 [0.5-4.1]	$2.3 [2.0-2.6]
**Indonesia[45]**	$110 [53-219]	$198 [115-314]	$2.2 [0.8-4.8]	$14.1 [8.3-22.0]

2.2.6.2.4 Final estimates used for cost-effectiveness analysis

The final treatment cost for patients not seeking formal medical care was $0·84, 95% credible interval: [$0·03-2·44] in 2016USD. The final estimates for the treatment costs for typhoid inpatients and outpatients used in our analysis are shown in Appendix Table 14 for each Gavi-eligible country. Note that for Tanzania, Pakistan, and India, the country-specific estimates were used, whereas for all other countries, the estimates were based on the WHO-CHOICE unit cost data.

Appendix Table 14: Average treatment cost for a typhoid inpatient and outpatient for each Gavi-eligible country. We list the mean, median, and 95% credible interval (UI, lower and upper bound) of the treatment cost distributions used as input in our model. Costs presented in USD2016.

	**Inpatient treatment costs (US$)**				**Outpatient treatment costs (US$)**			
	**mean**	**median**	**lower 95% UI**	**upper 95% UI**	**mean**	**median**	**lower 95% UI**	**upper 95% UI**
**Afghanistan**	28	22	3	91	1.1	1	0.1	2.9
**Bangladesh**	48	42	14	119	1.5	1.3	0.3	3.3
**Benin**	54	48	17	121	1.6	1.5	0.4	3.7

**Burkina Faso**	48	42	13	117	1.5	1.4	0.3	3.5
**Burundi**	26	21	3	88	1.1	0.9	0.1	2.7
**Cambodia**	59	53	19	130	1.6	1.5	0.4	3.6
**Cameroon**	77	70	29	160	2	1.8	0.6	4.2
**Central African Republic**	39	34	10	104	1.4	1.2	0.3	3.3
**Chad**	55	49	17	127	1.6	1.5	0.4	3.6
**Comoros**	53	47	16	120	1.6	1.5	0.4	3.7
**Cote dIvoire**	70	62	26	149	1.9	1.7	0.5	4.1
**Dem Republic of Congo**	27	22	4	89	1.1	1	0.2	2.8
**Djibouti**	92	86	37	177	2.2	2.1	0.7	4.6
**DPR Korea**	48	41	13	118	1.5	1.3	0.3	3.5
**Eritrea**	27	21	3	89	1.1	1	0.1	2.7
**Ethiopia**	34	28	6	99	1.2	1.1	0.2	2.8
**Gambia**	58	53	19	128	1.6	1.5	0.4	3.7
**Ghana**	60	55	21	134	1.7	1.5	0.5	3.7
**Guinea**	37	31	8	102	1.3	1.2	0.2	3.1
**Guinea Bissau**	46	40	12	114	1.5	1.3	0.3	3.5
**Haiti**	54	48	17	130	1.7	1.5	0.4	3.7
**India**	125	125	106	147	2.5	2.5	2.2	2.8
**Kenya**	55	49	17	125	1.6	1.5	0.3	3.7
**Kyrgyz Republic**	63	57	20	138	1.7	1.6	0.4	3.7
**Lao PDR**	64	58	21	142	1.7	1.5	0.4	3.8
**Lesotho**	69	63	25	143	1.9	1.7	0.5	4.2
**Liberia**	31	26	5	95	1.2	1.1	0.2	2.9
**Madagascar**	33	28	6	97	1.2	1	0.2	2.9
**Malawi**	41	34	9	107	1.4	1.3	0.3	3.2
**Mali**	45	40	12	113	1.4	1.3	0.3	3.4
**Mauritania**	69	62	22	156	1.8	1.7	0.5	4
**Mozambique**	39	33	9	108	1.4	1.2	0.2	3.4
**Myanmar**	49	44	14	112	1.5	1.4	0.4	3.5
**Nepal**	41	35	10	108	1.3	1.2	0.2	3.3
**Nicaragua**	111	104	49	207	2.4	2.2	0.8	4.8
**Niger**	31	26	5	95	1.2	1.1	0.2	3.2
**Nigeria**	84	79	33	172	2.1	1.9	0.7	4.5
**Pakistan**	21	20	12	32	0	0	0	0
**Papua New Guinea**	119	112	52	222	2.7	2.5	0.9	5.5
**Republic of Sudan**	87	80	36	174	2.1	1.9	0.6	4.4
**Rwanda**	44	38	12	111	1.4	1.3	0.3	3.4
**Sao Tome e Principe**	63	58	22	138	1.7	1.6	0.5	4
**Senegal**	69	63	26	149	1.9	1.7	0.5	4
**Sierra Leone**	38	32	8	102	1.3	1.2	0.2	3.2
**Solomon**	145	136	67	265	3.2	3	1.1	6.4

**Islands**								
**Somalia**	34	28	6	98	1.2	1.1	0.2	3
**South Sudan**	86	81	36	169	2.1	1.9	0.6	4.4
**Tajikistan**	59	53	19	127	1.7	1.5	0.4	3.6
**Tanzania**	24	21	5	56	1.1	0.9	0.1	3.3
**Togo**	39	33	7	106	1.3	1.2	0.3	3.1
**Uganda**	43	37	12	112	1.4	1.3	0.3	3.5
**Yemen**	98	92	41	191	2.4	2.2	0.8	5
**Zambia**	57	52	19	131	1.7	1.5	0.4	3.8
**Zimbabwe**	39	33	8	106	1.4	1.2	0.3	3.3

2.2.7 Vaccine-related costs

We calculated the costs of vaccination for routine and catch-up campaign doses as follows:

total cost per dose routine vaccination =
vaccine procurement cost + vaccine injection and safety equipment cost + vaccine delivery cost routine start-up costs (only included for the first 1-3 years) + vaccine delivery cost routine ongoing cost

total cost per dose campaign =
vaccine procurement cost + vaccine injection and safety equipment cost + vaccine delivery cost campaign

2.2.7.1 Vaccine procurement cost per dose

We assumed a price of $1·50 per dose for all countries based on the recent announcement of the WHO pre-qualification of the Bharat Biotech Typbar-TCV.[50] In our primary analysis, we included only the costs to be paid by the country (i.e. excluding the Gavi support). Each country's contribution over time was informed by direct communication with Gavi. However, the country contributions we use in our analysis are simplified approximations and assume a country would introduce TCVs in 2019. As such, they do not necessarily reflect Gavi's real support over time for a given country.

Our assumptions on vaccine procurement costs can be summarized as follows:
For routine immunisation, the country's contribution for the vaccine price per dose and per year depends on the phase in which the country is:[51] Initial self-financing 'ISP', Preparatory transition 'PTP', Accelerated transition 'ATP', or fully self-financing, as follows:

- ISP: The country’s contribution was assumed to be 13% (i.e. $0·20 per dose because we used a total price of $1·50 per dose).
- PTP: The contribution in year one in PTP was assumed to be the same as in ISP, and then to increase gradually at a rate of 15% each year.
- ATP: The contribution in year one in ATP was assumed to be the same as the contribution in the last year in PTP. Beginning the second year in ATP, the contribution increases linearly up to 100% in five years. Countries that are assumed to start in ATP are assumed to contribute 20% of the total vaccine price in the first year.
- fully self-financing: the country does not receive Gavi-support.

Twenty-one countries were assumed to be in ISP for the next 10 years, 10 countries were assumed to go from ISP to PTP in the next 10 years, 5 countries were assumed to be in PTP for the next 10 years, 11 countries were assumed to go from PTP to ATP in the

next 10 years, 6 countries were assumed to be in ATP for the next 10 years, and 1 country was assumed to be fully self-financed. We are not allowed to disclose the more detailed country-specific information, as per our agreement with Gavi.

Vaccine doses for the one-time campaigns were assumed to be fully funded by Gavi (informed by communication with Gavi); hence, no costs were assumed for the countries in transition (but full costs were assumed for fully self-financing countries).

In scenario analysis, we assumed a cost of $1·50 per dose for all countries (i.e. including the costs that would be covered by Gavi support).[50]

2.2.7.2 Vaccine injection and safety equipment cost per dose

We based our estimates of the costs for injection and safety equipment on a study by Portnoy et al,[52] which reports on the lowest and highest supplies and freight price per dose between 2011 and 2020. Their estimates are based on the Gavi price forecast for 91 countries (Appendix B in Portnoy et al[52]). The lowest value of all countries considered was $0·21 and the highest value of all countries considered was $0·24. We assumed the lowest and highest values reflect the 95% confidence limits of a gamma distribution, and that this gamma distribution is symmetric around the mean. We are aware that minimum and maximum values are not the best parameters to base distributions on, but because the injection and safety equipment cost per dose is such as small part of the total vaccine cost per dose, and because the variation in this cost between countries is very small, this cost estimate is very likely not influential on the results. Therefore, we believe our approach is sufficient.

2.2.7.3 Vaccine delivery cost per dose for routine immunization

We searched the literature for original studies reporting on the incremental economic costs of implementing a new childhood vaccination program (i.e. not replacing an existing vaccine, such as replacing PCV7 by PCV13) in a Gavi-eligible country from the governmental health service provider perspective. We excluded studies looking at vaccines for teenagers, such as the ones against human papilloma virus. 'Incremental' refers to activities and resources that would not have occurred if the new vaccines had not been introduced. 'Economic' (as opposed to financial) includes opportunity costs. The following studies were excluded:
- Madsen et al[53] (rotavirus vaccination in Malawi) used secondary data, i.e. Malawi was used as a theoretical case and data were obtained from public data sources, including WHO and UNICEF.
- Hutton et al[54](vaccination against malaria in Tanzania) used the societal perspective.
- The EPIC study in Uganda (see the EPIC immunization costing studies, http://immunizationeconomics.org) used the public service provider perspective (including NGO service providers).
- The EPIC study in Moldova is not representative of a Gavi-eligible country.

Hence, our estimates of the vaccine delivery cost per dose for adding a new vaccine to an existing childhood vaccination program were based on the four studies shown in Appendix Table 15 below.

Appendix Table 15: Overview of the studies used to estimate the average delivery cost per dose for adding a new vaccine to an existing routine immunization program. **Costs that are ongoing (as opposed to start-up) are presented separately. Costs are presented in USD2016.**

country	vaccine	study design	delivery cost per dose (US$)	ongoing delivery cost per dose	% ongoing	reference

				(US$)		
**Benin**	PCV13 (3 doses)	retrospective	1.71	1.18	69%	EPIC
**Ghana**	PCV13 (3doses) + rotavirus (2 doses) + measles (2nd dose)	retrospective	2.61	1.31	50%	EPIC
**Zambia**	PCV10 (3 doses)	prospective	2.98	1.86	62%	EPIC
**Rwanda**	PCV13 (3doses)	retrospective	0.71	0.46	65%	[55]
**Rwanda**	rotavirus (3 doses)	prospective	0.56	0.41	72%	[55]

PCV: pneumococcal conjugate vaccine
EPIC: see the EPIC immunization costing studies, http://immunizationeconomics.org

These estimates were used as input for the corresponding countries (Benin, Ghana, Zambia, and Rwanda). To reflect the uncertainty around how representative the costs for introducing pneumococcal, rotavirus, and measles vaccines are for the cost of introducing typhoid vaccine, we assumed a standard error around the mean estimates of US$0·45, which is equal to the mean of the five estimates presented in Appendix Table 15. The proportion of vaccine delivery cost representing ongoing costs was taken directly from each of the five studies (Appendix Table 15). We varied the number of years for which start-up costs should be included. As no information was available in the literature, we assumed this to be between 1 and 3 years; we used a discrete distribution assigning equal probabilities to 1, 2, and 3 years.

For the countries for which no information was found on the delivery cost of introducing a new vaccine into an existing routine immunization program, we used the delivery cost averaged over the five data points available ($1·94 per dose, Appendix Table 15), but doubled the standard error because we extrapolated estimates from other countries. Also, we applied a WHO-region adjustment to account for the variation in delivery costs between WHO regions (as the data available were only from African countries). Atherly et al [56] estimated a regional weighted mean for the cost per dose for rotavirus vaccination. We used these weighted means to assess how different the other regions are likely to be from the African region. This resulted in the following adjustment factors: 1 (Africa), 0·87 (Southeast Asia), 0·45 (Western Pacific), 2·19 (Europe), 1·21 (Americas) and 1·30 (Eastern Mediterranean). We multiplied the mean delivery cost per dose of $1·94 by the region-specific multiplication factor to get a region-specific estimate, which is presented in Appendix Table 16 below (final input estimates by country for routine immunization and catch-up campaign doses). For the proportion of the vaccine delivery cost that is ongoing in these countries, we used the mean plus and minus the standard error proportion of all four countries for which data was available. The standard error already reflects the uncertainty in this proportion when using different vaccines, but we further doubled this standard error to acknowledge that we were extrapolating data to other countries.

For the primary analysis, Gavi support for delivery of routine doses at the year of introduction was excluded. This one-time support depends on the phase in which a country is in at the year of introduction (see section 2.2.6.1), i.e. $0·80 per dose for countries in the initial self-financing phase, $0·70 per dose for countries in preparatory transition, $0·60 per dose for countries in accelerated transition, and no support for fully self-financing countries.

Appendix Table 16: Vaccine delivery cost per dose for routine immunization and for a one-time catch-up campaign. **Mean, median, and 95% credible intervals (UI, lower and upper bounds) for each country used as input in our model are presented. Costs presented in USD2016.**

	routine immunization				catch-up campaign			
	mean	median	lower 95% UI	upper 95% UI	mean	median	lower 95% UI	upper 95% UI
**Afghanistan**	2.29	2.09	0.47	5.5	0.24	0.24	0.15	0.35
**Bangladesh**	1.53	1.4	0.31	3.68	0.41	0.4	0.23	0.62
**Benin**	1.7	1.67	0.91	2.75	0.41	0.4	0.23	0.62
**Burkina Faso**	1.76	1.61	0.36	4.23	0.56	0.55	0.38	0.77
**Burundi**	1.76	1.61	0.36	4.23	0.41	0.4	0.23	0.62
**Cambodia**	0.79	0.72	0.16	1.9	0.41	0.4	0.23	0.62
**Cameroon**	1.76	1.61	0.36	4.23	0.41	0.4	0.23	0.62
**Central African Republic**	1.76	1.61	0.36	4.23	0.41	0.4	0.23	0.62
**Chad**	1.76	1.61	0.36	4.23	0.41	0.4	0.23	0.62
**Comoros**	1.76	1.61	0.36	4.23	0.41	0.4	0.23	0.62
**Cote dIvoire**	1.76	1.61	0.36	4.23	0.25	0.23	0.09	0.49
**Dem Republic of Congo**	1.76	1.61	0.36	4.23	0.41	0.4	0.23	0.62
**Djibouti**	2.29	2.09	0.47	5.5	0.41	0.4	0.23	0.62
**DPR Korea**	1.53	1.4	0.31	3.68	0.41	0.4	0.23	0.62
**Eritrea**	1.76	1.61	0.36	4.23	0.41	0.4	0.23	0.62
**Ethiopia**	1.76	1.61	0.36	4.23	0.23	0.21	0.08	0.45
**Gambia**	1.76	1.61	0.36	4.23	0.41	0.4	0.23	0.62
**Ghana**	2.61	2.58	1.77	3.61	0.41	0.4	0.23	0.62
**Guinea**	1.76	1.61	0.36	4.23	0.36	0.35	0.19	0.56
**Guinea Bissau**	1.76	1.61	0.36	4.23	0.41	0.4	0.23	0.62
**Haiti**	2.13	1.95	0.43	5.12	0.41	0.4	0.23	0.62
**India**	1.53	1.4	0.31	3.68	0.41	0.4	0.23	0.62
**Kenya**	1.76	1.61	0.36	4.23	0.41	0.4	0.23	0.62
**Kyrgyz Republic**	3.86	3.52	0.78	9.27	0.41	0.4	0.23	0.62
**Lao PDR**	0.79	0.72	0.16	1.9	0.5	0.5	0.41	0.6
**Lesotho**	1.76	1.61	0.36	4.23	0.41	0.4	0.23	0.62
**Liberia**	1.76	1.61	0.36	4.23	0.41	0.4	0.23	0.62
**Madagascar**	1.76	1.61	0.36	4.23	0.41	0.4	0.23	0.62
**Malawi**	1.76	1.61	0.36	4.23	0.41	0.4	0.23	0.62
**Mali**	1.76	1.61	0.36	4.23	0.41	0.4	0.23	0.62
**Mauritania**	1.76	1.61	0.36	4.23	0.41	0.4	0.23	0.62
**Mozambique**	1.76	1.61	0.36	4.23	0.41	0.4	0.23	0.62
**Myanmar**	1.53	1.4	0.31	3.68	0.41	0.4	0.23	0.62
**Nepal**	1.53	1.4	0.31	3.68	0.41	0.4	0.23	0.62
**Nicaragua**	2.13	1.95	0.43	5.12	0.41	0.4	0.23	0.62
**Niger**	1.76	1.61	0.36	4.23	0.41	0.4	0.23	0.62
**Nigeria**	1.76	1.61	0.36	4.23	0.53	0.53	0.36	0.73
**Pakistan**	2.29	2.09	0.47	5.5	0.54	0.54	0.36	0.75
**Papua New Guinea**	0.79	0.72	0.16	1.9	0.41	0.4	0.23	0.62
**Republic of Sudan**	2.29	2.09	0.47	5.5	0.41	0.4	0.23	0.62
**Rwanda**	0.63	0.5	0.07	2.03	0.31	0.31	0.23	0.42

**Sao Tome e Principe**	1.76	1.61	0.36	4.23	0.41	0.4	0.23	0.62
**Senegal**	1.76	1.61	0.36	4.23	0.08	0.05	0	0.34
**Sierra Leone**	1.76	1.61	0.36	4.23	0.41	0.4	0.23	0.62
**Solomon Islands**	0.79	0.72	0.16	1.9	0.41	0.4	0.23	0.62
**Somalia**	2.29	2.09	0.47	5.5	0.41	0.4	0.23	0.62
**South Sudan**	2.29	2.09	0.47	5.5	0.41	0.4	0.23	0.62
**Tajikistan**	3.86	3.52	0.78	9.27	0.41	0.4	0.23	0.62
**Tanzania**	1.76	1.61	0.36	4.23	0.34	0.34	0.26	0.44
**Togo**	1.76	1.61	0.36	4.23	0.41	0.4	0.23	0.62
**Uganda**	1.76	1.61	0.36	4.23	0.63	0.63	0.54	0.74
**Yemen**	2.29	2.09	0.47	5.5	0.41	0.4	0.23	0.62
**Zambia**	2.97	2.93	2.09	4.02	0.03	0.01	0	0.17
**Zimbabwe**	1.76	1.61	0.36	4.23	0.41	0.4	0.23	0.62

2.2.7.4 Vaccine delivery cost per dose for campaign

In our primary analysis, we excluded the Gavi support for delivery of campaign doses. This one-time support depends on which phase a country is in at the year of introduction (see section 2.2.6.1), i.e. $0·65 per targeted person for countries in the initial self-financing phase, $0·55 per targeted person for countries in preparatory transition, $0·45 per targeted person for countries in accelerated transition, and no support for fully self-financing countries.

In scenario analysis, we included the full delivery cost for campaign doses (including Gavi support). To estimate the vaccine delivery cost per dose for catch-up campaigns, we relied on a recent literature review by Gandhi and Lydon.[57] They examined the operational cost per dose for Supplementary Immunization Activities (SIAs) from 1992 to 2012. They included 17 studies with primary source data on SIAs from low- and middle-income countries. Most (but not all) of these studies reported retrospectively on the incremental or average costs for SIAs in children <5 years of age from the national healthcare payer perspective. Note that SIAs are usually conducted in addition to routine immunization, and therefore no big differences between incremental and average costs are expected. For comparability, the authors excluded opportunity costs, indirect costs, costs of vaccines, injection supplies, freight and custom charges, and international technical assistance to support the SIAs, including agency overhead costs. Eight studies reported on SIAs for measles (in combination with Vitamin A for one study and in combination with deworming and nutrition screening for another); the other studies reported on meningitis vaccine (MenA+C) (n=3), cholera vaccine (n=2), oral polio vaccine, tetanus toxoid vaccine, yellow fever and yellow fever in combination with MenA+C vaccine (n=1 for each).

Estimates for the vaccine delivery costs for SIAs were available for 13 countries.[57] To inform the standard error around these estimates, we used the standard error of the mean estimate for all 13 countries (=$0·05) to account for using other vaccines as a proxy for typhoid vaccine. For countries for which data were restricted to specific regions/districts (7 out of 13 studies), the standard error was further doubled to account for extrapolation from the region/district to the country level (Appendix Table 17).

For countries for which no data on the costs of SIAs were available, we used the same estimate, i.e. the mean of all 13 studies (US$0·41 ± 0·05). All operational costs per person per dose were approximately US$0·50 (median $0·33, range $0·03-0·79), and all were under US$1 (2010USD). There were no clear differences in the cost per dose between the different vaccines, and the variation in costs seemed larger within a region than between regions. We further doubled the standard error to acknowledge the uncertainty associated with extrapolating from other countries (Appendix Table 16).

Another study[58] estimated the cost per immunized child of adding measles into the routine program in Benin to be more than three times higher than the cost per immunized child for a campaign (US$0·63 vs US$1·95 in US$2011). Thus, we believe our estimates of the relative costs for routine versus campaign doses to be reasonable (Appendix Table 16). Nevertheless, we explored the impact of assuming vaccine delivery cost for campaign doses to be equal to the delivery costs of routine doses in scenario analysis (Appendix Table 27 andAppendix Table 28).

Appendix Table 17: Uncertainty distributions for country-specific vaccine delivery cost, as used in our cost-effectiveness analysis

	Uncertainty distribution
**Afghanistan**	gamma(mean=0.24,SE=0.05)
**Lao PDR**	gamma(mean=0.50,SE=0.05)
**Rwanda**	gamma(mean=0.31,SE=0.05)
**Uganda**	gamma(mean=0.63,SE=0.05)
**Tanzania**	gamma(mean=0.34,SE=0.05)
**Zambia**	gamma(mean=0.03,SE=0.05)
**Burkina Faso**	gamma(mean=0.56,SE=0.05*2)
**Côte d'Ivoire**	gamma(mean=0.25,SE=0.05*2)
**Ethiopia**	gamma(mean=0.22,SE=0.05*2)
**Guinea**	gamma(mean=0.36,SE=0.05*2)
**Nigeria**	gamma(mean=0.53,SE=0.05*2)
**Pakistan**	gamma(mean=0.54,SE=0.05*2)
**Senegal**	gamma(mean=0.08,SE=0.05*2)

2.2.8 Disability-adjusted life-years

2.2.8.1 Disability weights

To estimate the disability associated with a case of typhoid fever, we used the disability weights for acute infectious diseases (mild, moderate, and severe) from the 2010 Global Burden of Disease (GBD) study,[59] rather than the more recent GBD 2013 study.[60] The only difference (for infectious diseases) between the two GBD studies is that a large European respondent sample was added to the GBD 2013 study; this produced different disability weight estimates compared to the GBD 2010 study. However, as our study is focused on low- and middle-income countries, we decided the GBD 2010 estimates were more representative of disability associated with acute infectious disease episodes in these settings.

We based the disability weight for a moderate episode of typhoid fever on the disability weight of an "infectious disease, acute episode, moderate", which is characterized as 'has a fever and aches and feels weak, which causes some difficulty with daily activities', and is estimated to be 0·053 ± 0·012.[59] For a severe episode of typhoid fever, we based the disability weight on an "infectious disease, acute episode, severe", which is characterized as 'has a high fever and pain, and feels very weak, which causes great

difficulty with daily activities', and is estimated to be 0·21 ± 0·.04.[59] For a mild episode of typhoid fever, we based the disability weight on an "infectious disease, acute episode, mild", which is characterized as 'has a low fever and mild discomfort, but no difficulty with daily activities', and is estimated to be 0·005 ± 0·002.[59]

The difficulty lies in relating these disability weights to the model-predicted number of hospitalized cases, outpatients, and patients not seeking medical care. We consulted expert colleagues on typhoid fever, but their answers differed. Therefore, we decided to acknowledge the uncertainty around how to assign disability weights to the different healthcare use groups. Specifically, we considered two options:

Option 1:
- typhoid inpatient = disability weight for infectious disease, acute, severe
- typhoid outpatient = disability weight for infectious disease, acute, moderate
- typhoid patient not seeking medical care = disability weight for infectious disease, acute, mild

This results in an average disability weight of 0·04.

Option 2:
- typhoid inpatient = disability weight for infectious disease, acute, severe
- typhoid outpatient = disability weight for infectious disease, acute, severe
- typhoid patient not seeking medical care = disability weight for infectious disease, acute, moderate

This results in an average disability weight of 0·15, which is similar to that of another recent cost-effectiveness analysis of TCV introduction.[61]

In our analysis, we assumed that each of the two options has a 50% probability (i.e. half of the simulations were run assuming option 1 and the other half of the simulations were run assuming option 2). The same disability weights were used for all countries.

2.2.8.2 Duration of illness

We identified seven estimates of the average duration of illness for a person with typhoid fever based on three studies.[33, 40, 45] These studies included both outpatients and hospitalized patients. We found no clear relationship between the proportion of the study population hospitalized and the reported duration of illness. Also, we found no difference between the average duration of illness for children and adults with typhoid fever. Therefore, we used a single estimate for the duration of illness of 16 ± 2 days for patients seeking medical care based on a random effects model including all seven estimates. The credible interval is based on the prediction interval of the common estimate. We used the same value for all countries.

We assumed individuals with typhoid not seeking medical care had an average duration of illness of 8 days (i.e. half the length of persons seeking medical care), and varied this percentage between 0% and 100% (hence the duration of illness for a person not seeking medical care was varied uniformly between 0 and 16 days). The same assumption was used for all countries.

2.2.8.3 Years of life lost due to typhoid fever

We obtained the life expectancy at birth for each country from the World Bank 2014 (https://data.worldbank.org/indicator/SP.DYN.LE00.IN, latest access May 22 2018). To calculate the years of life lost due to typhoid fever, we subtracted the average age of death from typhoid fever from the life expectancy from that country (see section 'Average age of death from typhoid fever'). With this approach, we might slightly underestimate the years of life lost due to typhoid fever, since the average life

expectancy at 1 year of age (or at the average age of typhoid deaths) may be larger than the average life expectancy at birth if there is a high probability of death during the first year of life (i.e. high infant mortality rate). However, in this case, our approach is conservative (i.e. would bias against vaccine adoption).

2.3 Cost-effectiveness analysis

2.3.1 Determining the optimal strategy for each country

We used the net benefit framework to compare the costs and disability-adjusted life-years (DALYs) of the three vaccination strategies (routine vaccination with one dose of TCV at 9 months of age, hereinafter referred to as strategy *R*, or routine vaccination combined with a catch-up campaign to either age 5 (*RC5*) or 15 years (*RC15*)) versus no vaccination. Hence, our measure of cost-effectiveness is incremental net monetary benefit (and not the incremental cost-effectiveness ratio (ICER)), because determining the optimal strategy for more than two decision options in the context of uncertainty is not straightforward when using the ICER[62]. Using the net benefit framework, the optimal strategy among the four strategies compared is the strategy that results in highest *average* net monetary benefit (NB) for a given willingness-to-pay (WTP) value.[63]

Net monetary benefit was calculated as NB = ΔE*WTP - ΔC, where ΔE is the DALYs averted through a vaccination strategy compared to no vaccination, and ΔC is the incremental costs of a vaccination strategy compared to no vaccination. Because we accounted for uncertainty with probabilistic sensitivity analysis (PSA, see section 2.3.2), we obtained 2,000 NB values for each strategy. The strategy with the highest *average* NB (i.e. average from the 2,000 values) for a given WTP value is identified as the optimal strategy.

Due to the absence of a generally agreed-upon WTP threshold to define whether an intervention is 'cost-effective' in a given country,[64] we identified for each country (1) the optimal strategy for a range of WTP values, both in absolute terms (ranging between US$0-US$13,000 per DALY averted) as well as relative to each country's gross domestic product (GDP) per capita (between 0-4 times GDP per capita per DALY averted), and (2) the minimum WTP value for which any vaccination strategy becomes optimal.

2.3.2 Probabilistic sensitivity analysis ('PSA')

With probabilistic sensitivity analysis, a set of input values is drawn by random sampling one value independently from the probability distribution of each uncertain input parameter (presented in Tables 1-2 in main text). The model is then 'run' to obtain the corresponding cost-effectiveness outputs (i.e. the corresponding costs and DALYs for each strategy). This is repeated many times (in our case 2,000 times), generating 2,000 sets of input values with 2,000 corresponding costs and DALYs for each strategy. Hence, instead of a single estimate for the costs and DALYs for a particular strategy, PSA generates a distribution of costs and DALYs, reflecting the uncertainty around the cost and DALYs associated with a strategy.

2.3.3 Determining the uncertainty around the optimal strategy for each country

The percentage of the 2,000 samples for which a given strategy has the highest net benefit among all strategies reflects how certain we are that a strategy is optimal. This is presented for all countries in Tables 3-4 (main text) and in a cost-effectiveness acceptability frontier (CEAF) for each country (https://ceatyphoid.uantwerpen.be/home/).[62] Appendix Figure 3 shows the CEAF for India as an example. Based on this CEAF, we can see that in India, the optimal strategy is routine vaccination with a catch-up campaign up to 15 years of age (green line) if we are willing to pay at least US$300 per DALY averted. The probability that this strategy

results in the highest net benefit is only 45% at a willingness-to-pay of US$300 per DALY averted, i.e. we are 45% certain that routine vaccination with a catch-up campaign up to 15 years of age will result in higher net benefit than the other strategies. Note that for this strategy (routine + campaign up to 15 years), the probability to result in highest net benefit increases with increasing WTP values.

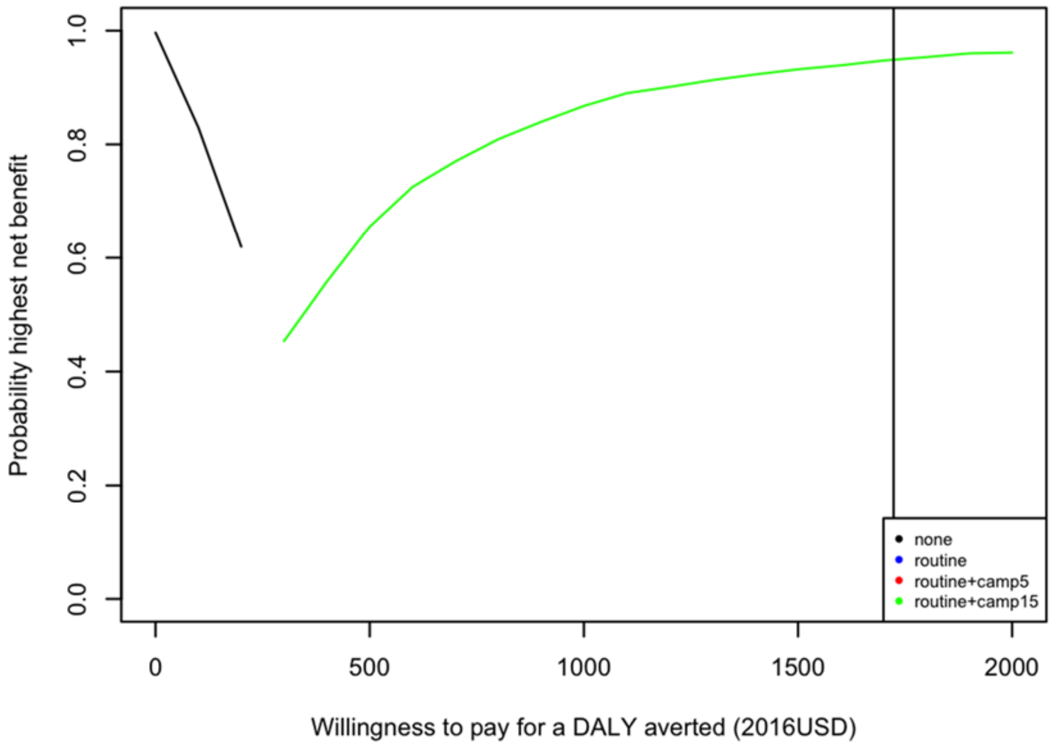

Appendix Figure 3: Cost-effectiveness acceptability frontier for India, showing for a range of willingness-to-pay values (X-axis), the **optimal** strategy (colour) and how certain we are about the **optimal** strategy (Y-axis). The vertical black line shows the GDP per capita for India.

2.3.4 Identifying the key drivers of uncertainty with EVPPI

For each country, we estimated the expected value of partially perfect information (EVPPI) for each uncertain input parameter.[65] The EVPPI is the maximum price that a healthcare decision-maker would (in theory) be willing to spend to obtain perfect information on an currently uncertain input parameter in order to assess the cost-effectiveness of an intervention with more certainty. Parameters with the highest EVPPI values contribute the most to the uncertainty associated with the optimal strategy for a given country at a given WTP value. EVPPIs were obtained for each country for a range of WTP values for all uncertain input parameters using the one-level method described in Strong et al.[66]

2.3.5 Identifying minimum conditions for vaccination to be optimal with threshold analysis

We performed a threshold analysis for the input parameters that caused the most uncertainty associated with the optimal strategy (i.e. the parameters with the highest EVPPI). We divided the input parameter range into 10 quantiles, with each quantile containing 200 samples. We then obtained the optimal strategy for each category: this is the strategy with highest average NB for that category, when averaged over the uncertainty of all other input parameters (similar to the approach described in Strong et al[66] for EVPPI). This was repeated for all countries and for a range of WTP values. We

then identified the minimum parameter value for which any vaccination strategy became optimal for each country and WTP value.

3 Results

3.1 Predicted disease and economic burden in the absence of vaccination

Appendix Table 18: Median [95% credible interval] discounted burden, costs and DALYs in the absence of vaccination, based on a 10-year time horizon. Costs are presented in USD2016. Output is discounted at a rate of 3% per year.

Country	Cases (thousands)	Deaths	DALYs (thousands)	Treatment Costs (thousands)
**Afghanistan**	2,019 (295-3,779)	6,958 (261-140,648)	178 (10-3,401)	4,916 (499-37,333)
**Bangladesh**	12,374 (8,370-16,306)	21,136 (296-839,056)	621 (36-21,889)	40,584 (8,541-358,576)
**Benin**	1,081 (219-1,952)	3,651 (165-83,879)	94 (6-2,016)	4,603 (547-35,637)
**Burkina Faso**	856 (700-1,034)	3,459 (211-57,991)	86 (8-1,353)	3,519 (891-24,137)
**Burundi**	1,114 (162-2,082)	3,807 (144-83,181)	95 (6-1,965)	2,526 (227-23,086)
**Cambodia**	710 (503-933)	2,786 (187-49,728)	75 (7-1,273)	3,465 (819-24,149)
**Cameroon**	490 (399-592)	1,965 (117-32,213)	48 (5-735)	3,027 (744-21,202)
**Central African Republic**	220 (54-399)	801 (38-15,697)	18 (1-337)	744 (112-5,510)
**Chad**	759 (334-1,217)	2,866 (157-50,560)	67 (6-1,115)	3,391 (694-26,500)
**Comoros**	5 (4-6)	20 (1-348)	0.514 (0.048-8)	24 (6-149)
**Dem Republic of Congo**	6,198 (1,045-12,273)	3.1.1.1.1.1 21,030 (704-456,817)	3.1.1.1.1.2 519 (28-10,719)	3.1.1.1.1.3 14,962 (1,654-128,470)
**Cote dIvoire**	805 (320-1,286)	2,738 (153-41,698)	70 (6-999)	4,362 (859-32,951)
**Djibouti**	7 (6-9)	28 (2-474)	0.724 (0.071-12)	51 (12-341)
**Eritrea**	162 (69-263)	618 (37-11,045)	17 (1-277)	397 (70-3,147)
**Ethiopia**	3,359 (1,247-5,617)	36,332 (1,705-646,845)	922 (55-15,840)	9,818 (1,853-69,084)
**Gambia**	30 (22-40)	122 (8-2,048)	3 (0.299-49)	145 (35-1,023)
**Ghana**	540 (466-630)	2,159 (140-36,577)	53 (5-829)	2,706 (672-18,032)
**Guinea**	481 (203-781)	1,765 (110-31,410)	45 (4-767)	1,548 (299-12,376)

**Guinea Bissau**	28 (6-50)	99 (5-2,016)	2 (0.153-47)	102 (13-831)
**Haiti**	105 (11-215)	371 (11-7,711)	8 (0.331-162)	430 (40-3,888)
**India**	36,341 (11,308-61,161)	284,698 (47,982-1,328,085)	6,990 (1,265-31,726)	464,094 (120,896-1,392,585)
**Kenya**	909 (783-1,040)	5,646 (353-66,389)	145 (13-1,634)	5,372 (1,253-31,124)
**DPR Korea**	577 (45-1,113)	1,947 (58-41,160)	47 (2-947)	2,101 (146-18,822)
**Kyrgyz Republic**	6 (0.368-11)	18 (1-432)	0.485 (0.019-11)	26 (2-208)
**Lao PDR**	367 (216-521)	484 (13-11,711)	14 (1-286)	1,838 (404-13,594)
**Lesotho**	5 (0.760-9)	17 (1-386)	0.395 (0.022-8)	26 (3-225)
**Liberia**	343 (87-627)	1,237 (60-25,676)	30 (2-592)	918 (124-8,193)
**Madagascar**	1,676 (328-2,992)	5,546 (303-113,780)	145 (10-2,747)	4,522 (600-38,959)
**Malawi**	220 (150-295)	863 (55-14,587)	22 (2-350)	800 (182-5,241)
**Mali**	491 (309-700)	1,951 (117-31,305)	48 (4-730)	1,936 (433-14,303)
**Mauritania**	76 (64-88)	305 (20-5,105)	8 (0.722-121)	408 (107-2,889)
**Mozambique**	626 (454-827)	2,514 (152-41,700)	60 (6-939)	2,182 (481-14,570)
**Myanmar**	1,236 (977-1,489)	4,893 (302-78,799)	125 (12-1,925)	5,211 (1,327-35,602)
**Nepal**	745 (320-1,183)	2,823 (167-54,837)	76 (7-1,365)	2,589 (465-19,883)
**Nicaragua**	41 (4-84)	147 (4-3,226)	4 (0.133-80)	328 (24-2,989)
**Niger**	572 (328-871)	2,194 (135-40,290)	57 (5-954)	1,683 (331-12,719)
**Nigeria**	7,436 (3,783-11,592)	104,732 (7,620-1,265,211)	2,279 (191-27,019)	48,538 (9,230-354,334)
**Pakistan**	3,695 (2,216-5,290)	5,657 (557-51,179)	162 (24-1,328)	3,215 (792-10,866)
**Papua New Guinea**	1,336 (316-1,464)	4,822 (258-80,188)	125 (10-1,944)	10,962 (1,947-76,479)
**Rwanda**	251 (121-394)	940 (58-18,281)	24 (2-430)	946 (204-7,113)
**Sao Tome e Principe**	1 (0.626-2)	5 (0-90)	0.137 (0.011-2)	7 (1-52)
**Senegal**	213 (163-289)	657 (39-7,747)	17 (2-187)	1,175 (290-8,014)
**Sierra Leone**	354 (135-575)	1,308 (72-24,237)	35 (3-613)	1,159 (226-8,976)
**Solomon Islands**	43 (10-77)	157 (6-3,038)	4 (0.247-67)	436 (63-3,523)
**Somalia**	378 (221-605)	1,492 (87-25,899)	38 (4-625)	1,155 (251-8,425)

**Republic of Sudan**	582 (211-967)	2,113 (124-39,604)	52 (4-893)	3,600 (686-29,594)
**South Sudan**	233 (149-330)	912 (55-15,856)	22 (2-351)	1,557 (357-11,075)
**Tajikistan**	10 (0.954-22)	36 (1-798)	0.949 (0.037-20)	46 (3-404)
**Tanzania**	1,718 (683-2,881)	6,626 (387-128,206)	178 (15-3,167)	4,493 (890-29,353)
**Togo**	142 (114-172)	555 (36-9,562)	14 (1-216)	493 (116-3,469)
**Uganda**	467 (381-564)	1,858 (120-32,065)	46 (4-742)	1,799 (416-11,695)
**Yemen**	6,448 (529-7,785)	19,309 (589-367,311)	519 (22-9,066)	39,632 (2,820-297,075)
**Zambia**	379 (216-584)	1,476 (89-26,262)	37 (3-614)	1,781 (390-12,812)
**Zimbabwe**	205 (14-426)	1,059 (34-19,738)	26 (0.937-459)	646 (35-5,811)

3.2 Predicted impact of vaccination

Appendix Table 19: Median [95% credible interval] discounted avoided burden, incremental costs and DALYs averted for routine vaccination against typhoid fever compared to no vaccination, based on a 10-year time horizon. Costs are presented in USD2016. Output is discounted at a rate of 3% per year.

Country	Cases Averted (thousands)	Deaths Averted	DALYs Averted (thousands)	Averted Treatment Costs (thousands)	Cost of Vaccination (thousands)	Net Costs (thousands), i.e. cost vaccination - averted treatment costs
**Afghanistan**	577 (85-1,303)	2,034 (67-41,931)	53 (3-988)	1,425 (129-12,120)	15,450 (6,199-36,600)	13,154 (306-34,665)
**Bangladesh**	4,007 (1,828-7,162)	6,961 (94-281,588)	200 (10-7,385)	13,215 (2,280-118,182)	71,680 (44,831-134,903)	54,410 (-51,562.329-117,439)
**Benin**	368 (77-848)	1,293 (55-32,007)	33 (2-784)	1,648 (188-13,952)	5,544 (3,546-8,601)	3,700 (-8,584.721-7,503)
**Burkina Faso**	316 (160-492)	1,224 (70-21,329)	31 (3-492)	1,281 (287-9,834)	9,227 (4,129-21,252)	7,387 (-956.736-19,380)
**Burundi**	401 (56-947)	1,395 (49-32,872)	35 (2-778)	941 (83-9,428)	5,686 (2,538-13,109)	4,334 (-3,226.733-11,963)
**Cambodia**	213 (98-378)	857 (49-16,323)	23 (2-408)	1,030 (213-8,157)	4,211 (3,040-7,242)	3,075 (-3,482.531-6,343)
**Cameroon**	184 (94-280)	701 (40-12,242)	17 (1-285)	1,145 (235-8,230)	13,519 (7,109-28,380)	11,835 (3,014-26,667)
**Central African Republic**	51 (12-126)	194 (8-4,151)	5 (0.303-88)	176 (24-1,501)	1,637 (731-3,763)	1,374 (-75.794-3,490)
**Chad**	210 (73-435)	797 (39-15,250)	19 (1-342)	951 (173-8,234)	5,390 (2,407-12,408)	4,109 (-2,917.185-11,306)
**Comoros**	2 (0.885-3)	7 (0-133)	0.184 (0.015-3)	8 (2-58)	419 (187-964)	405 (174-945)
**Dem Republic of Congo**	1,832 (286-4,687)	6,477 (208-150,142)	3.2.1.1.1.1 163 (8-3,491)	3.2.1.1.1.2 4,450 (477-39,508)	35,561 (16,184-80,111)	29,164 (-6,703.023-73,623)
**Cote dIvoire**	288 (100-582)	1,010 (54-16,593)	26 (2-396)	1,622 (284-13,672)	16,130 (9,923-30,568)	13,887 (2,020-28,221)
**Djibouti**	2 (1-4)	9 (0-147)	0.231 (0.019-4)	15 (3-117)	401 (190-880)	375 (156-848)
**Eritrea**	54 (19-117)	217 (11-4,384)	6 (0.382-109)	138 (22-1,199)	2,717 (1,245-6,189)	2,487 (815-5,932)
**Ethiopia**	1,034 (339-2,151)	10,987 (495-212,224)	275 (16-5,052)	3,071 (531-23,255)	42,149 (19,145-95,204)	37,460 (9,147-91,013)
**Gambia**	11 (5-19)	43 (2-764)	1 (0.088-18)	52 (11-394)	1,034 (462-2,384)	947 (336-2,271)
**Ghana**	186 (91-301)	731 (40-12,701)	19 (2-305)	912 (190-6,787)	24,975 (18,522-33,685)	23,538 (15,540-32,445)
**Guinea**	126 (42-258)	482 (25-9,285)	12 (0.902-220)	412 (70-3,443)	4,591 (2,049-10,582)	3,954 (334-9,995)
**Guinea Bissau**	8 (2-21)	32 (1-764)	0.801 (0.048-18)	32 (4-281)	923 (412-2,122)	862 (325-2,053)
**Haiti**	14 (1-45)	50 (1-1,260)	1 (0.045-28)	59 (5-646)	3,019 (1,247-7,121)	2,901 (1,079-6,902)

**India**	8,670 (2,145-20,295)	68,333 (10,549-356,105)	1,701 (269-8,521)	113,766 (22,818-392,177)	711,528 (523,528-1,129,054)	584,510 (277,632-1,013,065)
**Kenya**	290 (141-464)	1,730 (102-22,519)	45 (4-573)	1,660 (350-10,471)	29,605 (17,714-57,114)	27,274 (13,419-55,093)
**DPR Korea**	125 (11-370)	462 (13-9,865)	12 (0.421-237)	488 (33-5,069)	5,403 (2,558-12,217)	4,572 (326-11,433)
**Kyrgyz Republic**	1 (0.084-4)	5 (0-112)	0.126 (0.004-3)	6 (0.339-64)	4,391 (1,707-10,383)	4,378 (1,689-10,376)
**Lao PDR**	103 (45-195)	134 (3-3,275)	4 (0.335-82)	541 (103-4,284)	2,722 (2,231-3,966)	2,144 (-1,606.392-3,556)
**Lesotho**	1 (0.226-4)	5 (0-128)	0.125 (0.006-3)	8 (0.794-78)	902 (488-1,865)	888 (473-1,844)
**Liberia**	96 (23-254)	368 (15-8,585)	9 (0.588-197)	270 (34-2,839)	2,229 (996-5,126)	1,802 (-748.250-4,814)
**Madagascar**	416 (76-1,005)	1,473 (69-33,385)	38 (2-801)	1,178 (146-10,635)	9,207 (4,115-21,160)	7,400 (-2,795.220-19,507)
**Malawi**	72 (34-130)	294 (16-5,174)	8 (0.616-121)	261 (54-1,840)	8,231 (3,681-18,902)	7,775 (3,143-18,289)
**Mali**	167 (72-303)	646 (37-12,256)	16 (1-285)	661 (128-4,946)	8,380 (3,741-19,311)	7,357 (1,599-18,271)
**Mauritania**	24 (12-37)	94 (5-1,647)	2 (0.202-39)	127 (29-959)	2,126 (1,120-4,454)	1,917 (768-4,251)
**Mozambique**	222 (104-377)	881 (48-15,198)	21 (2-347)	765 (151-5,452)	14,232 (6,354-32,817)	12,989 (4,559-31,260)
**Myanmar**	311 (141-580)	1,232 (68-21,874)	33 (2-556)	1,340 (275-10,043)	15,221 (9,214-29,368)	13,285 (4,173-27,523)
**Nepal**	218 (76-472)	869 (42-17,362)	23 (2-441)	781 (118-6,700)	8,779 (4,340-19,350)	7,628 (907-18,088)
**Nicaragua**	9 (0.905-30)	34 (1-817)	0.950 (0.030-21)	74 (5-818)	4,055 (2,525-7,516)	3,891 (2,313-7,371)
**Niger**	189 (80-370)	767 (40-13,307)	19 (1-323)	561 (100-4,333)	9,266 (4,140-21,301)	8,407 (2,498-20,371)
**Nigeria**	1,502 (544-3,180)	21,133 (1,408-269,814)	467 (36-5,975)	10,015 (1,636-81,825)	101,949 (68,766-177,489)	88,255 (19,494-162,489)
**Pakistan**	850 (369-1,607)	1,293 (118-12,786)	38 (5-322)	760 (164-2,905)	80,161 (45,758-157,679)	79,196 (44,709-156,907)
**Papua New Guinea**	389 (92-596)	1,396 (72-24,462)	37 (3-613)	3,263 (506-26,688)	2,865 (2,349-4,175)	-354.911 (-23,428.773-2,738)
**Rwanda**	85 (33-180)	328 (16-6,468)	8 (0.682-155)	324 (61-2,782)	3,042 (1,891-7,139)	2,569 (338-6,869)
**Sao Tome e Principe**	0.443 (0.152-0.989)	2 (0-33)	0.046 (0.003-0.814)	2 (0.398-18)	164 (109-291)	160 (105-285)
**Senegal**	65 (29-125)	196 (13-2,647)	5 (0.487-65)	364 (74-2,728)	8,470 (4,210-18,522)	7,865 (3,404-17,848)
**Sierra Leone**	122 (41-258)	460 (24-9,284)	12 (0.941-224)	409 (70-3,354)	3,283 (1,466-7,570)	2,712 (-398.188-6,949)
**Solomon Islands**	17 (4-36)	62 (2-1,273)	1 (0.090-28)	173 (23-1,461)	391 (320-572)	224 (-1,041.300-469)
**Somalia**	74 (30-169)	298 (15-5,481)	8 (0.596-133)	227 (39-1,805)	4,255 (1,705-10,120)	3,890 (1,067-9,686)
**Republic of Sudan**	208 (69-450)	790 (40-16,133)	19 (1-373)	1,378 (239-12,000)	36,135 (20,900-70,801)	33,776 (17,029-68,336)
**South Sudan**	29 (13-56)	114 (6-2,201)	3 (0.215-49)	202 (39-1,547)	2,971 (1,372-6,618)	2,642 (805-6,316)
**Tajikistan**	3 (0.264-8)	11 (0-248)	0.278 (0.010-6)	13 (0.887-141)	9,234 (3,600-21,823)	9,209 (3,553-21,727)
**Tanzania**	579 (186-1,279)	2,316 (111-50,528)	62 (5-1,214)	1,542 (272-11,171)	29,933 (14,875-65,442)	27,568 (11,046-62,396)
**Togo**	51 (25-80)	198 (11-3,394)	5 (0.437-80)	174 (37-1,337)	3,717 (1,659-8,568)	3,430 (1,270-8,261)
**Uganda**	171 (88-269)	676 (37-11,993)	17 (1-274)	644 (137-4,834)	19,837 (8,856-45,714)	18,759 (7,591-44,336)
**Yemen**	2,000 (193-3,412)	6,812 (205-134,162)	182 (8-3,226)	13,974 (1,047-111,577)	16,840 (7,970-36,929)	2,893 (-91,728.755-27,580)

**Zambia**	128 (52-262)	529 (26-9,693)	14 (1-229)	617 (119-4,790)	15,840 (11,828-20,963)	15,002 (9,888-20,239)
**Zimbabwe**	68 (4-181)	366 (11-7,120)	9 (0.310-166)	217 (12-2,071)	7,937 (3,544-18,301)	7,560 (2,943-17,686)

Appendix Table 20: Median [95% credible interval] discounted avoided burden, incremental costs and DALYs averted for routine vaccination against typhoid fever with a catch-up campaign up to 5 years of age compared to no vaccination, based on a 10-year time horizon. Costs are presented in USD2016. Output is discounted at a rate of 3% per year.

Country	Cases Averted (thousands)	Deaths Averted	DALYs Averted (thousands)	Averted Treatment Costs (thousands)	Cost of Vaccination (thousand)	Net Costs (thousands), i.e. cost vaccination - averted treatment costs
**Afghanistan**	897 (129-1,939)	3,111 (101-64,453)	83 (4-1,573)	2,174 (202-18,468)	16,183 (6,920-37,357)	12,981 (-4,251.971-34,560)
**Bangladesh**	5,665 (2,662-9,488)	9,708 (134-398,986)	285 (15-10,523)	18,560 (3,276-169,342)	74,115 (47,391-137,263)	51,289 (-99,112.618-114,836)
**Benin**	521 (109-1,150)	1,841 (79-45,219)	47 (3-1,119)	2,303 (271-19,254)	5,787 (3,786-8,845)	3,361 (-13,904.209-7,477)
**Burkina Faso**	457 (240-674)	1,781 (99-29,743)	46 (4-700)	1,863 (417-13,858)	9,658 (4,538-21,652)	7,198 (-4,197.843-19,382)
**Burundi**	578 (81-1,302)	1,993 (74-46,166)	50 (3-1,111)	1,326 (120-13,171)	5,936 (2,784-13,357)	4,137 (-6,835.939-11,654)
**Cambodia**	312 (152-514)	1,229 (71-23,535)	34 (3-613)	1,479 (307-11,795)	4,454 (3,288-7,467)	2,878 (-6,944.307-6,229)
**Cameroon**	260 (134-376)	997 (54-16,964)	25 (2-404)	1,583 (337-11,616)	14,051 (7,645-28,909)	11,808 (763-26,693)
**Central African Republic**	89 (21-205)	340 (15-6,949)	8 (0.529-152)	308 (43-2,501)	1,745 (840-3,873)	1,330 (-1,037.618-3,474)
**Chad**	334 (116-661)	1,285 (66-24,098)	30 (2-547)	1,530 (280-13,130)	5,703 (2,718-12,719)	3,732 (-6,684.331-11,232)
**Comoros**	3 (1-4)	10 (1-190)	0.272 (0.023-5)	12 (3-86)	436 (205-981)	415 (183-951)
**Dem Republic of Congo**	2,659 (423-6,458)	9,301 (302-212,288)	238 (11-5,018)	6,528 (709-56,857)	36,940 (17,613-81,451)	28,078 (-20,018.771-73,474)
**Cote dIvoire**	404 (139-776)	1,414 (77-22,926)	37 (3-554)	2,286 (397-19,074)	16,641 (10,439-31,077)	13,680 (-2,550.145-28,165)
**Djibouti**	3 (2-5)	13 (1-210)	0.333 (0.027-5)	23 (5-169)	415 (204-894)	379 (151-852)
**Eritrea**	81 (29-162)	316 (16-6,227)	9 (0.594-160)	203 (34-1,785)	2,834 (1,360-6,305)	2,517 (634-5,979)
**Ethiopia**	1,441 (469-2,885)	15,387 (659-287,965)	395 (23-6,986)	4,235 (760-33,135)	43,935 (20,943-97,084)	37,751 (5,699-92,112)
**Gambia**	16 (8-26)	63 (4-1,079)	2 (0.134-26)	76 (17-559)	1,079 (505-2,428)	961 (288-2,291)
**Ghana**	278 (141-414)	1,081 (61-19,204)	28 (2-452)	1,347 (298-9,983)	25,584 (19,142-34,301)	23,592 (14,007-32,695)
**Guinea**	211 (73-417)	800 (43-14,697)	21 (2-354)	683 (116-5,715)	4,872 (2,330-10,859)	3,890 (-1,312.159-10,054)
**Guinea Bissau**	13 (3-30)	49 (2-1,129)	1 (0.074-26)	48 (6-411)	964 (453-2,164)	881 (288-2,073)
**Haiti**	26 (3-83)	96 (3-2,367)	2 (0.088-53)	114 (9-1,215)	3,185 (1,406-7,288)	2,956 (1,044-6,967)
**India**	12,153 (3,173-26,130)	95,344 (15,314-474,149)	2,413 (394-11,387)	156,980 (32,828-512,374)	842,801 (654,654-1,268,809)	674,748 (297,107-1,106,380)

**Kenya**	449 (224-659)	2,669 (162-35,065)	70 (6-869)	2,558 (530-15,973)	30,671 (18,773-58,184)	26,996 (10,446-55,499)
**DPR Korea**	189 (15-520)	694 (20-14,926)	18 (0.656-367)	737 (50-7,064)	5,652 (2,816-12,460)	4,500 (-1,497.863-11,383)
**Kyrgyz Republic**	2 (0.129-6)	7 (0-168)	0.198 (0.006-4)	10 (0.526-94)	4,484 (1,802-10,477)	4,463 (1,782-10,468)
**Lao PDR**	155 (70-277)	200 (5-4,718)	6 (0.511-119)	800 (155-6,352)	2,849 (2,361-4,101)	2,040 (-3,394.828-3,514)
**Lesotho**	2 (0.334-5)	8 (0-181)	0.180 (0.009-4)	11 (1-110)	935 (521-1,898)	915 (498-1,869)
**Liberia**	151 (37-369)	567 (26-13,241)	15 (0.910-312)	420 (54-4,259)	2,329 (1,095-5,230)	1,747 (-1,995.461-4,810)
**Madagascar**	708 (131-1,612)	2,424 (118-54,334)	66 (4-1,345)	1,964 (240-17,777)	9,747 (4,650-21,715)	7,095 (-9,508.427-19,383)
**Malawi**	109 (54-183)	442 (25-7,760)	11 (0.955-184)	384 (79-2,655)	8,614 (4,056-19,302)	7,937 (3,181-18,440)
**Mali**	248 (111-426)	949 (54-17,548)	24 (2-415)	978 (190-7,126)	8,774 (4,129-19,700)	7,327 (168-18,243)
**Mauritania**	37 (19-54)	146 (8-2,507)	4 (0.318-61)	195 (47-1,468)	2,220 (1,214-4,548)	1,925 (492-4,266)
**Mozambique**	325 (159-520)	1,302 (71-22,412)	32 (3-511)	1,130 (224-7,930)	14,859 (6,968-33,431)	13,017 (3,844-31,699)
**Myanmar**	466 (219-780)	1,845 (102-32,433)	49 (4-821)	1,952 (417-14,018)	15,767 (9,789-29,895)	13,129 (616-27,739)
**Nepal**	321 (113-647)	1,269 (63-25,864)	35 (3-667)	1,129 (186-9,691)	9,201 (4,772-19,798)	7,655 (-1,133.270-18,148)
**Nicaragua**	14 (1-42)	54 (1-1,247)	1 (0.048-33)	117 (8-1,217)	4,153 (2,626-7,612)	3,921 (2,222-7,409)
**Niger**	283 (126-523)	1,138 (61-20,370)	30 (2-502)	838 (152-6,529)	9,707 (4,575-21,763)	8,479 (1,659-20,694)
**Nigeria**	2,573 (1,036-5,116)	36,708 (2,483-439,819)	825 (64-9,715)	17,104 (2,959-134,583)	106,576 (73,727-183,832)	85,979 (-26,728.543-162,132)
**Pakistan**	1,272 (580-2,248)	1,915 (184-19,080)	57 (8-482)	1,114 (253-4,166)	82,698 (48,662-160,095)	81,464 (47,052-159,065)
**Papua New Guinea**	563 (136-805)	2,033 (104-36,998)	54 (4-907)	4,652 (766-37,658)	2,994 (2,475-4,302)	-1,581.844 (-34,582.312-2,565)
**Rwanda**	124 (51-253)	481 (24-9,508)	13 (1-230)	483 (93-3,888)	3,302 (2,150-7,389)	2,665 (-619.480-6,943)
**Sao Tome e Principe**	0.672 (0.238-1)	3 (0-48)	0.069 (0.005-1)	3 (0.602-28)	169 (113-295)	162 (106-288)
**Senegal**	101 (49-179)	308 (20-4,035)	8 (0.759-98)	557 (120-4,084)	8,807 (4,542-18,849)	7,941 (3,101-17,858)
**Sierra Leone**	179 (61-358)	678 (36-13,043)	18 (1-333)	596 (108-4,746)	3,427 (1,607-7,712)	2,623 (-1,491.872-6,983)
**Solomon Islands**	23 (5-49)	88 (3-1,760)	2 (0.128-38)	236 (32-1,963)	406 (335-586)	179 (-1,527.892-461)
**Somalia**	141 (61-292)	575 (29-9,876)	15 (1-256)	428 (80-3,417)	4,500 (1,941-10,371)	3,825 (333-9,744)
**Republic of Sudan**	293 (98-598)	1,082 (58-21,862)	27 (2-503)	1,881 (331-17,233)	37,067 (21,824-71,738)	33,974 (14,919-69,163)

**South Sudan**	75 (33-134)	291 (15-5,656)	7 (0.567-126)	511 (100-3,954)	3,251 (1,647-6,906)	2,540 (-713.513-6,282)
**Tajikistan**	4 (0.411-13)	16 (0-387)	0.431 (0.016-10)	20 (1-218)	9,431 (3,799-22,021)	9,384 (3,749-21,886)
**Tanzania**	857 (292-1,774)	3,440 (172-71,871)	93 (7-1,788)	2,277 (402-15,829)	31,130 (16,051-66,625)	28,003 (9,702-63,158)
**Togo**	75 (39-111)	295 (17-4,985)	8 (0.644-118)	250 (54-1,887)	3,880 (1,820-8,730)	3,471 (1,151-8,337)
**Uganda**	248 (130-368)	969 (54-17,238)	25 (2-405)	937 (202-6,812)	20,759 (9,787-46,580)	19,225 (7,624-44,750)
**Yemen**	2,790 (262-4,507)	9,315 (268-184,851)	253 (11-4,625)	19,051 (1,431-146,847)	17,455 (8,580-37,554)	-1,058.212 (-128,857.713-26,592)
**Zambia**	189 (83-366)	771 (39-13,876)	20 (2-326)	902 (183-7,004)	16,201 (12,187-21,336)	14,989 (8,319-20,300)
**Zimbabwe**	101 (7-254)	538 (16-10,507)	13 (0.481-246)	323 (18-2,980)	8,287 (3,886-18,644)	7,667 (2,925-17,980)

Appendix Table 21: Median [95% credible interval] discounted avoided burden, incremental costs and DALYs averted for routine vaccination against typhoid fever with a catch-up campaign up to 15 years of age compared to no vaccination, based on a 10-year time horizon. Costs are presented in USD2016. Output is discounted at a rate of 3% per year.

Country	Cases Averted (thousands)	Deaths Averted	DALYs Averted (thousands)	Averted Treatment Costs (thousands)	Cost of Vaccination (thousand)	Net Costs (thousands), i.e. cost vaccination - averted treatment costs
**Afghanistan**	1,209 (175-2,633)	4,237 (141-88,057)	112 (6-2,156)	2,969 (275-25,405)	17,683 (8,435-38,897)	13,668 (-8,024.880-35,634)
**Bangladesh**	7,511 (3,721-11,838)	12,752 (175-528,811)	374 (21-14,020)	24,575 (4,406-222,553)	79,931 (53,267-143,117)	51,731 (-151,474.341-116,454)
**Benin**	670 (138-1,440)	2,394 (100-56,738)	62 (4-1,398)	2,992 (364-24,530)	6,303 (4,292-9,375)	3,276 (-19,099.011-7,701)
**Burkina Faso**	611 (326-836)	2,379 (135-39,350)	61 (5-939)	2,468 (568-17,647)	10,525 (5,416-22,451)	7,366 (-7,018.922-19,785)
**Burundi**	757 (110-1,620)	2,575 (97-59,835)	66 (3-1,403)	1,732 (153-16,461)	6,455 (3,317-13,850)	4,288 (-9,887.410-12,026)
**Cambodia**	424 (215-658)	1,659 (103-31,502)	45 (4-813)	2,066 (433-16,106)	5,013 (3,849-8,087)	2,935 (-9,793.962-6,438)
**Cameroon**	336 (169-464)	1,288 (70-21,719)	33 (3-509)	2,052 (430-14,906)	15,151 (8,744-30,021)	12,416 (-691.901-27,470)
**Central African Republic**	133 (32-288)	495 (23-10,085)	12 (0.770-220)	455 (64-3,648)	1,973 (1,071-4,089)	1,403 (-1,796.591-3,598)
**Chad**	470 (171-893)	1,798 (91-33,546)	43 (3-740)	2,174 (398-17,838)	6,356 (3,379-13,337)	3,818 (-10,494.677-11,440)
**Comoros**	4 (2-5)	14 (1-258)	0.368 (0.032-6)	17 (4-113)	473 (242-1,016)	445 (208-983)
**Dem Republic of Congo**	3,701 (627-8,462)	12,935 (425-290,127)	328 (15-6,811)	9,060 (979-77,093)	39,955 (20,540-84,284)	28,068 (-37,818.483-74,427)
**Cote dIvoire**	513 (177-968)	1,820 (101-28,474)	47 (4-698)	2,887 (518-23,770)	17,681 (11,503-32,077)	14,126 (-5,251.834-28,970)
**Djibouti**	4 (2-6)	17 (1-282)	0.441 (0.035-7)	30 (6-220)	447 (236-927)	402 (151-877)
**Eritrea**	110 (41-208)	431 (23-8,149)	12 (0.843-210)	275 (46-2,259)	3,077 (1,609-6,535)	2,667 (398-6,128)
**Ethiopia**	1,870 (609-3,695)	20,073 (854-372,091)	519 (28-9,216)	5,512 (977-42,830)	47,678 (24,791-100,964)	39,989 (673-93,681)
**Gambia**	21 (11-33)	84 (5-1,432)	2 (0.189-35)	101 (23-730)	1,171 (600-2,516)	1,023 (254-2,343)
**Ghana**	382 (210-521)	1,462 (85-26,021)	38 (3-612)	1,837 (412-13,306)	26,853 (20,436-35,594)	24,319 (12,386-33,514)
**Guinea**	300 (109-573)	1,131 (65-20,645)	29 (2-499)	973 (170-8,060)	5,455 (2,923-11,415)	4,121 (-2,778.326-10,328)
**Guinea Bissau**	18 (4-39)	67 (3-1,502)	2 (0.101-36)	68 (9-572)	1,049 (541-2,246)	944 (278-2,110)
**Haiti**	48 (5-131)	174 (5-4,104)	4 (0.156-88)	203 (17-2,041)	3,564 (1,793-7,648)	3,225 (950-7,267)
**India**	16,949 (4,741-34,122)	132,252 (21,626-632,682)	3,326 (568-15,583)	217,987 (48,914-694,892)	1,170,403 (979,505-1,583,218)	949,238 (458,882-1,395,297)

**Kenya**	625 (339-842)	3,713 (231-45,378)	99 (8-1,183)	3,578 (731-21,788)	32,817 (20,954-60,419)	28,059 (7,051-56,915)
**DPR Korea**	281 (23-693)	1,006 (28-22,493)	26 (1-534)	1,061 (74-9,901)	6,255 (3,420-13,026)	4,755 (-3,514.252-11,815)
**Kyrgyz Republic**	3 (0.207-8)	10 (0-258)	0.287 (0.010-6)	14 (0.804-129)	4,703 (2,013-10,698)	4,674 (1,989-10,676)
**Lao PDR**	211 (96-359)	270 (7-6,555)	8 (0.719-165)	1,080 (215-8,502)	3,156 (2,645-4,395)	2,074 (-5,298.447-3,652)
**Lesotho**	3 (0.447-6)	10 (0-242)	0.246 (0.012-5)	15 (2-142)	1,012 (598-1,975)	984 (561-1,947)
**Liberia**	220 (57-490)	819 (37-18,245)	21 (1-432)	601 (81-5,926)	2,539 (1,310-5,429)	1,758 (-3,403.921-4,880)
**Madagascar**	1,026 (201-2,199)	3,580 (178-78,604)	94 (6-1,972)	2,873 (359-25,544)	10,874 (5,794-22,784)	7,303 (-15,240.558-19,571)
**Malawi**	149 (79-235)	603 (35-10,154)	16 (1-245)	535 (119-3,578)	9,413 (4,873-20,077)	8,559 (3,465-19,033)
**Mali**	335 (161-546)	1,303 (76-22,758)	33 (3-537)	1,319 (263-9,596)	9,594 (4,965-20,476)	7,772 (-910.480-18,769)
**Mauritania**	52 (28-70)	201 (12-3,386)	5 (0.438-83)	270 (67-1,989)	2,409 (1,406-4,743)	2,025 (274-4,343)
**Mozambique**	438 (233-659)	1,741 (100-29,535)	43 (4-672)	1,536 (316-10,430)	16,157 (8,303-34,664)	13,855 (3,164-32,174)
**Myanmar**	657 (325-987)	2,523 (147-44,570)	69 (6-1,121)	2,738 (620-19,345)	17,077 (11,109-31,220)	13,499 (-3,327.577-28,391)
**Nepal**	438 (155-828)	1,703 (86-34,054)	47 (4-886)	1,500 (257-12,873)	10,212 (5,791-20,757)	8,151 (-3,063.820-18,846)
**Nicaragua**	23 (2-58)	83 (2-1,828)	2 (0.076-47)	180 (12-1,737)	4,389 (2,860-7,844)	4,075 (2,103-7,584)
**Niger**	384 (175-673)	1,526 (81-27,720)	40 (3-666)	1,136 (210-8,851)	10,632 (5,520-22,641)	9,049 (823-21,270)
**Nigeria**	3,995 (1,716-7,435)	57,402 (4,103-670,962)	1,268 (105-14,856)	26,349 (4,798-196,841)	116,305 (82,797-192,306)	86,284 (-87,807.736-166,206)
**Pakistan**	1,744 (800-2,976)	2,690 (266-25,327)	80 (11-655)	1,523 (362-5,668)	89,218 (54,903-166,908)	87,122 (52,367-164,258)
**Papua New Guinea**	745 (178-1,011)	2,631 (133-48,237)	71 (5-1,190)	6,156 (991-47,522)	3,288 (2,754-4,583)	-2,762.964 (-44,408.848-2,547)
**Rwanda**	169 (72-319)	651 (34-12,576)	17 (1-301)	656 (130-5,095)	3,843 (2,691-7,919)	3,060 (-1,311.022-7,328)
**Sao Tome e Principe**	0.949 (0.357-2)	4 (0-64)	0.098 (0.007-2)	5 (0.884-37)	178 (123-305)	170 (111-296)
**Senegal**	144 (79-232)	438 (28-5,647)	12 (1-136)	791 (183-5,789)	9,501 (5,264-19,516)	8,351 (2,822-18,275)
**Sierra Leone**	241 (87-456)	899 (48-17,862)	24 (2-443)	805 (151-6,181)	3,727 (1,915-7,996)	2,713 (-2,436.707-7,194)
**Solomon Islands**	29 (7-60)	109 (4-2,177)	3 (0.159-47)	301 (40-2,404)	435 (363-616)	144 (-2,005.576-467)
**Somalia**	223 (107-431)	903 (48-16,245)	24 (2-392)	687 (132-5,348)	5,001 (2,454-10,847)	4,024 (-671.965-10,037)
**Republic of Sudan**	373 (126-751)	1,373 (73-27,625)	34 (2-647)	2,413 (410-21,747)	38,981 (23,709-73,569)	35,292 (13,610-70,497)

**South Sudan**	129 (60-219)	503 (27-9,368)	12 (0.998-214)	867 (179-6,821)	3,819 (2,236-7,459)	2,737 (-2,796.753-6,568)
**Tajikistan**	6 (0.626-16)	23 (1-546)	0.633 (0.022-14)	29 (2-291)	9,840 (4,197-22,435)	9,770 (4,155-22,258)
**Tanzania**	1,164 (416-2,263)	4,590 (236-92,989)	126 (9-2,337)	3,081 (548-21,888)	33,578 (18,600-68,983)	29,454 (8,983-64,313)
**Togo**	101 (55-138)	392 (23-6,788)	10 (0.837-159)	336 (74-2,560)	4,219 (2,168-9,052)	3,695 (1,131-8,512)
**Uganda**	330 (177-455)	1,303 (75-22,642)	33 (3-532)	1,233 (276-8,889)	22,667 (11,778-48,301)	20,806 (8,395-46,191)
**Yemen**	3,495 (313-5,574)	11,577 (353-233,733)	315 (13-5,970)	23,732 (1,699-184,762)	18,734 (9,839-38,817)	-4,063.996 (-160,213.522-26,612)
**Zambia**	258 (121-467)	1,038 (59-18,432)	27 (2-439)	1,233 (246-9,387)	16,960 (12,961-22,121)	15,372 (7,042-20,817)
**Zimbabwe**	139 (10-332)	753 (23-13,920)	18 (0.689-325)	450 (25-4,137)	9,012 (4,632-19,332)	8,253 (3,288-18,642)

3.3 Cost-effectiveness of vaccination

3.3.1 What makes vaccination optimal in some countries but not in others?

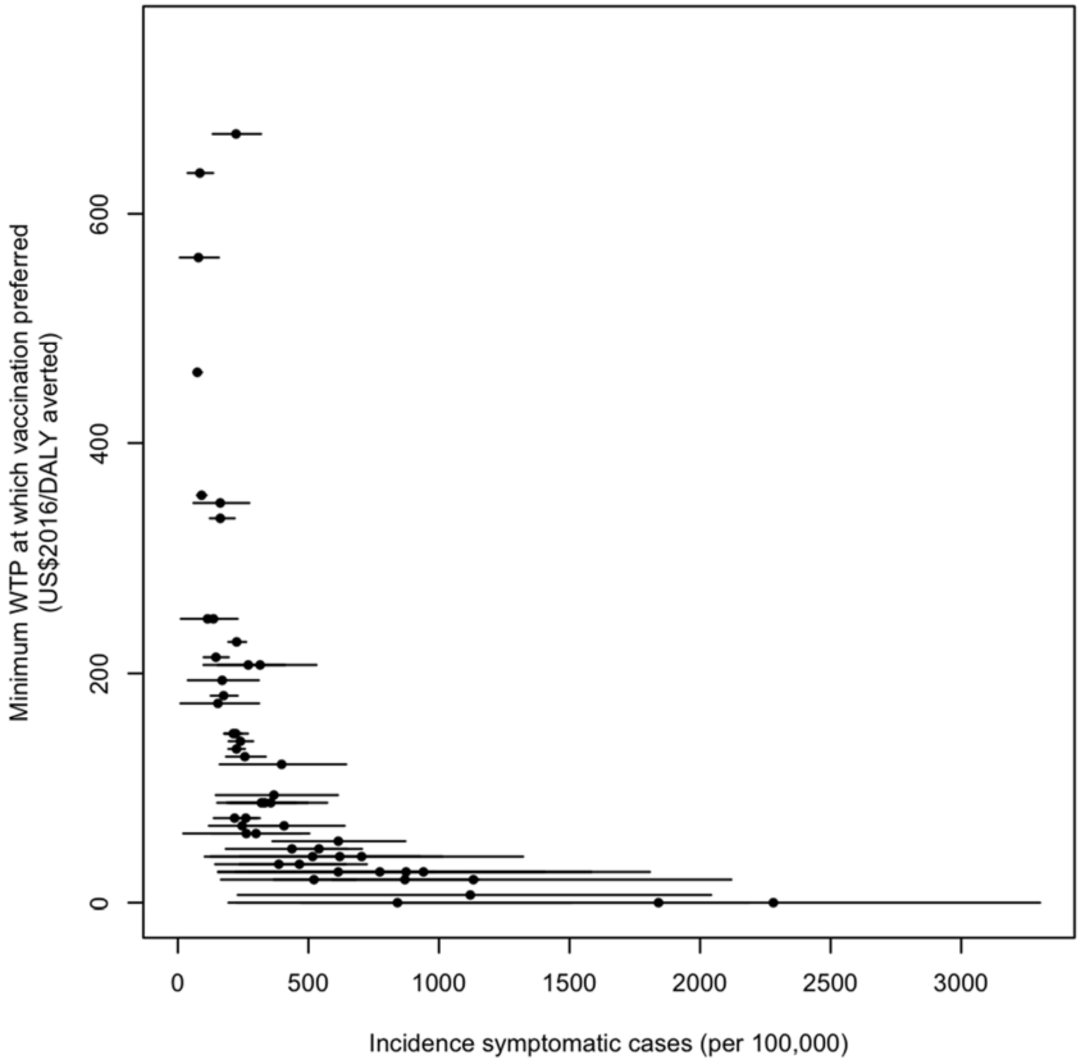

Appendix Figure 4: Relationship between typhoid fever incidence and the minimum willingness-to-pay value at which any vaccination strategy becomes optimal when compared to no vaccination. The annual incidence of symptomatic cases per 100,000 person-years (mean and 95% credible interval) used as an input in our health economic evaluation is plotted on the X-axis for 51 Gavi-eligible countries. The minimum WTP value at which vaccination becomes optimal when compared to no vaccination is plotted on the Y-axis for 51 Gavi-eligible countries. Lesotho, Kyrgyz Republic and Tajikistan are not shown on this plot as the minimum WTP value at which vaccination becomes optimal in these countries exceeds USD1200 per DALY averted.

3.3.2 What are the key drivers of uncertainty (EVPPI)?

Uncertainty around the probability of hospitalisation for typhoid fever contributes the most to uncertainty about the optimal strategy in all countries except for India and Pakistan, i.e. the typhoid hospitalisation rate has the highest EVPPI (black solid line in Appendix Figure 5). Appendix Figure 5 shows the EVPPI for Zimbabwe, Guinea Bissau and Myanmar; the EVPPI plots for all other countries (except for India and Pakistan) look similar to one of these three (https://ceatyphoid.uantwerpen.be/home/). Thus, there is the most value in obtaining more information on the probability of hospitalisation for typhoid fever in order to identify the optimal TCV strategy with more certainty. The WTP value at which the EVPPI values are

greatest corresponds to the WTP value at which the optimal strategy is most uncertain (e.g. the greatest uncertainty in the optimal strategy occurs at a WTP of $250 per DALY averted in Zimbabwe).

In Cambodia, Chad, Ethiopia, Kyrgyz Republic, Nigeria, Papua New Guinea and Tajikiskistan, the uncertainty around the probability of hospitalisation is estimated to contribute far more to decision uncertainty than any other uncertain parameter. In Benin, Burundi, Central African Republic, Democratic Republic of Congo, DPR Korea, Liberia, Madagascar, Solomon Islands, Yemen and Zimbabwe the uncertainty about typhoid incidence is the second-most important contributor to uncertainty about the optimal strategy. Uncertainty about both typhoid incidence and typhoid mortality within a hospital setting are the other key drivers of uncertainty for Afghanistan, Ivory Coast, Haiti and Lesotho. For all other 31 countries, additional evidence on the case fatality rate of typhoid among inpatients is the second-most valuable parameter (https://ceatyphoid.uantwerpen.be/home/).

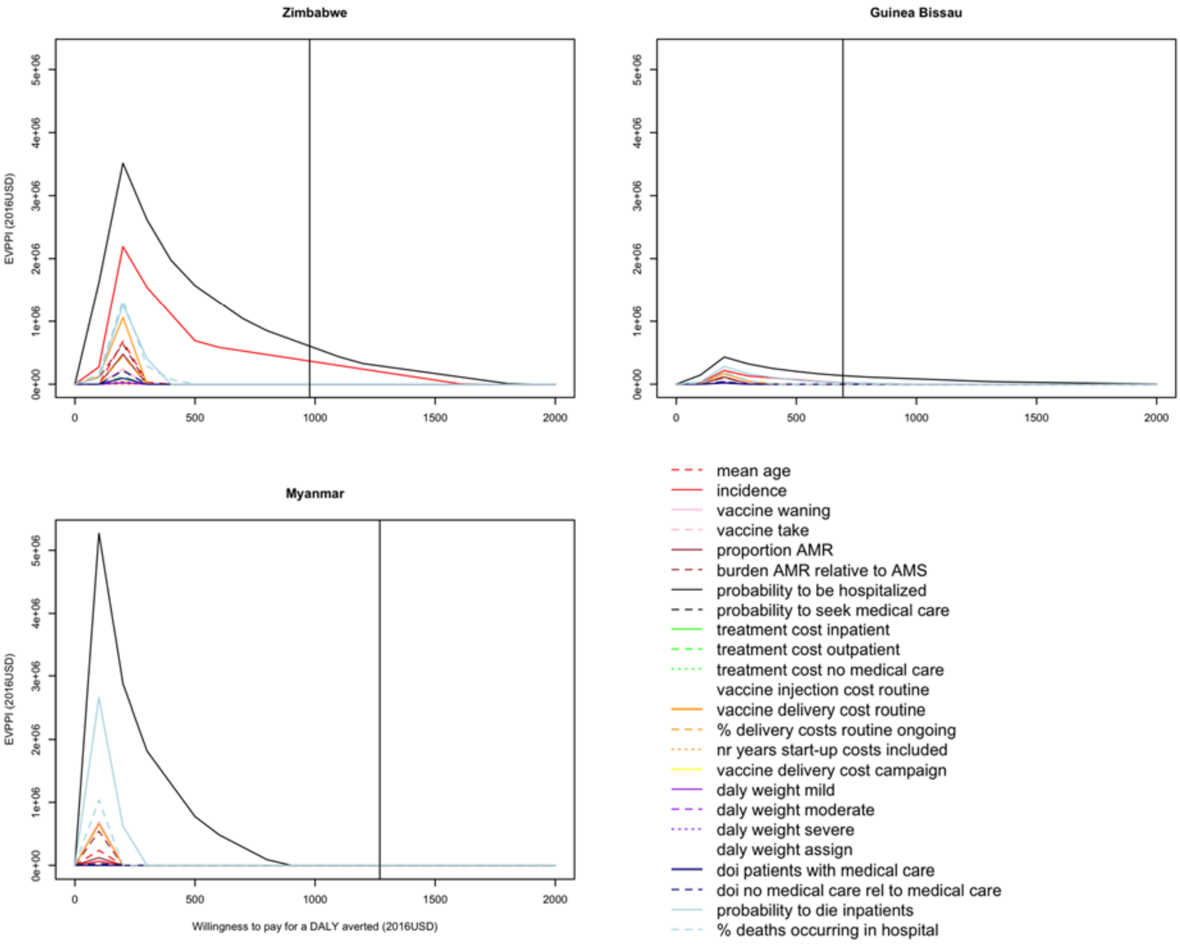

Appendix Figure 5: Expected value of partial perfect information for three representative countries. The expected value of partial perfect information (EVPPI) for each uncertain parameter is plotted on the Y-axis for a range of willingness-to-pay values (X-axis) for Zimbabwe, Guinea Bissau and Myanmar. Vertical black bars represent the country's GDP per capita. Results are based on 2,000 parameter samples. Costs are presented in US$2016. We assume a vaccine price of US$1·50 per dose, but exclude Gavi's contribution depending on each country's expected graduation from Gavi support. See also https://ceatyphoid.uantwerpen.be/home/.

For India, uncertainty around typhoid incidence contributes the most to uncertainty around the optimal strategy (red solid line in Appendix Figure 6, left panel). For Pakistan, uncertainty around typhoid mortality within a hospital setting contributes the most to uncertainty around the optimal strategy (light blue solid line in Appendix Figure 6, right panel).

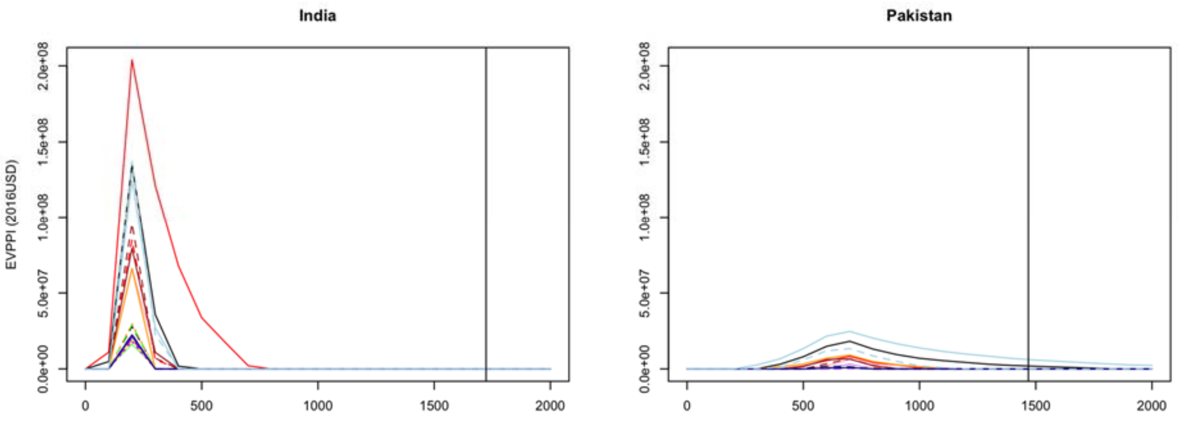

Appendix Figure 6: Expected value of partial perfect information for India and Pakistan. The expected value of partial perfect information (EVPPI) for each uncertain parameter is plotted on the Y-axis for a range of willingness-to-pay values (X-axis). See Appendix Figure 5 for legend of the coloured lines. Vertical black bars represent the country's GDP per capita. Results are based on 2,000 parameter samples. Costs are presented in US$2016. We assume a vaccine price of US$1·50 per dose, but exclude Gavi's contribution depending on each country's expected graduation from Gavi support. See also https://ceatyphoid.uantwerpen.be/home/.

3.3.3 Minimum conditions under which vaccination is optimal (threshold analysis)

Appendix Table 22 shows the minimum probability of hospitalisation for typhoid for which routine vaccination with a catch-up campaign up to age 15 years (RC15) becomes optimal, for the countries and WTP values for which the uncertainty around the typhoid hospitalisation rate influences the optimal strategy. For example, for Afghanistan, uncertainty around the typhoid hospitalisation rate influences the optimal strategy for WTP values of $100-400 per DALY averted. At a WTP of $100 per DALY averted, RC15 is optimal compared to no vaccination if the probability of hospitalisation for typhoid is at least 0.03. For higher WTP values, RC15 is optimal compared to no vaccination at lower hospitalisation rates, i.e. at a WTP of $200 per DALY averted, RC15 is optimal compared to no vaccination if the probability is at least 0·01.

Appendix Table 23 shows the minimum typhoid incidence rate (per 100,000 person-years) for which routine vaccination with a catch-up campaign up to age 15 years (RC15) becomes optimal, for the countries and WTP values for which the uncertainty around typhoid incidence influences the optimal strategy. For example, for Afghanistan, uncertainty around typhoid incidence influences the optimal strategy at a WTP value of $100 per DALY averted: RC15 is optimal compared to no vaccination if typhoid incidence is at least 330 symptomatic cases per 100,000 person-years. For Bangladesh, uncertainty around typhoid incidence does not influence the uncertainty around the optimal strategy, i.e. the EVPPI of incidence is low for Bangladesh; see https://ceatyphoid.uantwerpen.be/home/. Indeed, the optimal strategy is the same for each value of typhoid incidence considered for Bangladesh in our analysis (Appendix Table 6).

Appendix Table 24 shows the minimum typhoid case fatality rate among hospitalised patients (CFR_{hosp}) for which routine vaccination with a catch-up campaign up to age 15 years (RC15) becomes optimal compared to no vaccination, for the countries and WTP values for which the uncertainty around typhoid CFR_{hosp} influences the optimal strategy. For Burkina Faso, for example, uncertainty around the typhoid CFR_{hosp} influences the optimal strategy for a WTP value of $100 per DALY averted: RC15 is optimal compared to no vaccination if the CFR_{hosp} is at least 0·03. For Benin, the uncertainty around the optimal strategy is not due to uncertainty around the CFR_{hosp} (the EVPPI of the probability of death among inpatients is low for Benin; see https://ceatyphoid.uantwerpen.be/home/). Indeed, the optimal strategy is the same for each value of the typhoid CFR_{hosp} considered for Benin in our analysis (see Table 2 in main text).

Appendix Table 22: Minimum probability of hospitalisation for typhoid fever for which vaccination is optimal for a range of willingness-to-pay (WTP) values. Threshold values are only shown for countries and willingness-to-pay values for which the uncertainty around typhoid incidence influences the optimal intervention strategy (either no vaccination 'No' or routine vaccination + catch-up campaign up to age 15 years 'RC15'). For countries and WTP values for which no values are shown, the **optimal** strategy is the same for each value of the typhoid hospitalisation rate considered for that country in our analysis.

	$0	$100	$200	$300	$400	$500	$600	$700	$800	$900	$1000	$1100	$1200	$1300	$1400	$1500	$1600	$1700	$1800	$1900	$2000
Afghanistan	No	0.03	0.01	0.01	0.01	RC15	RC15	RC15	RC15	RC15	RC15	RC15	RC15	RC15	RC15	RC15	RC15	RC15	RC15	RC15	RC15
Bangladesh	0.16	0.02	0.01	0.01	RC15	RC15	RC15	RC15	RC15	RC15	RC15	RC15	RC15	RC15	RC15	RC15	RC15	RC15	RC15	RC15	RC15
Benin	0.1	0.01	0.01	RC15	RC15	RC15	RC15	RC15	RC15	RC15	RC15	RC15	RC15	RC15	RC15	RC15	RC15	RC15	RC15	RC15	RC15
Burkina Faso	0.16	0.04	0.01	0.01	0.01	0.01	RC15	RC15	RC15	RC15	RC15	RC15	RC15	RC15	RC15	RC15	RC15	RC15	RC15	RC15	RC15
Burundi	0.16	0.01	0.01	RC15	RC15	RC15	RC15	RC15	RC15	RC15	RC15	RC15	RC15	RC15	RC15	RC15	RC15	RC15	RC15	RC15	RC15
Cambodia	0.1	0.01	0.01	0.01	RC15	RC15	RC15	RC15	RC15	RC15	RC15	RC15	RC15	RC15	RC15	RC15	RC15	RC15	RC15	RC15	RC15
Cameroon	No	0.1	0.04	0.04	0.03	0.02	0.01	0.01	0.01	0.01	0.01	0.01	0.01	0.01	0.01	0.01	0.01	RC15	RC15	RC15	RC15
Central African Republic	0.16	0.03	0.01	0.01	0.01	RC15	RC15	RC15	RC15	RC15	RC15	RC15	RC15	RC15	RC15	RC15	RC15	RC15	RC15	RC15	RC15
Chad	0.16	0.02	0.01	0.01	0.01	RC15	RC15	RC15	RC15	RC15	RC15	RC15	RC15	RC15	RC15	RC15	RC15	RC15	RC15	RC15	RC15
Comoros	No	No	0.16	0.1	0.07	0.07	0.04	0.04	0.04	0.04	0.03	0.03	0.03	0.02	0.02	0.02	0.02	0.01	0.01	0.01	0.01
Dem Republic of Congo	0.16	0.02	0.01	0.01	RC15	RC15	RC15	RC15	RC15	RC15	RC15	RC15	RC15	RC15	RC15	RC15	RC15	RC15	RC15	RC15	RC15
Cote dIvoire	No	0.1	0.04	0.03	0.02	0.02	0.01	0.01	0.01	0.01	0.01	0.01	0.01	0.01	0.01	NA	NA	NA	NA	NA	NA
Djibouti	No	0.16	0.1	0.07	0.07	0.04	0.04	0.04	0.03	0.03	0.02	0.02	0.02	0.01	0.01	0.01	0.01	0.01	0.01	0.01	0.01
Eritrea	No	0.05	0.03	0.02	0.01	0.01	0.01	0.01	0.01	0.01	RC15	RC15	RC15	RC15	RC15	RC15	RC15	RC15	RC15	RC15	RC15
Ethiopia	No	0.02	0.01	0.01	RC15	RC15	RC15	RC15	RC15	RC15	RC15	RC15	RC15	RC15	RC15	RC15	RC15	RC15	RC15	RC15	RC15
Gambia	No	0.1	0.07	0.04	0.03	0.02	0.02	0.01	0.01	0.01	0.01	0.01	0.01	0.01	0.01	0.01	0.01	0.01	0.01	0.01	RC15
Ghana	No	0.16	0.07	0.04	0.04	0.03	0.03	0.02	0.02	0.01	0.01	0.01	0.01	0.01	0.01	0.01	0.01	0.01	0.01	0.01	0.01
Guinea	No	0.04	0.01	0.01	0.01	0.01	RC15	RC15	RC15	RC15	RC15	RC15	RC15	RC15	RC15	RC15	RC15	RC15	RC15	RC15	RC15
Guinea Bissau	No	0.1	0.07	0.04	0.03	0.03	0.02	0.02	0.01	0.01	0.01	0.01	0.01	0.01	0.01	0.01	0.01	0.01	0.01	0.01	0.01
Haiti	No	0.16	0.1	0.07	0.04	0.04	0.03	0.02	0.02	0.01	0.01	0.01	0.01	0.01	0.01	0.01	0.01	0.01	0.01	0.01	0.01
India	No	0.09	0.06	0.04	0.04	RC15	RC15	RC15	RC15	RC15	RC15	RC15	RC15	RC15	RC15	RC15	RC15	RC15	RC15	RC15	RC15
Kenya	No	0.1	0.06	0.04	0.03	0.02	0.02	0.01	0.01	0.01	0.01	0.01	RC15	RC15	RC15	RC15	RC15	RC15	RC15	RC15	RC15
DPR Korea	No	0.04	0.02	0.01	0.01	0.01	0.01	RC15	RC15	RC15	RC15	RC15	RC15	RC15	RC15	RC15	RC15	RC15	RC15	RC15	RC15
Kyrgyz Republic	No	No	No	No	No	No	No	No	No	No	No	No	No	0.16	0.16	0.16	0.16	0.16	0.16	0.16	0.16
Lao PDR	0.16	0.05	0.02	0.02	0.01	0.01	0.01	0.01	0.01	RC15	RC15	RC15	RC15	RC15	RC15	RC15	RC15	RC15	RC15	RC15	RC15
Lesotho	No	No	No	0.16	0.16	0.16	0.16	0.16	0.1	0.1	0.1	0.07	0.07	0.07	0.07	0.07	0.05	0.05	0.04	0.04	0.04
Liberia	0.16	0.02	0.01	0.01	RC15	RC15	RC15	RC15	RC15	RC15	RC15	RC15	RC15	RC15	RC15	RC15	RC15	RC15	RC15	RC15	RC15
Madagascar	0.16	0.02	0.01	RC15	RC15	RC15	RC15	RC15	RC15	RC15	RC15	RC15	RC15	RC15	RC15	RC15	RC15	RC15	RC15	RC15	RC15
Malawi	No	0.16	0.07	0.04	0.04	0.03	0.03	0.02	0.01	0.01	0.01	0.01	0.01	0.01	0.01	0.01	0.01	0.01	0.01	0.01	0.01

Mali	No	0.07	0.03	0.02	0.01	0.01	0.01	0.01	0.01	0.01	0.01	RC15	RC15	RC15	RC15	RC15	RC15	RC15	RC15	RC15	RC15
Mauritania	No	0.1	0.04	0.04	0.03	0.02	0.01	0.01	0.01	0.01	0.01	0.01	0.01	0.01	0.01	0.01	0.01	RC15	RC15	RC15	RC15
Mozambique	No	0.1	0.04	0.03	0.02	0.01	0.01	0.01	0.01	0.01	0.01	0.01	0.01	0.01	RC15	RC15	RC15	RC15	RC15	RC15	RC15
Myanmar	No	0.04	0.03	0.01	0.01	0.01	0.01	0.01	0.01	RC15	RC15	RC15	RC15	RC15	RC15	RC15	RC15	RC15	RC15	RC15	RC15
Nepal	No	0.04	0.02	0.01	0.01	0.01	0.01	0.01	RC15	RC15	RC15	RC15	RC15	RC15	RC15	RC15	RC15	RC15	RC15	RC15	RC15
Nicaragua	No	NA	0.16	0.1	0.1	0.07	0.07	0.07	0.04	0.04	0.04	0.04	0.04	0.03	0.03	0.03	0.03	0.03	0.02	0.02	0.02
Niger	No	0.07	0.03	0.02	0.01	0.01	0.01	0.01	0.01	0.01	RC15	RC15	RC15	RC15	RC15	RC15	RC15	RC15	RC15	RC15	RC15
Nigeria	0.16	0.02	0.01	0.01	0.01	RC15	RC15	RC15	RC15	RC15	RC15	RC15	RC15	RC15	RC15	RC15	RC15	RC15	RC15	RC15	RC15
Pakistan	No	No	No	0.03	0.03	0.02	0.02	0.02	0.01	0.01	0.01	0.01	0.01	0.01	0.01	0.01	0.01	0.01	0.01	RC15	RC15
Papua New Guinea	0.01	RC15	RC15	RC15	RC15	RC15	RC15	RC15	RC15	RC15	RC15	RC15	RC15	RC15	RC15	RC15	RC15	RC15	RC15	RC15	RC15
Rwanda	No	0.04	0.02	0.01	0.01	0.01	0.01	0.01	0.01	RC15	RC15	RC15	RC15	RC15	RC15	RC15	RC15	RC15	RC15	RC15	RC15
Sao Tome e Principe	No	No	0.16	0.16	0.1	0.1	0.07	0.07	0.04	0.04	0.04	0.04	0.04	0.04	0.04	0.03	0.03	0.03	0.02	0.02	0.02
Senegal	No	0.16	0.1	0.1	0.07	0.05	0.04	0.04	0.03	0.03	0.02	0.02	0.02	0.02	0.02	0.01	0.01	0.01	0.01	0.01	0.01
Sierra Leone	0.16	0.03	0.01	0.01	0.01	RC15	RC15	RC15	RC15	RC15	RC15	RC15	RC15	RC15	RC15	RC15	RC15	RC15	RC15	RC15	RC15
Solomon Islands	0.05	0.02	0.01	0.01	RC15	RC15	RC15	RC15	RC15	RC15	RC15	RC15	RC15	RC15	RC15	RC15	RC15	RC15	RC15	RC15	RC15
Somalia	No	0.04	0.02	0.01	0.01	0.01	0.01	0.01	RC15	RC15	RC15	RC15	RC15	RC15	RC15	RC15	RC15	RC15	RC15	RC15	RC15
Republic of Sudan	No	0.16	0.1	0.07	0.07	0.04	0.04	0.04	0.03	0.03	0.02	0.02	0.02	0.01	0.01	0.01	0.01	0.01	0.01	0.01	0.01
South Sudan	0.16	0.04	0.03	0.02	0.01	0.01	0.01	0.01	0.01	0.01	0.01	RC15	RC15	RC15	RC15	RC15	RC15	RC15	RC15	RC15	RC15
Tajikistan	No	No	No	No	No	No	No	No	No	No	No	No	No	0.16	0.16	0.16	0.16	0.16	0.16	0.16	0.16
Tanzania	No	0.07	0.03	0.02	0.01	0.01	0.01	0.01	0.01	RC15	RC15	RC15	RC15	RC15	RC15	RC15	RC15	RC15	RC15	RC15	RC15
Togo	No	0.1	0.04	0.04	0.03	0.02	0.01	0.01	0.01	0.01	0.01	0.01	0.01	0.01	0.01	0.01	RC15	RC15	RC15	RC15	RC15
Uganda	No	0.16	0.1	0.04	0.04	0.04	0.03	0.02	0.02	0.01	0.01	0.01	0.01	0.01	0.01	0.01	0.01	0.01	0.01	0.01	0.01
Yemen	0.03	0.01	RC15	RC15	RC15	RC15	RC15	RC15	RC15	RC15	RC15	RC15	RC15	RC15	RC15	RC15	RC15	RC15	RC15	RC15	RC15
Zambia	No	0.16	0.07	0.04	0.04	0.03	0.02	0.02	0.01	0.01	0.01	0.01	0.01	0.01	0.01	0.01	0.01	0.01	0.01	0.01	0.01
Zimbabwe	No	0.1	0.07	0.04	0.03	0.02	0.02	0.02	0.01	0.01	0.01	0.01	0.01	0.01	0.01	0.01	0.01	0.01	0.01	RC15	RC15

Appendix Table 23: Minimum typhoid incidence rate for which vaccination is optimal for a range of willingness-to-pay (WTP) values. Threshold values are only shown for countries and willingness-to-pay values for which the uncertainty around typhoid incidence (cases per 100,000 person-years) influences the **optimal** intervention strategy (either no vaccination 'No' or routine vaccination + catch-up campaign up to age 15 years 'RC15'). For countries and WTP values for which no values are shown, the **optimal** strategy is the same for each value of typhoid incidence considered for that country in our analysis.

	$0	$100	$200	$300	$400	$500	$600	$700	$800	$900	$1000	$1100	$1200	$1300	$1400	$1500	$1600	$1700	$1800	$1900	$2000
Afghanistan	No	330	RC15	RC15	RC15	RC15	RC15	RC15	RC15	RC15	RC15	RC15	RC15	RC15	RC15	RC15	RC15	RC15	RC15	RC15	RC15
Bangladesh	No	RC15	RC15	RC15	RC15	RC15	RC15	RC15	RC15	RC15	RC15	RC15	RC15	RC15	RC15	RC15	RC15	RC15	RC15	RC15	RC15
Benin	1305	369	RC15	RC15	RC15	RC15	RC15	RC15	RC15	RC15	RC15	RC15	RC15	RC15	RC15	RC15	RC15	RC15	RC15	RC15	RC15
Burkina Faso	No	RC15	RC15	RC15	RC15	RC15	RC15	RC15	RC15	RC15	RC15	RC15	RC15	RC15	RC15	RC15	RC15	RC15	RC15	RC15	RC15
Burundi	No	322	RC15	RC15	RC15	RC15	RC15	RC15	RC15	RC15	RC15	RC15	RC15	RC15	RC15	RC15	RC15	RC15	RC15	RC15	RC15
Cambodia	No	RC15	RC15	RC15	RC15	RC15	RC15	RC15	RC15	RC15	RC15	RC15	RC15	RC15	RC15	RC15	RC15	RC15	RC15	RC15	RC15
Cameroon	No	275	204	RC15	RC15	RC15	RC15	RC15	RC15	RC15	RC15	RC15	RC15	RC15	RC15	RC15	RC15	RC15	RC15	RC15	RC15
Central African Republic	No	272	RC15	RC15	RC15	RC15	RC15	RC15	RC15	RC15	RC15	RC15	RC15	RC15	RC15	RC15	RC15	RC15	RC15	RC15	RC15
Chad	No	RC15	RC15	RC15	RC15	RC15	RC15	RC15	RC15	RC15	RC15	RC15	RC15	RC15	RC15	RC15	RC15	RC15	RC15	RC15	RC15
Comoros	No	No	No	82	82	69	63	63	RC15	RC15	RC15	RC15	RC15	RC15	RC15	RC15	RC15	RC15	RC15	RC15	RC15
Dem Republic of Congo	No	275	RC15	RC15	RC15	RC15	RC15	RC15	RC15	RC15	RC15	RC15	RC15	RC15	RC15	RC15	RC15	RC15	RC15	RC15	RC15
Cote dIvoire	No	492	244	RC15	RC15	RC15	RC15	RC15	RC15	RC15	RC15	RC15	RC15	RC15	RC15	RC15	RC15	RC15	RC15	RC15	RC15
Djibouti	No	No	No	91	70	RC15	RC15	RC15	RC15	RC15	RC15	RC15	RC15	RC15	RC15	RC15	RC15	RC15	RC15	RC15	RC15
Eritrea	No	266	180	180	RC15	RC15	RC15	RC15	RC15	RC15	RC15	RC15	RC15	RC15	RC15	RC15	RC15	RC15	RC15	RC15	RC15
Ethiopia	No	RC15	RC15	RC15	RC15	RC15	RC15	RC15	RC15	RC15	RC15	RC15	RC15	RC15	RC15	RC15	RC15	RC15	RC15	RC15	RC15
Gambia	No	No	165	RC15	RC15	RC15	RC15	RC15	RC15	RC15	RC15	RC15	RC15	RC15	RC15	RC15	RC15	RC15	RC15	RC15	RC15
Ghana	No	No	216	211	RC15	RC15	RC15	RC15	RC15	RC15	RC15	RC15	RC15	RC15	RC15	RC15	RC15	RC15	RC15	RC15	RC15
Guinea	No	328	RC15	RC15	RC15	RC15	RC15	RC15	RC15	RC15	RC15	RC15	RC15	RC15	RC15	RC15	RC15	RC15	RC15	RC15	RC15
Guinea Bissau	No	279	173	85	85	85	85	59	59	59	RC15	RC15	RC15	RC15	RC15	RC15	RC15	RC15	RC15	RC15	RC15
Haiti	No	198	157	111	92	71	50	50	50	29	29	29	29	29	29	29	29	29	29	RC15	RC15
India	No	498	359	228	178	134	134	134	RC15	RC15	RC15	RC15	RC15	RC15	RC15	RC15	RC15	RC15	RC15	RC15	RC15
Kenya	No	225	179	RC15	RC15	RC15	RC15	RC15	RC15	RC15	RC15	RC15	RC15	RC15	RC15	RC15	RC15	RC15	RC15	RC15	RC15
DPR Korea	No	112	112	60	60	60	60	RC15	RC15	RC15	RC15	RC15	RC15	RC15	RC15	RC15	RC15	RC15	RC15	RC15	RC15
Kyrgyz Republic	No	No	No	No	No	No	No	No	No	No	No	No	No	No	No	No	No	No	No	No	No
Lao PDR	No	560	RC15	RC15	RC15	RC15	RC15	RC15	RC15	RC15	RC15	RC15	RC15	RC15	RC15	RC15	RC15	RC15	RC15	RC15	RC15
Lesotho	No	No	No	No	No	No	41	41	41	41	31	31	31	26	26	26	26	26	22	22	22
Liberia	No	322	RC15	RC15	RC15	RC15	RC15	RC15	RC15	RC15	RC15	RC15	RC15	RC15	RC15	RC15	RC15	RC15	RC15	RC15	RC15
Madagascar	No	258	RC15	RC15	RC15	RC15	RC15	RC15	RC15	RC15	RC15	RC15	RC15	RC15	RC15	RC15	RC15	RC15	RC15	RC15	RC15
Malawi	No	No	172	109	RC15	RC15	RC15	RC15	RC15	RC15	RC15	RC15	RC15	RC15	RC15	RC15	RC15	RC15	RC15	RC15	RC15

Mali	No	271	RC15	RC15	RC15	RC15	RC15	RC15	RC15	RC15	RC15	RC15	RC15	RC15	RC15	RC15	RC15	RC15	RC15	RC15	RC15
Mauritania	No	No	191	RC15	RC15	RC15	RC15	RC15	RC15	RC15	RC15	RC15	RC15	RC15	RC15	RC15	RC15	RC15	RC15	RC15	RC15
Mozambique	No	241	RC15	RC15	RC15	RC15	RC15	RC15	RC15	RC15	RC15	RC15	RC15	RC15	RC15	RC15	RC15	RC15	RC15	RC15	RC15
Myanmar	No	193	RC15	RC15	RC15	RC15	RC15	RC15	RC15	RC15	RC15	RC15	RC15	RC15	RC15	RC15	RC15	RC15	RC15	RC15	RC15
Nepal	No	229	RC15	RC15	RC15	RC15	RC15	RC15	RC15	RC15	RC15	RC15	RC15	RC15	RC15	RC15	RC15	RC15	RC15	RC15	RC15
Nicaragua	No	No	No	124	124	95	77	77	62	48	48	33	33	33	33	33	33	33	33	18	18
Niger	No	296	RC15	RC15	RC15	RC15	RC15	RC15	RC15	RC15	RC15	RC15	RC15	RC15	RC15	RC15	RC15	RC15	RC15	RC15	RC15
Nigeria	No	RC15	RC15	RC15	RC15	RC15	RC15	RC15	RC15	RC15	RC15	RC15	RC15	RC15	RC15	RC15	RC15	RC15	RC15	RC15	RC15
Pakistan	No	No	No	No	No	298	260	205	165	165	165	RC15	RC15	RC15	RC15	RC15	RC15	RC15	RC15	RC15	RC15
Papua New Guinea	RC15	RC15	RC15	RC15	RC15	RC15	RC15	RC15	RC15	RC15	RC15	RC15	RC15	RC15	RC15	RC15	RC15	RC15	RC15	RC15	RC15
Rwanda	No	221	RC15	RC15	RC15	RC15	RC15	RC15	RC15	RC16	RC17	RC18	RC19	RC20	RC21	RC22	RC23	RC24	RC25	RC26	RC27
Sao Tome e Principe	No	No	No	No	122	112	75	75	75	55	45	45	45	45	45	45	45	45	45	45	RC15
Senegal	No	No	No	153	148	133	RC15	RC15	RC15	RC15	RC15	RC15	RC15	RC15	RC15	RC15	RC15	RC15	RC15	RC15	RC15
Sierra Leone	No	RC15	RC15	RC15	RC15	RC15	RC15	RC15	RC15	RC15	RC15	RC15	RC15	RC15	RC15	RC15	RC15	RC15	RC15	RC15	RC15
Solomon Islands	842	300	RC15	RC15	RC15	RC15	RC15	RC15	RC15	RC15	RC15	RC15	RC15	RC15	RC15	RC15	RC15	RC15	RC15	RC15	RC15
Somalia	No	306	RC15	RC15	RC15	RC15	RC15	RC15	RC15	RC15	RC15	RC15	RC15	RC15	RC15	RC15	RC15	RC15	RC15	RC15	RC15
Republic of Sudan	No	No	223	165	140	140	96	96	96	RC15	RC15	RC15	RC15	RC15	RC15	RC15	RC15	RC15	RC15	RC15	RC15
South Sudan	No	172	155	RC15	RC15	RC15	RC15	RC15	RC15	RC15	RC15	RC15	RC15	RC15	RC15	RC15	RC15	RC15	RC15	RC15	RC15
Tajikistan	No	No	No	No	No	No	No	No	No	No	No	No	No	No	No	No	No	No	No	No	No
Tanzania	No	321	123	RC15	RC15	RC15	RC15	RC15	RC15	RC15	RC15	RC15	RC15	RC15	RC15	RC15	RC15	RC15	RC15	RC15	RC15
Togo	No	No	189	RC15	RC15	RC15	RC15	RC15	RC15	RC15	RC15	RC15	RC15	RC15	RC15	RC15	RC15	RC15	RC15	RC15	RC15
Uganda	No	No	140	123	RC15	RC15	RC15	RC15	RC15	RC15	RC15	RC15	RC15	RC15	RC15	RC15	RC15	RC15	RC15	RC15	RC15
Yemen	1329	RC15	RC15	RC15	RC15	RC15	RC15	RC15	RC15	RC15	RC15	RC15	RC15	RC15	RC15	RC15	RC15	RC15	RC15	RC15	RC15
Zambia	No	No	318	193	RC15	RC15	RC15	RC15	RC15	RC15	RC15	RC15	RC15	RC15	RC15	RC15	RC15	RC15	RC15	RC15	RC15
Zimbabwe	No	276	124	90	90	57	32	32	32	32	32	32	32	32	32	32	32	RC15	RC15	RC15	RC15

Appendix Table 24: Minimum case fatality rate among hospitalised patients (CFR_{hosp}) for which vaccination is optimal for a range of willingness-to-pay (WTP) values. Threshold values are only shown for countries and willingness-to-pay values for which the uncertainty around CFR_{hosp} influences the **optimal** intervention strategy (either no vaccination 'No' or routine vaccination + catch-up campaign up to age 15 years 'RC15'). For countries and WTP values for which no values are shown, the **optimal** strategy is the same for each value of typhoid CFR_{hosp} considered for that country in our analysis.

	$0	$100	$200	$300	$400	$500	$600	$700	$800	$900	$1000	$1100	$1200	$1300	$1400	$1500	$1600	$1700	$1800	$1900	$2000
Afghanistan	No	0.03	RC15	RC15	RC15	RC15	RC15	RC15	RC15	RC15	RC15	RC15	RC15	RC15	RC15	RC15	RC15	RC15	RC15	RC15	RC15
Bangladesh	No	0.01	RC15	RC15	RC15	RC15	RC15	RC15	RC15	RC15	RC15	RC15	RC15	RC15	RC15	RC15	RC15	RC15	RC15	RC15	RC15
Benin	No	RC15	RC15	RC15	RC15	RC15	RC15	RC15	RC15	RC15	RC15	RC15	RC15	RC15	RC15	RC15	RC15	RC15	RC15	RC15	RC15
Burkina Faso	No	0.03	RC15	RC15	RC15	RC15	RC15	RC15	RC15	RC15	RC15	RC15	RC15	RC15	RC15	RC15	RC15	RC15	RC15	RC15	RC15
Burundi	No	RC15	RC15	RC15	RC15	RC15	RC15	RC15	RC15	RC15	RC15	RC15	RC15	RC15	RC15	RC15	RC15	RC15	RC15	RC15	RC15
Cambodia	No	RC15	RC15	RC15	RC15	RC15	RC15	RC15	RC15	RC15	RC15	RC15	RC15	RC15	RC15	RC15	RC15	RC15	RC15	RC15	RC15
Cameroon	No	0.07	0.05	0.03	0.02	0.02	RC15	RC15	RC15	RC15	RC15	RC15	RC15	RC15	RC15	RC15	RC15	RC15	RC15	RC15	RC15
Central African Republic	No	0.03	RC15	RC15	RC15	RC15	RC15	RC15	RC15	RC15	RC15	RC15	RC15	RC15	RC15	RC15	RC15	RC15	RC15	RC15	RC15
Chad	No	RC15	RC15	RC15	RC15	RC15	RC15	RC15	RC15	RC15	RC15	RC15	RC15	RC15	RC15	RC15	RC15	RC15	RC15	RC15	RC15
Comoros	No	No	0.13	0.09	0.07	0.05	0.05	0.05	0.03	0.03	0.03	0.03	0.02	0.02	0.02	0.02	0.02	0.02	0.02	0.02	RC15
Dem Republic of Congo	No	0.02	RC15	RC15	RC15	RC15	RC15	RC15	RC15	RC15	RC15	RC15	RC15	RC15	RC15	RC15	RC15	RC15	RC15	RC15	RC15
Cote dIvoire	No	0.07	0.03	0.02	RC15	RC15	RC15	RC15	RC15	RC15	RC15	RC15	RC15	RC15	RC15	RC15	RC15	RC15	RC15	RC15	RC15
Djibouti	No	No	0.09	0.07	0.05	0.05	0.03	0.03	0.03	0.03	0.02	0.02	0.02	0.02	0.02	RC15	RC15	RC15	RC15	RC15	RC15
Eritrea	No	0.05	0.03	0.02	RC15	RC15	RC15	RC15	RC15	RC15	RC15	RC15	RC15	RC15	RC15	RC15	RC15	RC15	RC15	RC15	RC15
Ethiopia	No	0.04	RC15	RC15	RC15	RC15	RC15	RC15	RC15	RC15	RC15	RC15	RC15	RC15	RC15	RC15	RC15	RC15	RC15	RC15	RC15
Gambia	No	0.13	0.05	0.03	0.03	0.02	0.02	0.02	RC15	RC15	RC15	RC15	RC15	RC15	RC15	RC15	RC15	RC15	RC15	RC15	RC15
Ghana	No	0.13	0.07	0.05	0.03	0.03	0.02	0.02	0.02	0.02	RC15	RC15	RC15	RC15	RC15	RC15	RC15	RC15	RC15	RC15	RC15
Guinea	No	0.03	RC15	RC15	RC15	RC15	RC15	RC15	RC15	RC15	RC15	RC15	RC15	RC15	RC15	RC15	RC15	RC15	RC15	RC15	RC15
Guinea Bissau	No	0.13	0.05	0.05	0.03	0.02	0.02	0.02	0.02	0.02	RC15	RC15	RC15	RC15	RC15	RC15	RC15	RC15	RC15	RC15	RC15
Haiti	No	0.13	0.07	0.05	0.05	0.03	0.03	0.02	0.02	0.02	RC15	RC15	RC15	RC15	RC15	RC15	RC15	RC15	RC15	RC15	RC15
India	No	No	0.07	0.04	RC15	RC15	RC15	RC15	RC15	RC15	RC15	RC15	RC15	RC15	RC15	RC15	RC15	RC15	RC15	RC15	RC15
Kenya	No	0.07	0.04	0.03	0.02	0.02	RC15	RC15	RC15	RC15	RC15	RC15	RC15	RC15	RC15	RC15	RC15	RC15	RC15	RC15	RC15
DPR Korea	No	0.03	0.02	RC15	RC15	RC15	RC15	RC15	RC15	RC15	RC15	RC15	RC15	RC15	RC15	RC15	RC15	RC15	RC15	RC15	RC15
Kyrgyz Republic	No	No	No	No	No	No	No	No	No	No	No	No	No	No	No	No	No	No	0.13	0.13	0.13
Lao PDR	No	0.01	0.01	RC15	RC15	RC15	RC15	RC15	RC15	RC15	RC15	RC15	RC15	RC15	RC15	RC15	RC15	RC15	RC15	RC15	RC15
Lesotho	No	No	No	No	No	0.13	0.13	0.13	0.09	0.07	0.07	0.07	0.07	0.06	0.05	0.05	0.05	0.05	0.05	0.05	0.05
Liberia	No	RC15	RC15	RC15	RC15	RC15	RC15	RC15	RC15	RC15	RC15	RC15	RC15	RC15	RC15	RC15	RC15	RC15	RC15	RC15	RC15
Madagascar	No	RC15	RC15	RC15	RC15	RC15	RC15	RC15	RC15	RC15	RC15	RC15	RC15	RC15	RC15	RC15	RC15	RC15	RC15	RC15	RC15
Malawi	No	0.13	0.07	0.05	0.03	0.03	0.02	0.02	0.02	RC15	RC15	RC15	RC15	RC15	RC15	RC15	RC15	RC15	RC15	RC15	RC15
Mali	No	0.05	0.03	0.02	RC15	RC15	RC15	RC15	RC15	RC15	RC15	RC15	RC15	RC15	RC15	RC15	RC15	RC15	RC15	RC15	RC15

Mauritania	No	0.07	0.05	0.03	0.02	0.02	RC15	RC15	RC15	RC15	RC15	RC15	RC15	RC15	RC15	RC15	RC15	RC15	RC15	RC15	RC15
Mozambique	No	0.07	0.05	0.03	0.02	0.02	RC15	RC15	RC15	RC15	RC15	RC15	RC15	RC15	RC15	RC15	RC15	RC15	RC15	RC15	RC15
Myanmar	No	0.05	0.02	RC15	RC15	RC15	RC15	RC15	RC15	RC15	RC15	RC15	RC15	RC15	RC15	RC15	RC15	RC15	RC15	RC15	RC15
Nepal	No	0.03	0.02	RC15	RC15	RC15	RC15	RC15	RC15	RC15	RC15	RC15	RC15	RC15	RC15	RC15	RC15	RC15	RC15	RC15	RC15
Nicaragua	No	No	No	0.13	0.07	0.07	0.06	0.05	0.05	0.03	0.03	0.03	0.03	0.03	0.03	0.02	0.02	0.02	0.02	0.02	0.02
Niger	No	0.05	0.03	0.02	RC15	RC15	RC15	RC15	RC15	RC15	RC15	RC15	RC15	RC15	RC15	RC15	RC15	RC15	RC15	RC15	RC15
Nigeria	No	RC15	RC15	RC15	RC15	RC15	RC15	RC15	RC15	RC15	RC15	RC15	RC15	RC15	RC15	RC15	RC15	RC15	RC15	RC15	RC15
Pakistan	No	No	No	0.13	0.09	0.07	0.07	0.06	0.06	0.05	0.04	0.04	0.03	0.03	0.03	0.02	0.02	0.02	0.02	0.02	0.02
Papua New Guinea	RC15	RC15	RC15	RC15	RC15	RC15	RC15	RC15	RC15	RC15	RC15	RC15	RC15	RC15	RC15	RC15	RC15	RC15	RC15	RC15	RC15
Rwanda	No	0.04	0.02	RC15	RC15	RC15	RC15	RC15	RC15	RC15	RC15	RC15	RC15	RC15	RC15	RC15	RC15	RC15	RC15	RC15	RC15
Sao Tome e Principe	No	No	No	0.13	0.09	0.07	0.07	0.05	0.05	0.05	0.05	0.03	0.03	0.03	0.03	0.03	0.02	0.02	0.02	0.02	0.02
Senegal	No	No	0.06	0.04	0.03	0.02	0.02	0.02	0.02	0.02	0.02	RC15	RC15	RC15	RC15	RC15	RC15	RC15	RC15	RC15	RC15
Sierra Leone	No	0.02	RC15	RC15	RC15	RC15	RC15	RC15	RC15	RC15	RC15	RC15	RC15	RC15	RC15	RC15	RC15	RC15	RC15	RC15	RC15
Solomon Islands	RC15	RC15	RC15	RC15	RC15	RC15	RC15	RC15	RC15	RC15	RC15	RC15	RC15	RC15	RC15	RC15	RC15	RC15	RC15	RC15	RC15
Somalia	No	0.05	0.02	RC15	RC15	RC15	RC15	RC15	RC15	RC15	RC15	RC15	RC15	RC15	RC15	RC15	RC15	RC15	RC15	RC15	RC15
Republic of Sudan	No	No	0.13	0.07	0.05	0.05	0.03	0.03	0.03	0.02	0.02	0.02	0.02	0.02	RC15	RC15	RC15	RC15	RC15	RC15	RC15
South Sudan	No	0.05	0.03	RC15	RC15	RC15	RC15	RC15	RC15	RC15	RC15	RC15	RC15	RC15	RC15	RC15	RC15	RC15	RC15	RC15	RC15
Tajikistan	No	No	No	No	No	No	No	No	No	No	No	No	No	No	No	No	No	No	No	No	No
Tanzania	No	0.05	0.03	0.02	RC15	RC15	RC15	RC15	RC15	RC15	RC15	RC15	RC15	RC15	RC15	RC15	RC15	RC15	RC15	RC15	RC15
Togo	No	0.09	0.05	0.03	0.02	0.02	RC15	RC15	RC15	RC15	RC15	RC15	RC15	RC15	RC15	RC15	RC15	RC15	RC15	RC15	RC15
Uganda	No	0.13	0.07	0.05	0.04	0.03	0.03	0.02	0.02	0.02	0.02	RC15	RC15	RC15	RC15	RC15	RC15	RC15	RC15	RC15	RC15
Yemen	RC15	RC15	RC15	RC15	RC15	RC15	RC15	RC15	RC15	RC15	RC15	RC15	RC15	RC15	RC15	RC15	RC15	RC15	RC15	RC15	RC15
Zambia	No	0.13	0.06	0.05	0.03	0.02	0.02	0.02	0.02	RC15	RC15	RC15	RC15	RC15	RC15	RC15	RC15	RC15	RC15	RC15	RC15
Zimbabwe	No	0.12	0.06	0.05	RC15	RC15	RC15	RC15	RC15	RC15	RC15	RC15	RC15	RC15	RC15	RC15	RC15	RC15	RC15	RC15	RC15

3.4 Scenario analyses

3.4.1 Vaccine cost: including Gavi's share

Appendix Table 25 and Appendix Table 26 show the optimal intervention strategy for each country, and how certain we are about it, if we include the total vaccine procurement and delivery costs (country contribution + Gavi's support), i.e. a vaccine price of US$1·50 per dose for all countries for both routine and campaign doses, and the full country-specific delivery cost per dose for routine and campaign doses (shown in Appendix Table 16).

RC15 or RC5 are optimal in 19 countries when the WTP is ≥US$200 per DALY averted, in 37 countries at a WTP ≥US$500 per DALY averted, and in 47 countries at a WTP ≥US$1000 per DALY averted (Appendix Table 25). For three countries (Kyrgyz Republic, Lesotho, and Tajikistan), none of the vaccination programs is optimal at WTP values <US$2000 per DALY averted. In terms of GDP per capita, RC15 or RC5 are optimal in 19 countries at WTP values ≥25% of the GDP per capita, in 35 countries at a WTP ≥50% of the GDP per capita, and in 42 countries at a WTP ≥100% of the GDP per capita (Appendix Table 26).

Appendix Table 25 and Appendix Table 26 indicate that including an initial catch-up campaign is always optimal when compared to routine immunisation alone when introducing TCVs. RC15 is consistently optimal when compared to RC5 and R for 34 countries. For 17 countries, RC5 is optimal when compared to RC15 and R for smaller WTP thresholds; for larger WTP values, RC15 becomes the optimal strategy. For these 17 countries, if RC5 is optimal, the probability that it results in highest net benefit never exceeds 25%.

Appendix Table 25: Cost-effectiveness results when including Gavi's share for each country for a range of willingness-to-pay values (US$0-US$2000 per DALY averted). The **optimal** strategy (i.e. strategy that yields the highest *average* net monetary benefit) is indicated by the colour-shading: no vaccination (white), routine childhood immunisation (green), routine immunisation with a catch-up campaign up to age 5 years (purple) and routine immunisation with a catch-up campaign up to age 15 years (orange). How certain we are about the **optimal** strategy is indicated by the percentage of parameter samples in which the **optimal** strategy yielded the highest net benefit.

	$0	$100	$200	$300	$400	$500	$600	$700	$800	$900	$1000	$1100	$1200	$1300	$1400	$1500	$1600	$1700	$1800	$1900	$2000
Afghanistan	99%	79%	29%	38%	44%	50%	55%	59%	62%	65%	68%	70%	72%	73%	75%	76%	77%	78%	80%	81%	82%
Bangladesh	95%	22%	32%	39%	44%	49%	53%	56%	59%	62%	64%	66%	68%	70%	71%	72%	73%	75%	76%	77%	78%
Benin	95%	25%	40%	50%	57%	63%	67%	70%	73%	75%	77%	78%	80%	81%	82%	83%	84%	85%	86%	87%	88%
Burkina Faso	100%	85%	6%	32%	40%	45%	50%	55%	59%	62%	65%	68%	71%	72%	74%	76%	77%	79%	80%	81%	83%
Burundi	98%	25%	40%	49%	55%	62%	66%	70%	72%	75%	76%	78%	80%	81%	82%	83%	84%	85%	86%	87%	88%
Cambodia	98%	9%	32%	42%	49%	55%	60%	64%	67%	70%	74%	77%	79%	80%	82%	83%	84%	85%	86%	87%	88%
Cameroon	100%	94%	87%	80%	74%	8%	27%	31%	34%	36%	39%	42%	44%	47%	48%	50%	52%	54%	55%	57%	58%
Central African Republic	99%	84%	24%	32%	40%	46%	50%	53%	57%	60%	63%	66%	68%	70%	72%	74%	75%	76%	77%	79%	80%
Chad	98%	80%	28%	38%	46%	51%	56%	60%	64%	67%	69%	72%	74%	76%	78%	79%	80%	81%	82%	83%	84%
Comoros	100%	100%	98%	96%	94%	92%	90%	88%	86%	84%	82%	80%	78%	76%	74%	24%	26%	27%	28%	30%	31%
Dem Republic of Congo	99%	76%	36%	46%	53%	58%	63%	67%	69%	72%	74%	76%	78%	80%	81%	83%	84%	84%	85%	86%	87%
Cote dIvoire	99%	90%	79%	7%	27%	32%	36%	40%	44%	47%	50%	53%	55%	57%	60%	61%	63%	65%	66%	68%	70%
Djibouti	100%	98%	95%	92%	89%	86%	83%	80%	76%	73%	7%	24%	26%	28%	29%	31%	33%	34%	35%	37%	38%
Eritrea	100%	91%	80%	25%	31%	36%	40%	44%	48%	51%	54%	57%	59%	61%	64%	67%	69%	71%	72%	73%	74%
Ethiopia	100%	5%	39%	50%	57%	63%	67%	71%	73%	75%	78%	80%	81%	83%	84%	85%	87%	88%	88%	89%	90%
Gambia	100%	96%	91%	87%	82%	77%	3%	27%	31%	33%	36%	38%	40%	42%	44%	46%	47%	50%	52%	54%	55%
Ghana	100%	96%	91%	85%	80%	76%	27%	31%	35%	38%	40%	43%	45%	47%	49%	51%	53%	54%	57%	58%	60%
Guinea	100%	87%	4%	30%	37%	44%	48%	53%	56%	60%	63%	65%	68%	70%	72%	74%	75%	77%	78%	79%	80%
Guinea Bissau	100%	97%	92%	88%	84%	80%	76%	24%	27%	29%	32%	34%	36%	38%	40%	42%	44%	46%	48%	49%	50%
Haiti	100%	98%	96%	93%	89%	86%	83%	80%	78%	76%	26%	28%	30%	32%	34%	35%	37%	38%	39%	40%	41%
India	100%	85%	63%	45%	56%	65%	71%	76%	80%	83%	85%	87%	88%	90%	92%	93%	93%	94%	95%	95%	96%
Kenya	100%	92%	82%	24%	32%	38%	43%	47%	51%	54%	57%	60%	62%	64%	66%	68%	70%	71%	73%	74%	76%
DPR Korea	100%	89%	77%	26%	34%	38%	42%	46%	49%	51%	55%	58%	60%	61%	63%	64%	66%	67%	68%	69%	70%
Kyrgyz Republic	100%	100%	100%	100%	100%	100%	100%	100%	99%	99%	99%	99%	98%	98%	98%	98%	97%	97%	97%	97%	96%
Lao PDR	96%	82%	9%	24%	29%	33%	38%	42%	45%	47%	50%	53%	55%	56%	58%	61%	62%	64%	66%	67%	68%
Lesotho	100%	100%	100%	99%	98%	98%	97%	96%	95%	94%	93%	92%	92%	91%	90%	89%	88%	88%	87%	86%	85%
Liberia	99%	77%	35%	46%	52%	58%	63%	68%	71%	73%	75%	77%	79%	81%	82%	83%	84%	85%	86%	87%	88%
Madagascar	99%	76%	36%	46%	54%	59%	63%	66%	70%	72%	74%	77%	78%	79%	81%	82%	83%	84%	85%	86%	87%
Malawi	100%	97%	94%	89%	85%	81%	77%	4%	26%	29%	31%	34%	36%	38%	39%	41%	43%	45%	46%	48%	50%
Mali	100%	91%	83%	75%	27%	33%	37%	41%	45%	49%	52%	55%	56%	59%	61%	63%	65%	65%	67%	68%	70%
Mauritania	100%	94%	88%	82%	76%	25%	30%	33%	37%	40%	42%	45%	47%	50%	52%	54%	56%	58%	60%	61%	62%

Mozambique	100%	95%	88%	81%	75%	26%	31%	35%	38%	40%	43%	46%	48%	51%	53%	55%	57%	59%	61%	63%	63%
Myanmar	99%	87%	75%	29%	35%	41%	46%	51%	54%	58%	60%	62%	65%	67%	69%	70%	73%	74%	76%	77%	78%
Nepal	100%	88%	77%	24%	30%	35%	40%	44%	49%	52%	55%	58%	60%	62%	64%	65%	67%	69%	70%	72%	73%
Nicaragua	100%	99%	96%	94%	92%	89%	87%	85%	82%	80%	79%	77%	24%	25%	27%	28%	30%	31%	33%	34%	35%
Niger	100%	92%	81%	4%	28%	34%	38%	42%	45%	49%	53%	56%	58%	60%	62%	64%	66%	67%	69%	70%	71%
Nigeria	98%	36%	56%	67%	74%	79%	83%	86%	88%	90%	92%	93%	94%	95%	96%	96%	96%	97%	97%	97%	98%
Pakistan	100%	100%	98%	96%	94%	91%	88%	85%	82%	78%	75%	73%	70%	7%	8%	29%	32%	34%	35%	37%	38%
Papua New Guinea	23%	55%	70%	77%	81%	84%	86%	88%	90%	91%	92%	93%	93%	94%	94%	95%	95%	96%	96%	96%	96%
Rwanda	100%	92%	83%	76%	7%	29%	33%	38%	41%	44%	47%	49%	52%	54%	56%	58%	60%	61%	63%	64%	66%
Sao Tome e Principe	100%	99%	97%	95%	92%	90%	87%	86%	84%	82%	80%	78%	76%	23%	25%	27%	28%	29%	31%	32%	33%
Senegal	100%	99%	96%	92%	89%	86%	83%	80%	77%	74%	27%	30%	32%	35%	36%	38%	40%	42%	44%	45%	47%
Sierra Leone	99%	82%	28%	37%	44%	51%	56%	61%	64%	67%	70%	72%	75%	76%	78%	79%	81%	82%	83%	84%	85%
Solomon Islands	84%	15%	36%	44%	50%	56%	60%	63%	66%	69%	71%	73%	75%	76%	78%	79%	80%	81%	82%	83%	84%
Somalia	100%	89%	77%	31%	38%	44%	49%	54%	57%	60%	62%	65%	67%	69%	71%	73%	74%	76%	77%	79%	80%
Republic of Sudan	100%	97%	93%	89%	85%	81%	77%	74%	7%	25%	28%	29%	30%	33%	34%	36%	37%	39%	41%	42%	43%
South Sudan	100%	92%	85%	78%	26%	31%	36%	39%	42%	45%	48%	51%	53%	56%	58%	59%	60%	62%	64%	66%	67%
Tajikistan	100%	100%	100%	100%	100%	100%	99%	99%	99%	99%	98%	98%	98%	98%	98%	98%	97%	97%	97%	96%	96%
Tanzania	100%	91%	80%	24%	30%	36%	41%	45%	49%	52%	56%	58%	61%	63%	65%	66%	68%	70%	71%	73%	73%
Togo	100%	96%	89%	83%	77%	4%	28%	32%	35%	37%	40%	43%	45%	48%	50%	52%	54%	56%	57%	59%	61%
Uganda	100%	98%	94%	91%	88%	84%	80%	77%	6%	7%	25%	28%	30%	31%	33%	36%	37%	39%	40%	42%	43%
Yemen	72%	45%	59%	68%	74%	77%	80%	82%	84%	85%	87%	88%	89%	89%	90%	91%	92%	92%	93%	93%	93%
Zambia	100%	95%	88%	82%	76%	29%	32%	36%	40%	43%	45%	48%	51%	52%	55%	57%	58%	60%	61%	63%	64%
Zimbabwe	100%	96%	91%	85%	80%	76%	25%	28%	32%	35%	38%	40%	42%	44%	46%	48%	50%	51%	53%	55%	56%

Appendix Table 26: Cost-effectiveness results when including Gavi's share for each country for a range of willingness-to-pay values (0 up to 4 times a country's GDP per capita). The **optimal** strategy (i.e. strategy that yields the highest *average* net monetary benefit) is indicated by the colour-shading: no vaccination (white), routine childhood immunisation (green), routine immunisation with a catch-up campaign up to age 5 years (purple) and routine immunisation with a catch-up campaign up to age 15 years (orange). How certain we are about the **optimal** strategy is indicated by the percentage of parameter samples in which the **optimal** strategy yielded the highest net benefit.

	0	0.25*GDP	0.5*GDP	0.75*GDP	1*GDP	1.5*GDP	2*GDP	3*GDP	4*GDP
Afghanistan	99%	4%	36%	46%	54%	63%	70%	78%	83%
Bangladesh	95%	41%	56%	65%	71%	79%	83%	90%	93%
Benin	95%	39%	56%	66%	72%	79%	84%	90%	93%
Burkina Faso	100%	76%	34%	45%	53%	64%	72%	82%	87%
Burundi	98%	74%	34%	44%	51%	61%	68%	76%	81%
Cambodia	98%	43%	60%	71%	79%	87%	91%	94%	96%
Cameroon	100%	79%	28%	37%	45%	56%	64%	75%	82%
Central African Republic	99%	86%	4%	30%	37%	48%	54%	66%	73%
Chad	98%	29%	47%	58%	65%	75%	81%	88%	92%
Comoros	100%	98%	94%	91%	87%	80%	24%	35%	41%
Dem Republic of Congo	99%	26%	42%	51%	58%	68%	74%	82%	87%
Cote dIvoire	99%	8%	41%	53%	61%	72%	78%	86%	90%
Djibouti	100%	86%	7%	30%	37%	48%	57%	67%	75%
Eritrea	100%	79%	32%	41%	49%	60%	69%	78%	84%
Ethiopia	100%	39%	57%	67%	73%	81%	87%	91%	94%
Gambia	100%	95%	90%	84%	79%	27%	34%	44%	53%
Ghana	100%	81%	34%	44%	52%	64%	71%	82%	87%
Guinea	100%	82%	27%	36%	44%	56%	64%	75%	81%
Guinea Bissau	100%	93%	86%	80%	24%	33%	40%	51%	58%
Haiti	100%	96%	90%	84%	79%	29%	36%	45%	52%
India	100%	59%	82%	90%	94%	97%	99%	100%	100%
Kenya	100%	30%	50%	60%	68%	78%	84%	91%	95%
DPR Korea	NA	NA	NA	NA	NA	NA	NA	NA	NA
Kyrgyz Republic	100%	100%	100%	99%	99%	97%	96%	94%	90%
Lao PDR	96%	33%	49%	59%	67%	78%	84%	91%	94%
Lesotho	100%	99%	97%	94%	92%	87%	83%	75%	25%
Liberia	99%	25%	40%	50%	57%	68%	74%	83%	87%
Madagascar	99%	76%	35%	46%	53%	62%	69%	77%	82%
Malawi	100%	98%	96%	93%	90%	83%	78%	28%	35%
Mali	100%	82%	28%	38%	46%	57%	65%	76%	82%
Mauritania	100%	81%	31%	40%	48%	61%	68%	78%	85%
Mozambique	100%	95%	88%	81%	75%	30%	37%	48%	56%
Myanmar	99%	30%	47%	59%	66%	77%	84%	90%	93%
Nepal	100%	80%	28%	38%	46%	57%	65%	75%	81%
Nicaragua	100%	89%	78%	30%	36%	47%	53%	63%	70%
Niger	100%	92%	81%	4%	29%	39%	46%	58%	66%
Nigeria	98%	82%	93%	97%	98%	99%	99%	100%	100%
Pakistan	100%	95%	84%	73%	28%	42%	52%	67%	76%
Papua New Guinea	23%	87%	94%	96%	98%	98%	99%	100%	100%
Rwanda	100%	85%	7%	31%	39%	49%	57%	68%	75%
Sao Tome e Principe	100%	92%	83%	23%	29%	39%	47%	58%	66%
Senegal	100%	95%	87%	79%	72%	37%	45%	58%	68%
Sierra Leone	99%	4%	37%	48%	57%	68%	75%	84%	88%
Solomon Islands	84%	55%	71%	79%	84%	90%	93%	96%	97%
Somalia	100%	84%	29%	38%	47%	58%	65%	75%	82%
Republic of Sudan	100%	99%	96%	94%	92%	86%	82%	74%	26%
South Sudan	100%	35%	53%	64%	71%	81%	86%	92%	95%
Tajikistan	100%	100%	100%	99%	99%	98%	97%	95%	94%
Tanzania	100%	76%	35%	47%	55%	66%	73%	82%	87%
Togo	100%	92%	84%	75%	27%	37%	45%	57%	65%
Uganda	100%	96%	90%	85%	79%	7%	31%	42%	50%
Yemen	72%	63%	76%	82%	86%	90%	93%	95%	96%
Zambia	100%	81%	34%	45%	52%	63%	71%	81%	86%
Zimbabwe	100%	89%	77%	29%	37%	47%	55%	65%	71%

3.4.2 Vaccine cost: including Gavi's share and assuming the delivery cost of campaign doses is the same as routine doses.

Appendix Table 27 and Appendix Table 28 show the optimal intervention strategy for each country, and how certain we are about it, if we include the total vaccine procurement and delivery costs (same as in 3.4.1) and assume the delivery cost of campaign doses to be the same on average as delivery costs for routine doses (shown in Appendix Table 16).

RC15 or RC5 are optimal in 17 countries when the WTP is ≥US$200 per DALY averted, in 34 countries at a WTP ≥US$500 per DALY averted), and in 44 countries at a WTP ≥US$1000 per DALY averted (Appendix Table 27). For three countries (Kyrgyz Republic, Lesotho, and Tajikistan), none of the vaccination programs is optimal at WTP values <US$2000 per DALY averted. In terms of GDP per capita, RC15 or RC5 are optimal in 16 countries at WTP values ≥25% of the GDP per capita, in 32 countries at a WTP ≥50% of the GDP per capita, and in 41 countries at a WTP ≥100% of the GDP per capita (Appendix Table 28).

Appendix Table 27 and Appendix Table 28 indicate that including an initial catch-up campaign is always optimal compared to routine immunisation alone when introducing TCVs. RC15 is consistently optimal compared to RC5 and R for 6 countries. For 45 countries, RC5 is optimal compared to RC15 and R for smaller WTP thresholds; for larger WTP values, RC15 becomes the optimal strategy. For these 45 countries, if RC5 is optimal, the probability that it results in highest net benefit never exceeds 25%.

Appendix Table 27: Cost-effectiveness results when including Gavi's share and assuming the delivery cost of campaign doses is the same as routine doses, for each country for a range of willingness-to-pay values (US$0-US$2000 per DALY averted). The **optimal** strategy (i.e. strategy that yields the highest *average* net monetary benefit) is indicated by the colour-shading: no vaccination (white), routine childhood immunisation (green), routine immunisation with a catch-up campaign up to age 5 years (purple) and routine immunisation with a catch-up campaign up to age 15 years (orange). How certain we are about the **optimal** strategy is indicated by the percentage of parameter samples in which the **optimal** strategy yielded the highest net benefit.

	$0	$100	$200	$300	$400	$500	$600	$700	$800	$900	$1000	$1100	$1200	$1300	$1400	$1500	$1600	$1700	$1800	$1900	$2000
Afghanistan	99%	82%	11%	25%	31%	35%	39%	43%	46%	48%	51%	54%	56%	58%	60%	62%	63%	65%	66%	67%	68%
Bangladesh	96%	10%	26%	32%	37%	42%	45%	49%	51%	53%	55%	58%	59%	61%	62%	64%	65%	66%	68%	69%	70%
Benin	95%	12%	28%	36%	42%	48%	52%	56%	59%	62%	65%	68%	69%	71%	73%	74%	76%	77%	78%	79%	80%
Burkina Faso	100%	84%	10%	10%	31%	35%	40%	44%	47%	51%	54%	57%	59%	62%	64%	66%	68%	69%	71%	72%	73%
Burundi	99%	10%	29%	39%	45%	50%	54%	58%	61%	63%	66%	68%	70%	72%	74%	75%	76%	78%	80%	80%	81%
Cambodia	98%	72%	29%	38%	44%	50%	55%	59%	62%	66%	68%	71%	73%	75%	76%	78%	79%	81%	82%	83%	84%
Cameroon	100%	94%	87%	80%	75%	11%	12%	13%	14%	27%	29%	31%	33%	35%	37%	38%	40%	42%	43%	44%	45%
Central African Republic	100%	86%	75%	9%	29%	34%	38%	42%	45%	48%	50%	53%	55%	57%	59%	61%	64%	65%	67%	68%	70%
Chad	99%	81%	8%	28%	33%	38%	42%	47%	51%	54%	57%	59%	62%	64%	66%	68%	70%	71%	72%	73%	74%
Comoros	100%	100%	98%	96%	94%	92%	90%	88%	86%	84%	83%	80%	79%	77%	76%	75%	74%	7%	8%	7%	7%
Dem Republic of Congo	99%	79%	28%	37%	44%	50%	55%	59%	63%	65%	67%	70%	71%	74%	75%	77%	78%	79%	80%	81%	82%
Cote dIvoire	99%	90%	81%	75%	13%	15%	16%	28%	31%	34%	37%	39%	41%	44%	46%	47%	49%	50%	51%	53%	54%
Djibouti	100%	99%	96%	92%	89%	86%	83%	80%	78%	75%	74%	10%	11%	12%	12%	12%	13%	13%	13%	14%	14%
Eritrea	100%	91%	81%	75%	9%	27%	30%	35%	38%	42%	44%	46%	48%	51%	53%	54%	57%	58%	60%	62%	64%
Ethiopia	100%	75%	28%	38%	44%	50%	55%	59%	61%	64%	66%	68%	71%	72%	74%	75%	76%	78%	79%	80%	82%
Gambia	100%	97%	91%	87%	82%	78%	74%	9%	9%	9%	9%	29%	31%	32%	34%	36%	37%	39%	40%	41%	42%
Ghana	100%	97%	92%	87%	82%	78%	75%	8%	9%	9%	26%	28%	30%	32%	34%	35%	37%	38%	40%	42%	43%
Guinea	100%	87%	76%	8%	28%	33%	37%	40%	43%	47%	49%	52%	54%	56%	58%	60%	62%	64%	66%	67%	68%
Guinea Bissau	100%	98%	93%	89%	85%	82%	78%	76%	7%	8%	8%	26%	27%	29%	31%	33%	35%	36%	37%	39%	40%
Haiti	100%	98%	96%	94%	91%	89%	86%	84%	83%	81%	78%	76%	1%	24%	26%	27%	28%	29%	31%	32%	33%
India	100%	86%	65%	16%	46%	55%	61%	67%	72%	76%	79%	82%	84%	86%	87%	88%	89%	90%	91%	92%	93%
Kenya	100%	91%	83%	74%	9%	29%	34%	38%	41%	44%	47%	49%	52%	54%	57%	59%	61%	63%	64%	66%	67%
DPR Korea	100%	88%	80%	6%	26%	30%	35%	38%	41%	44%	46%	48%	51%	53%	54%	56%	57%	59%	60%	61%	62%
Kyrgyz Republic	100%	100%	100%	100%	100%	100%	100%	100%	99%	99%	99%	99%	99%	99%	98%	98%	98%	98%	98%	97%	97%
Lao PDR	96%	84%	10%	12%	26%	30%	35%	38%	41%	44%	46%	49%	51%	54%	56%	57%	59%	60%	62%	64%	66%
Lesotho	100%	100%	100%	99%	99%	98%	97%	97%	95%	94%	94%	92%	92%	91%	90%	90%	89%	88%	87%	87%	86%
Liberia	99%	79%	28%	36%	44%	50%	55%	59%	63%	65%	67%	69%	71%	73%	75%	76%	78%	79%	80%	81%	82%
Madagascar	99%	78%	27%	36%	43%	48%	52%	56%	60%	63%	65%	67%	69%	71%	73%	74%	76%	77%	78%	79%	80%
Malawi	100%	98%	94%	89%	85%	81%	78%	76%	8%	8%	8%	8%	26%	28%	30%	31%	33%	34%	35%	36%	38%

Mali	100%	92%	83%	77%	8%	9%	27%	30%	33%	36%	39%	42%	44%	46%	48%	50%	52%	53%	55%	56%	58%
Mauritania	100%	96%	88%	82%	77%	73%	8%	8%	27%	30%	32%	34%	36%	38%	40%	42%	43%	45%	47%	48%	49%
Mozambique	100%	95%	88%	81%	76%	9%	9%	10%	28%	31%	33%	35%	37%	39%	42%	44%	45%	46%	48%	49%	51%
Myanmar	100%	87%	76%	9%	28%	33%	37%	40%	44%	47%	50%	53%	55%	57%	59%	61%	63%	65%	66%	68%	69%
Nepal	100%	88%	79%	10%	12%	27%	31%	35%	39%	42%	45%	47%	49%	51%	52%	54%	56%	58%	59%	60%	62%
Nicaragua	100%	99%	97%	94%	92%	90%	88%	86%	84%	83%	81%	80%	78%	77%	76%	3%	3%	25%	26%	27%	28%
Niger	100%	91%	82%	75%	10%	10%	29%	32%	36%	38%	41%	43%	45%	47%	49%	51%	53%	55%	57%	58%	59%
Nigeria	98%	6%	47%	59%	67%	73%	78%	81%	83%	85%	87%	88%	89%	90%	91%	92%	92%	93%	94%	94%	95%
Pakistan	100%	100%	99%	97%	95%	92%	89%	86%	83%	81%	78%	76%	74%	72%	70%	13%	13%	14%	15%	15%	16%
Papua New Guinea	23%	50%	63%	71%	76%	80%	83%	85%	87%	88%	89%	90%	92%	92%	93%	93%	94%	94%	95%	95%	96%
Rwanda	100%	92%	82%	76%	7%	26%	30%	34%	37%	40%	43%	45%	48%	50%	52%	54%	55%	57%	58%	59%	61%
Sao Tome e Principe	100%	99%	98%	96%	93%	91%	88%	86%	84%	82%	80%	78%	77%	75%	74%	6%	6%	6%	7%	6%	26%
Senegal	100%	99%	97%	94%	92%	89%	86%	84%	81%	79%	76%	73%	71%	7%	6%	7%	29%	31%	32%	34%	35%
Sierra Leone	100%	82%	9%	28%	35%	40%	45%	49%	53%	56%	59%	61%	63%	64%	66%	68%	70%	71%	73%	74%	75%
Solomon Islands	82%	16%	32%	40%	46%	51%	55%	58%	62%	64%	67%	69%	71%	73%	74%	76%	77%	78%	79%	80%	81%
Somalia	100%	90%	81%	5%	27%	31%	36%	39%	42%	46%	49%	52%	55%	57%	59%	62%	63%	65%	66%	68%	69%
Republic of Sudan	100%	97%	93%	89%	85%	82%	79%	76%	11%	11%	11%	12%	12%	13%	14%	14%	26%	27%	28%	29%	31%
South Sudan	100%	94%	88%	81%	76%	8%	7%	8%	29%	31%	34%	36%	38%	39%	41%	42%	44%	46%	48%	49%	50%
Tajikistan	100%	100%	100%	100%	100%	100%	100%	100%	99%	99%	99%	99%	99%	99%	98%	98%	98%	98%	98%	97%	97%
Tanzania	100%	90%	81%	74%	9%	27%	31%	35%	38%	41%	44%	46%	49%	51%	53%	55%	57%	59%	60%	62%	63%
Togo	100%	96%	89%	83%	78%	74%	9%	9%	10%	28%	30%	33%	35%	36%	38%	41%	42%	44%	45%	47%	48%
Uganda	100%	98%	94%	90%	86%	83%	79%	76%	75%	9%	9%	9%	10%	11%	11%	28%	29%	30%	31%	32%	34%
Yemen	74%	30%	42%	51%	57%	62%	66%	69%	72%	73%	75%	77%	78%	79%	80%	81%	83%	84%	84%	85%	85%
Zambia	100%	97%	91%	86%	82%	78%	9%	11%	11%	10%	26%	29%	31%	34%	35%	36%	38%	40%	41%	43%	44%
Zimbabwe	100%	97%	91%	86%	82%	78%	74%	8%	9%	26%	29%	31%	33%	35%	37%	39%	40%	42%	43%	45%	46%

Appendix Table 28: Cost-effectiveness results when including Gavi's share and assuming the delivery cost of campaign doses is the same as routine doses, for each country for a range of willingness-to-pay values (0 up to 4 times a country's GDP per capita). The **optimal** strategy (i.e. strategy that yields the highest *average* net monetary benefit) is indicated by the colour-shading: no vaccination (white), routine childhood immunisation (green), routine immunisation with a catch-up campaign up to age 5 years (purple) and routine immunisation with a catch-up campaign up to age 15 years (orange). How certain we are about the **optimal** strategy is indicated by the percentage of parameter samples in which the **optimal** strategy yielded the highest net benefit.

	0	0.25*GDP	0.5*GDP	0.75*GDP	1*GDP	1.5*GDP	2*GDP	3*GDP	4*GDP
Afghanistan	99%	77%	12%	32%	38%	47%	54%	65%	71%
Bangladesh	96%	35%	49%	57%	62%	71%	77%	84%	88%
Benin	95%	28%	41%	52%	58%	69%	75%	82%	87%
Burkina Faso	100%	76%	11%	35%	42%	53%	62%	72%	79%
Burundi	99%	77%	13%	34%	40%	50%	57%	65%	72%
Cambodia	98%	39%	55%	66%	73%	82%	88%	92%	96%
Cameroon	100%	79%	12%	27%	34%	44%	51%	63%	70%
Central African Republic	100%	86%	77%	8%	27%	36%	42%	52%	61%
Chad	99%	9%	35%	44%	52%	64%	71%	80%	85%
Comoros	100%	99%	95%	91%	87%	80%	75%	8%	31%
Dem Republic of Congo	99%	5%	32%	42%	50%	60%	67%	77%	82%
Cote dIvoire	99%	13%	29%	39%	47%	57%	64%	74%	80%
Djibouti	100%	86%	74%	12%	14%	34%	42%	51%	59%
Eritrea	100%	81%	9%	32%	39%	49%	57%	68%	75%
Ethiopia	100%	28%	44%	55%	61%	70%	76%	85%	88%
Gambia	100%	96%	90%	84%	80%	9%	9%	34%	41%
Ghana	100%	83%	9%	29%	36%	48%	56%	66%	74%
Guinea	100%	83%	8%	27%	33%	43%	50%	61%	69%
Guinea Bissau	100%	94%	87%	81%	76%	25%	31%	41%	48%
Haiti	100%	96%	91%	87%	83%	76%	27%	36%	44%
India	100%	49%	75%	86%	90%	96%	98%	99%	100%
Kenya	100%	8%	40%	50%	59%	70%	77%	85%	90%
DPR Korea	NA	NA	NA	NA	NA	NA	NA	NA	NA
Kyrgyz Republic	100%	100%	100%	99%	99%	98%	97%	94%	92%
Lao PDR	96%	30%	45%	57%	64%	75%	81%	89%	93%
Lesotho	100%	99%	98%	95%	92%	88%	84%	77%	10%
Liberia	99%	76%	31%	42%	49%	60%	67%	75%	81%
Madagascar	99%	79%	27%	36%	42%	52%	59%	69%	75%
Malawi	100%	99%	96%	93%	90%	84%	79%	8%	9%
Mali	100%	83%	9%	28%	34%	45%	53%	65%	73%
Mauritania	100%	82%	8%	31%	37%	48%	56%	67%	74%
Mozambique	100%	95%	88%	81%	77%	9%	28%	37%	45%
Myanmar	100%	9%	38%	49%	56%	68%	74%	83%	88%
Nepal	100%	80%	11%	29%	36%	47%	53%	65%	71%
Nicaragua	100%	89%	81%	3%	29%	37%	44%	55%	62%
Niger	100%	91%	82%	75%	10%	30%	36%	46%	54%
Nigeria	98%	76%	88%	93%	96%	98%	99%	100%	100%
Pakistan	100%	96%	85%	76%	13%	17%	37%	52%	63%
Papua New Guinea	23%	84%	92%	95%	97%	98%	99%	99%	100%
Rwanda	100%	83%	7%	28%	34%	45%	53%	63%	70%
Sao Tome e Principe	100%	93%	83%	75%	6%	31%	38%	48%	56%
Senegal	100%	96%	90%	84%	77%	6%	34%	46%	54%
Sierra Leone	100%	75%	28%	38%	45%	57%	63%	74%	80%
Solomon Islands	82%	51%	66%	75%	81%	87%	91%	95%	96%
Somalia	100%	86%	75%	28%	33%	43%	52%	64%	71%
Republic of Sudan	100%	99%	97%	94%	92%	87%	83%	76%	10%
South Sudan	100%	7%	37%	48%	56%	68%	75%	83%	88%
Tajikistan	100%	100%	100%	100%	99%	99%	98%	96%	94%
Tanzania	100%	78%	26%	35%	43%	54%	62%	72%	79%
Togo	100%	93%	84%	76%	8%	28%	34%	45%	53%
Uganda	100%	96%	89%	84%	78%	9%	11%	33%	40%
Yemen	74%	46%	61%	69%	74%	81%	85%	89%	92%

Zambia	100%	85%	9%	26%	33%	43%	51%	62%	70%
Zimbabwe	100%	89%	78%	8%	28%	38%	46%	56%	63%

3.4.3 Typhoid incidence: declining over time without vaccination.

Appendix Table 29 and Appendix Table 30 show the optimal intervention strategy for each country, and how certain we are about it, if we assume the typhoid transmission rate declines at a rate of 25% over 10 years without vaccination (e.g. due to improved sanitation).

RC15 is optimal in 33 countries when the WTP is ≥US$200 per DALY averted, in 46 countries at a WTP ≥US$500 per DALY averted), and in 51 countries at a WTP ≥US$1000 per DALY averted (Appendix Table 29). For three countries (Kyrgyz Republic, Lesotho, and Tajikistan), none of the vaccination programs is optimal at WTP values <US$2000 per DALY averted. In terms of GDP per capita, RC15 is optimal in 35 countries at WTP values ≥25% of the GDP per capita, in 41 countries at a WTP ≥50% of the GDP per capita, and in 48 countries at a WTP ≥100% of the GDP per capita (Appendix Table 30).

In sum, assuming a declining transmission rate over time does not change the results to a great extent. Note that a decrease in transmission rate of 25% did not correspond to a 25% decrease in typhoid incidence. Higher declining rates of transmission are likely to have a greater impact, but it remains difficult to assess the rate at which typhoid incidence is expected to decline over time in the future.

Appendix Table 29: Cost-effectiveness results when assuming decreasing typhoid transmission over time, for each country for a range of willingness-to-pay values (US$0-US$2000 per DALY averted). The **optimal** strategy (i.e. strategy that yields the highest *average* net monetary benefit) is indicated by the colour-shading: no vaccination (white) and routine immunisation with a catch-up campaign up to age 15 years (orange). How certain we are about the **optimal** strategy is indicated by the percentage of parameter samples in which the **optimal** strategy yielded the highest net benefit.

	$0	$100	$200	$300	$400	$500	$600	$700	$800	$900	$1000	$1100	$1200	$1300	$1400	$1500	$1600	$1700	$1800	$1900	$2000
Afghanistan	96%	40%	56%	64%	71%	76%	79%	82%	84%	85%	86%	87%	88%	89%	90%	90%	91%	92%	92%	93%	93%
Bangladesh	91%	41%	53%	60%	66%	70%	73%	75%	78%	80%	82%	83%	85%	86%	87%	89%	89%	90%	91%	91%	92%
Benin	82%	57%	72%	79%	84%	86%	89%	90%	92%	93%	94%	95%	95%	96%	96%	97%	97%	97%	98%	98%	98%
Burkina Faso	96%	35%	52%	62%	69%	75%	79%	82%	84%	86%	88%	89%	90%	91%	92%	93%	93%	94%	94%	95%	95%
Burundi	93%	52%	68%	75%	80%	83%	85%	87%	89%	90%	92%	92%	93%	94%	94%	95%	96%	96%	96%	97%	97%
Cambodia	89%	52%	68%	77%	83%	87%	89%	91%	93%	94%	94%	95%	96%	96%	97%	97%	97%	97%	98%	98%	98%
Cameroon	98%	84%	28%	37%	44%	50%	55%	59%	62%	65%	68%	70%	72%	74%	76%	77%	79%	80%	82%	83%	84%
Central African Republic	95%	37%	53%	62%	69%	74%	77%	80%	82%	84%	86%	87%	89%	90%	91%	92%	92%	92%	93%	93%	94%
Chad	91%	45%	60%	69%	75%	79%	82%	84%	86%	89%	90%	91%	92%	93%	93%	94%	95%	95%	96%	96%	96%
Comoros	100%	97%	93%	88%	84%	80%	75%	27%	30%	33%	36%	38%	40%	43%	44%	46%	48%	49%	51%	53%	54%
Dem Republic of Congo	96%	42%	58%	67%	73%	77%	80%	83%	85%	86%	88%	89%	90%	91%	92%	93%	93%	94%	94%	95%	95%
Cote dIvoire	97%	80%	32%	42%	50%	54%	59%	64%	67%	69%	71%	74%	75%	77%	79%	80%	82%	82%	83%	84%	85%
Djibouti	100%	94%	87%	81%	75%	29%	32%	36%	40%	43%	46%	48%	51%	53%	55%	57%	58%	60%	61%	63%	64%
Eritrea	99%	77%	38%	48%	55%	60%	66%	69%	72%	75%	77%	80%	81%	83%	84%	85%	86%	87%	87%	88%	89%
Ethiopia	98%	49%	66%	74%	80%	83%	86%	88%	89%	91%	92%	93%	94%	94%	95%	96%	96%	97%	97%	97%	98%
Gambia	99%	88%	77%	30%	36%	43%	47%	51%	55%	58%	61%	63%	66%	68%	69%	71%	72%	74%	75%	76%	78%
Ghana	100%	93%	84%	77%	29%	34%	39%	43%	46%	50%	52%	55%	57%	59%	61%	62%	64%	66%	67%	69%	70%
Guinea	97%	34%	50%	60%	68%	72%	76%	79%	82%	84%	86%	87%	88%	89%	90%	92%	93%	93%	94%	94%	94%
Guinea Bissau	100%	90%	82%	26%	32%	37%	41%	46%	49%	52%	54%	57%	59%	61%	63%	65%	66%	68%	70%	71%	72%
Haiti	100%	93%	84%	79%	27%	31%	35%	38%	41%	44%	47%	50%	51%	53%	55%	56%	58%	59%	61%	63%	64%
India	100%	92%	77%	33%	44%	54%	62%	68%	72%	76%	80%	83%	85%	87%	89%	90%	91%	92%	93%	94%	94%
Kenya	99%	83%	30%	41%	49%	54%	59%	63%	67%	70%	73%	75%	77%	79%	81%	83%	84%	85%	86%	87%	88%
DPR Korea	96%	30%	43%	52%	59%	64%	69%	72%	75%	77%	78%	79%	80%	81%	82%	83%	84%	85%	86%	86%	87%
Kyrgyz Republic	100%	100%	100%	100%	99%	99%	99%	98%	98%	98%	97%	96%	96%	96%	95%	95%	95%	94%	94%	93%	93%
Lao PDR	91%	29%	38%	46%	52%	57%	61%	66%	69%	72%	74%	76%	78%	80%	82%	83%	84%	86%	87%	88%	89%
Lesotho	100%	100%	98%	97%	95%	94%	92%	90%	89%	87%	86%	84%	83%	82%	81%	80%	78%	77%	75%	26%	26%
Liberia	95%	42%	58%	67%	74%	78%	81%	84%	86%	88%	90%	91%	91%	92%	93%	94%	94%	95%	95%	96%	96%
Madagascar	93%	47%	63%	71%	77%	81%	84%	86%	88%	89%	90%	91%	92%	93%	93%	94%	94%	94%	95%	95%	96%
Malawi	100%	91%	82%	73%	32%	38%	42%	47%	50%	54%	57%	60%	61%	64%	65%	67%	69%	70%	72%	73%	74%
Mali	99%	77%	37%	47%	54%	60%	65%	68%	72%	75%	76%	79%	80%	83%	84%	85%	86%	87%	88%	90%	90%

Mauritania	99%	86%	74%	34%	41%	47%	52%	56%	60%	62%	66%	68%	70%	72%	73%	75%	77%	78%	80%	81%	82%
Mozambique	99%	85%	29%	38%	45%	51%	56%	60%	64%	67%	69%	71%	74%	76%	77%	79%	81%	82%	83%	85%	86%
Myanmar	98%	73%	42%	52%	59%	65%	70%	74%	77%	79%	81%	84%	86%	87%	88%	89%	90%	91%	91%	92%	92%
Nepal	98%	29%	46%	56%	63%	68%	73%	76%	80%	81%	83%	85%	86%	87%	88%	89%	90%	91%	92%	92%	92%
Nicaragua	100%	97%	94%	90%	87%	84%	82%	79%	23%	25%	27%	30%	32%	33%	34%	36%	37%	38%	39%	41%	42%
Niger	99%	77%	37%	48%	56%	62%	67%	71%	74%	76%	79%	81%	82%	84%	85%	86%	87%	88%	89%	90%	90%
Nigeria	97%	45%	65%	74%	79%	84%	88%	90%	92%	93%	94%	95%	96%	96%	97%	97%	98%	98%	98%	98%	98%
Pakistan	100%	99%	96%	91%	86%	82%	76%	72%	69%	35%	39%	43%	45%	48%	51%	54%	56%	58%	60%	62%	64%
Papua New Guinea	67%	88%	94%	96%	97%	98%	98%	99%	99%	99%	99%	99%	99%	100%	100%	100%	100%	100%	100%	100%	100%
Rwanda	98%	27%	42%	52%	60%	64%	68%	73%	76%	79%	81%	82%	84%	86%	87%	88%	89%	90%	91%	91%	92%
Sao Tome e Principe	100%	98%	96%	93%	90%	87%	84%	81%	79%	77%	26%	28%	30%	32%	33%	35%	37%	38%	40%	41%	43%
Senegal	100%	95%	88%	83%	78%	73%	31%	36%	41%	44%	46%	49%	52%	54%	56%	58%	60%	61%	63%	64%	65%
Sierra Leone	96%	38%	55%	64%	71%	77%	80%	82%	85%	86%	89%	90%	91%	91%	92%	92%	93%	94%	94%	95%	95%
Solomon Islands	74%	52%	64%	71%	76%	80%	83%	85%	86%	88%	89%	90%	91%	91%	92%	93%	93%	93%	94%	94%	95%
Somalia	98%	27%	44%	53%	60%	66%	72%	75%	78%	80%	82%	84%	86%	87%	88%	89%	90%	91%	92%	92%	92%
Republic of Sudan	100%	94%	88%	82%	78%	27%	31%	35%	38%	41%	44%	46%	48%	50%	52%	54%	56%	58%	59%	60%	62%
South Sudan	96%	75%	38%	48%	54%	60%	65%	68%	72%	74%	76%	79%	80%	82%	84%	85%	86%	87%	88%	88%	89%
Tajikistan	100%	100%	100%	100%	99%	99%	99%	98%	98%	98%	97%	97%	97%	96%	96%	95%	95%	94%	94%	94%	93%
Tanzania	100%	79%	36%	45%	53%	59%	65%	69%	72%	75%	77%	79%	81%	82%	83%	84%	85%	86%	87%	88%	88%
Togo	100%	86%	73%	35%	42%	48%	53%	58%	61%	65%	67%	70%	71%	73%	75%	76%	78%	80%	81%	82%	83%
Uganda	100%	92%	84%	75%	30%	35%	40%	44%	47%	51%	54%	57%	60%	62%	63%	65%	66%	68%	70%	71%	72%
Yemen	51%	78%	85%	89%	92%	93%	94%	95%	96%	96%	96%	97%	97%	97%	98%	98%	98%	98%	98%	98%	98%
Zambia	100%	91%	83%	26%	32%	37%	42%	46%	49%	51%	54%	57%	59%	61%	63%	65%	67%	68%	70%	71%	72%
Zimbabwe	100%	89%	78%	30%	36%	43%	47%	50%	54%	57%	60%	62%	64%	66%	67%	69%	70%	71%	73%	74%	75%

Appendix Table 30: Cost-effectiveness results when assuming decreasing typhoid transmission over time, for each country for a range of willingness-to-pay values (0 up to 4 times a country's GDP per capita). The **optimal** strategy (i.e. strategy that yields the highest *average* net monetary benefit) is indicated by the colour-shading: no vaccination (white) and routine immunisation with a catch-up campaign up to age 15 years (orange). How certain we are about the **optimal** strategy is indicated by the percentage of parameter samples in which the **optimal** strategy yielded the highest net benefit.

	0	0.25*GDP	0.5*GDP	0.75*GDP	1*GDP	1.5*GDP	2*GDP	3*GDP	4*GDP
Afghanistan	96%	47%	63%	72%	78%	84%	88%	92%	94%
Bangladesh	91%	63%	76%	83%	88%	92%	95%	98%	98%
Benin	82%	71%	83%	88%	92%	95%	97%	98%	99%
Burkina Faso	96%	47%	63%	74%	80%	88%	91%	95%	97%
Burundi	93%	48%	63%	71%	76%	83%	86%	92%	94%
Cambodia	89%	77%	90%	94%	96%	98%	99%	100%	100%
Cameroon	98%	38%	56%	66%	73%	82%	87%	92%	95%
Central African Republic	95%	36%	51%	60%	67%	76%	81%	87%	91%
Chad	91%	62%	76%	83%	87%	93%	95%	97%	98%
Comoros	100%	93%	85%	77%	29%	39%	46%	58%	66%
Dem Republic of Congo	96%	47%	63%	71%	77%	84%	87%	92%	95%
Cote dIvoire	97%	47%	65%	73%	80%	86%	91%	95%	97%
Djibouti	100%	28%	44%	55%	63%	72%	80%	87%	92%
Eritrea	99%	38%	56%	66%	72%	82%	86%	92%	94%
Ethiopia	98%	66%	80%	86%	89%	94%	96%	98%	99%
Gambia	99%	86%	74%	33%	41%	52%	59%	70%	76%
Ghana	100%	29%	45%	56%	64%	75%	81%	89%	93%
Guinea	97%	39%	56%	66%	73%	81%	86%	92%	94%
Guinea Bissau	100%	83%	29%	38%	45%	56%	63%	73%	79%
Haiti	100%	85%	74%	34%	40%	51%	57%	67%	72%
India	100%	47%	75%	87%	92%	97%	98%	100%	100%
Kenya	99%	47%	65%	76%	83%	90%	93%	96%	98%
DPR Korea	NA	NA	NA	NA	NA	NA	NA	NA	NA
Kyrgyz Republic	100%	100%	99%	98%	96%	94%	93%	88%	84%
Lao PDR	91%	56%	73%	82%	88%	93%	96%	98%	99%
Lesotho	100%	97%	92%	87%	84%	76%	30%	40%	47%
Liberia	95%	46%	62%	71%	77%	84%	89%	94%	96%
Madagascar	93%	47%	62%	71%	77%	84%	87%	92%	94%
Malawi	100%	94%	86%	80%	74%	35%	42%	53%	61%
Mali	99%	38%	55%	66%	72%	81%	87%	92%	94%
Mauritania	99%	35%	52%	64%	71%	81%	86%	92%	94%
Mozambique	99%	85%	28%	37%	44%	55%	63%	73%	80%
Myanmar	98%	53%	72%	80%	86%	92%	94%	97%	99%
Nepal	98%	43%	61%	71%	77%	85%	89%	94%	96%
Nicaragua	100%	83%	29%	37%	43%	53%	61%	69%	76%
Niger	99%	76%	38%	49%	57%	68%	74%	83%	88%
Nigeria	97%	86%	95%	98%	99%	99%	100%	100%	100%
Pakistan	100%	88%	72%	43%	53%	68%	78%	88%	92%
Papua New Guinea	67%	98%	99%	100%	100%	100%	100%	100%	100%
Rwanda	98%	40%	57%	66%	74%	82%	88%	93%	95%
Sao Tome e Principe	100%	90%	78%	31%	38%	50%	57%	68%	74%
Senegal	100%	86%	73%	37%	45%	57%	64%	74%	82%
Sierra Leone	96%	48%	65%	75%	81%	87%	91%	94%	96%
Solomon Islands	74%	80%	89%	92%	95%	97%	98%	99%	100%
Somalia	98%	34%	50%	61%	70%	78%	84%	91%	93%
Republic of Sudan	100%	97%	93%	89%	85%	80%	25%	35%	42%
South Sudan	96%	65%	80%	88%	91%	95%	97%	99%	100%
Tajikistan	100%	100%	99%	99%	98%	97%	95%	92%	89%
Tanzania	100%	41%	58%	70%	76%	84%	88%	93%	96%
Togo	100%	79%	35%	45%	53%	64%	71%	80%	86%
Uganda	100%	87%	74%	34%	42%	52%	61%	71%	78%
Yemen	51%	86%	93%	95%	96%	98%	98%	99%	100%
Zambia	100%	27%	43%	53%	61%	71%	78%	86%	90%
Zimbabwe	100%	74%	42%	52%	60%	68%	74%	81%	85%

3.4.4 Time horizon: 30 years

Appendix Table 31 and Appendix Table 32 show the optimal intervention strategy, and how certain we are about it, if we include the costs and health benefits for 30 years (instead of 10 years as we did in our primary analysis). Results are only shown for the countries for which we could derive the Gavi contribution for 2029-2048, i.e. the 17 countries assumed to not receive any additional support for 2029-2048.

The time horizon for the analysis impacts the results in a different way for each country. For 7 of the 17 countries, a longer time horizon results in a similar or lower minimum WTP value at which vaccination becomes optimal (Appendix Table 31). For 10 of the 17 countries, a longer time horizon results in a higher minimum WTP value at which vaccination becomes optimal (Appendix Table 31). For countries for which we assumed a large Gavi contribution in the first 10 years and little or no support over the next 20 years, the results may become less in favour of vaccination when using a 30-year instead of a 10-year time horizon. Also, for some countries (especially the ones with a relatively high baseline incidence), typhoid incidence is predicted to rebound over time as vaccine-induced immunity wanes, which may also explain the less favourable results for 3 countries if using a longer time horizon.

Appendix Table 31:Cost-effectiveness results when including costs and health benefits for 30 years, for a range of willingness-to-pay values (US$0-US$2000 per DALY averted). The **optimal** strategy (i.e. strategy that yields the highest *average* net monetary benefit) is indicated by the colour-shading: no vaccination (white) and routine immunisation with a catch-up campaign up to age 15 years (orange). How certain we are about the **optimal** strategy is indicated by the percentage of parameter samples in which the **optimal** strategy yielded the highest net benefit. Results are only shown for the countries for which we could derive the Gavi contribution for 2029-2048, i.e. the countries assumed to not receive any additional support for 2029-2048.

	$0	$100	$200	$300	$400	$500	$600	$700	$800	$900	$1000	$1100	$1200	$1300	$1400	$1500	$1600	$1700	$1800	$1900	$2000
Bangladesh	92%	36%	48%	54%	59%	64%	68%	71%	73%	76%	78%	79%	81%	82%	83%	84%	85%	85%	86%	87%	88%
Cambodia	92%	43%	59%	68%	74%	79%	83%	85%	87%	88%	89%	90%	91%	92%	93%	94%	94%	95%	95%	96%	96%
Cote dIvoire	98%	85%	75%	35%	41%	48%	52%	57%	61%	64%	67%	69%	71%	73%	74%	76%	77%	78%	79%	81%	82%
Ghana	100%	93%	84%	77%	29%	34%	39%	43%	47%	49%	52%	55%	58%	60%	63%	64%	66%	67%	69%	71%	72%
India	99%	79%	32%	46%	58%	65%	71%	76%	79%	82%	85%	86%	88%	90%	91%	92%	93%	94%	94%	95%	96%
Kenya	99%	85%	28%	39%	46%	53%	58%	62%	66%	68%	71%	73%	75%	77%	78%	80%	81%	82%	83%	84%	85%
Lao PDR	92%	73%	39%	46%	52%	57%	61%	66%	69%	71%	74%	76%	78%	80%	81%	83%	84%	85%	86%	87%	87%
Lesotho	100%	100%	99%	98%	96%	95%	94%	93%	91%	90%	89%	87%	86%	85%	84%	83%	82%	81%	80%	78%	77%
Myanmar	98%	77%	37%	46%	54%	60%	64%	68%	71%	74%	77%	78%	80%	81%	83%	84%	85%	86%	87%	88%	88%
Nicaragua	100%	97%	94%	90%	86%	83%	79%	77%	26%	28%	29%	32%	33%	35%	37%	39%	40%	42%	43%	45%	46%
Nigeria	94%	52%	70%	79%	84%	87%	90%	92%	93%	95%	96%	96%	97%	97%	97%	98%	98%	98%	98%	98%	98%
Pakistan	100%	99%	98%	95%	90%	86%	82%	79%	75%	71%	67%	36%	40%	42%	45%	48%	50%	52%	54%	56%	57%
Papua New Guinea	60%	83%	89%	92%	94%	95%	96%	97%	98%	98%	99%	99%	99%	99%	99%	99%	99%	99%	99%	99%	99%
Republic of Sudan	100%	94%	88%	83%	78%	27%	30%	34%	36%	40%	42%	45%	46%	49%	50%	52%	53%	55%	56%	58%	59%
Sao Tome e Principe	100%	98%	95%	92%	88%	85%	82%	78%	75%	27%	30%	32%	34%	36%	38%	40%	41%	42%	44%	45%	47%
Solomon Islands	73%	53%	65%	72%	76%	79%	81%	84%	86%	87%	89%	89%	90%	91%	92%	93%	93%	94%	94%	94%	95%
Zambia	100%	92%	82%	74%	32%	37%	41%	45%	49%	52%	54%	57%	59%	62%	64%	66%	67%	69%	70%	72%	73%

Appendix Table 32: Cost-effectiveness results when including costs and health benefits for 30 years, for a range of willingness-to-pay values (0 up to 4 times a country's GDP per capita). The **optimal** strategy (i.e. strategy that yields the highest *average* net monetary benefit) is indicated by the colour-shading: no vaccination (white) and routine immunisation with a catch-up campaign up to age 15 years (orange). How certain we are about the **optimal** strategy is indicated by the percentage of parameter samples in which the **optimal** strategy yielded the highest net benefit. Results are only shown for the countries for which we could derive the Gavi contribution for 2029-2048, i.e. the countries assumed to not receive any additional support for 2029-2048.

	0	0.25*GDP	0.5*GDP	0.75*GDP	1*GDP	1.5*GDP	2*GDP	3*GDP	4*GDP
Bangladesh	92%	57%	71%	78%	83%	88%	92%	95%	97%
Cambodia	92%	69%	83%	88%	92%	95%	97%	98%	99%
Cote dIvoire	98%	39%	58%	69%	75%	83%	88%	93%	95%
Ghana	100%	29%	46%	57%	65%	76%	82%	89%	92%
India	99%	60%	81%	89%	94%	97%	98%	99%	100%
Kenya	99%	45%	65%	74%	80%	87%	92%	96%	97%
Lao PDR	92%	57%	73%	82%	87%	92%	95%	98%	99%
Lesotho	100%	98%	94%	90%	87%	80%	26%	35%	41%
Myanmar	98%	48%	65%	76%	81%	88%	91%	95%	97%
Nicaragua	100%	82%	31%	40%	48%	57%	64%	72%	78%
Nigeria	94%	89%	96%	98%	99%	100%	100%	100%	100%
Pakistan	100%	92%	77%	36%	47%	61%	71%	82%	88%
Papua New Guinea	60%	97%	99%	99%	100%	100%	100%	100%	100%
Republic of Sudan	100%	97%	93%	90%	86%	80%	74%	34%	41%
Sao Tome e Principe	100%	87%	74%	36%	42%	53%	61%	71%	79%
Solomon Islands	73%	79%	88%	92%	95%	97%	98%	99%	100%
Zambia	100%	27%	43%	53%	61%	72%	79%	86%	90%

3.4.5 Case fatality rate in hospital for Nigeria and Ethiopia.

Studies conducted within Nigeria and Ethiopia estimated an extremely high CFR_{hosp} (≥15%). If we assume the common estimated CFR_{hosp} (on average 5·9%) for these countries instead of the country-specific CFR_{hosp}, the minimum WTP value at which vaccination becomes optimal remains the same (i.e. at 25% GDP per capita per DALY averted), but the probability that it results in highest net benefit decreases to 72% and 45% (Nigeria and Ethiopia respectively).

4 Interpretation of results for policy-making

This section describes how the results from our analysis can/should be used to make decisions on a country-level about TCV implementation and/or whether to embark on obtaining more evidence on uncertain aspects of typhoid fever.

What is the optimal strategy in your country?

Routine immunisation including a catch-campaign is always optimal when compared to routine immunisation alone. Whether routine immunisation including a catch-campaign is optimal compared to no vaccination in a given country depends on the 'willingness-to-pay (WTP) threshold', i.e. what a decision-maker is willing to pay for a health gain (e.g. for one averted disability-adjusted life-year). Healthcare technologies are considered to be relatively cost-effective if their additional cost per unit health gain lies below the WTP threshold defined by decision-makers or—equivalently—if the strategy results in a net monetary benefit (and not a loss) when using the decision-maker's WTP threshold. Consistent use of WTP thresholds can improve decision-making, as it sets a standard that allows optimizing the total sum of achievable health gains under a budget restriction. There is no 'one size fits all' WTP threshold for all countries. Each country is encouraged to establish their own WTP threshold, reflecting local preferences, rather than using for instance 'one time GDP per capita and three times GDP per capita per DALY averted' as thresholds to classify healthcare technologies as 'very cost-effective' and 'cost effective', respectively.[67, 68] The choice of the WTP threshold is related to budget restrictions and local value judgments on efficiency-equity trade-offs, and may be guided by the cost-effectiveness of previously funded and non-funded healthcare technology to stimulate consistency in policy-making. For instance, a strategy that achieves an additional cost per unit health gain below that of other widely implemented, non-controversial strategies might be considered a candidate for coverage based on its comparative cost-effectiveness.

In this article, we take the approach to present results for a range of WTP values expressed in absolute terms as well as a % of GDP per capita. If you specified your WTP value in absolute terms, see Table 3 of the main text of the manuscript; if you specified it in terms of your country's GDP per capita, see Table 4. In Tables 3 and 4, the colour of the cell that corresponds with your country and your WTP value denotes the most cost-effective strategy for your country (white = no vaccination, orange = RC15) given: 1) current evidence and 2) your country's contribution to vaccine price, which depends on obtaining Gavi support and your assumed transition phase from Gavi support for the next 10 years. If you want to make a decision today about TCV implementation based on cost-effectiveness alone, this should be your preferred strategy, irrespective of our uncertainty about various aspects of the disease and its burden.

Is additional evidence valuable?

If you specified your WTP value in absolute terms, see Table 3 of the manuscript; if you specified it in terms of your country's GDP per capita, see Table 4. The number in the cell that corresponds with your country and your WTP value denotes our certainty of the optimal strategy in terms of cost-effectiveness in your country, based on the available evidence. A high percentage (e.g. 95%) indicates that we are quite certain what the optimal strategy is in your country. A rather low percentage (e.g. 30%) indicates that there is a lot of uncertainty around the optimal strategy in your country. The latter does not mean that a decision should not be made, but it indicates that investing in obtaining more evidence may be valuable for future decision-making. The next section indicates which information may be most valuable for your country.

What additional evidence is most valuable?

By 'most valuable evidence', we refer to the evidence that will result in the greatest reduction in the uncertainty about the optimal strategy (in terms of cost-effectiveness). This is based on the results of the EVPPI analysis (Appendix 3.3.2). For India, more precise estimates of typhoid incidence are most valuable; for Pakistan, more precise estimates of the typhoid mortality rate among hospitalised patients is most valuable; for all other 52 Gavi-eligible countries, more precise estimates of the probability of hospitalisation for typhoid are most valuable.

What is the minimum typhoid hospitalisation, incidence and/or mortality rate necessary for vaccination to become optimal?

Once you know the uncertain characteristic(s) for which additional evidence is most valuable for your country (see previous section), Appendix Table 22, Appendix Table 23 and

Appendix Table 24 list the minimum value for that characteristic for which TCV is considered to be optimal in terms of cost-effectiveness. For example, if more evidence about probability of hospitalisation for typhoid is most valuable in your country, consider Appendix Table 22. The value in the cell that corresponds with your country and your WTP value indicates the minimum typhoid hospitalisation rate for TCV vaccination to be optimal in your country. If either a 'no' or 'RC15' is listed in the cell, this means that the uncertainty around the typhoid hospitalisation rate does not influence the optimal strategy for your country and your chosen WTP value.

5 Discussion

5.1 Comparison with previous health economic evaluations

For the most part, the results of our analysis agree with the two previously published economic evaluations of TCVs. In particular, all of the analyses have found that vaccination using TCVs is likely to be cost-effective when the incidence of typhoid fever is high (>100 cases per 100,000 person-years), and identified the probability of hospitalisation and/or case fatality rate as a key source of uncertainty. However, unlike us, Lo et al[61] found that routine vaccination alone was a more cost-effective strategy in medium-incidence settings (50-130 cases per 100,000 person-years). Here, we provide further discussion of the potential explanations underlying this discrepancy.

First, Lo et al[61] did not compare routine vaccination plus a catch-up campaign incrementally to routine vaccination alone, and hence a direct preference for one strategy over the other cannot be obtained from their analysis. Second, we used a lower vaccine delivery cost for campaigns relative to routine immunisation (~US$0·40 for campaign doses versus ~US$1·50 for routine doses),[58] whereas Lo et al[61] assumed a similar cost of delivery for routine and campaign doses. The delivery cost of TCV vaccines are currently unknown and need to be derived from other studies. Country multi-year plans may overestimate delivery costs; therefore, we only included data from studies that measured the costs in the field (sections 2.2.6.3 and 2.2.6.4). However, these costs may be an underestimate if delivery costs increase more rapidly than the inflation rate. Nevertheless, if we assume the vaccine delivery cost per dose for campaigns to on average equal to that for routine immunisation, routine vaccination including a catch-up campaign remains the optimal vaccine delivery strategy in our analysis (Appendix Table 27 andAppendix Table 28). Third, in their primary analysis, Lo et al[61] only considered a catch-up campaign among school-aged children (5-14 year olds). In a scenario analysis, they found that routine vaccination including a catch-up campaign among all children <15 years old dominated the strategy including only school-aged children. Fourth and perhaps most importantly, Lo et al[61] assumed a similar proportion of cases among <5 year olds and 5-9 year olds in medium-incidence settings, based on an age distribution that was interpolated from high- and low-incidence settings.[69] However, we found that in most medium-incidence settings, fewer cases occurred among <5 year olds,[3] and therefore we estimated a lower relative risk for <2 year olds and 2-5 year olds to capture the lower rates of disease observed in these age groups. Thus, Lo et al[61] may have overestimated typhoid incidence in <5 year olds, particularly in medium-incidence settings. This would make routine vaccination more attractive.

6 CHEERS Checklist

Items to include when reporting economic evaluations of health interventions:[70]

Section	Item No	Recommendation	Reported on page No/line No
Title and Abstract			
Title	1	Identify the study as an economic evaluation or use more specific terms such as “cost-effectiveness analysis”, and describe the interventions compared.	Title 'Cost-effectiveness'
Abstract	2	Provide a structured summary of objectives, perspective, setting, methods (including study design and inputs), results (including base case and uncertainty analyses), and conclusions.	Abstract
Introduction			
Background and objectives	3	Provide an explicit statement of the broader context for the study. Present the study question and its relevance for health policy or practice decisions.	Introduction: first two paragraphs; last paragraph
Methods			
Target population and subgroups	4	Describe characteristics of the base case population and subgroups analysed, including why they were chosen.	1st sentence of 'overview and cost-effectiveness framework'
Setting and location	5	State relevant aspects of the system(s) in which the decision(s) need(s) to be made.	1st sentence of 'overview and cost-effectiveness framework'
Study perspective	6	Describe the perspective of the study and relate this to the costs being evaluated.	Methods, 2nd paragraph:' We conducted our economic evaluation from the healthcare provider perspective, including only direct medical costs.'
Comparators	7	Describe the interventions or strategies being compared and state why they were chosen.	methods, 1st paragraph
Time horizon	8	State the time horizon(s) over which costs and consequences are being evaluated and say why appropriate.	methods, 1st paragraph
Discount rate	9	Report the choice of discount rate(s) used for costs and outcomes and say why appropriate.	methods, 2nd paragraph
Choice of health outcomes	10	Describe what outcomes were used as the measure(s) of benefit in the evaluation and their relevance for the type of analysis performed.	methods, 2nd paragraph
Measurement of effectiveness	11a	*Single study-based estimates*: Describe fully the design features of the single effectiveness study and why the single study was a sufficient source of clinical effectiveness data.	Not applicable
	11b	*Synthesis-based estimates*: Describe fully the methods used for identification of included studies and synthesis of clinical effectiveness data.	Appendix 2.1.3.2
Measurement and valuation of preference	12	If applicable, describe the population and methods used to elicit preferences for outcomes.	Not applicable

based outcomes			
Estimating resources and costs	13a	*Single study-based economic evaluation*: Describe approaches used to estimate resource use associated with the alternative interventions. Describe primary or secondary research methods for valuing each resource item in terms of its unit cost. Describe any adjustments made to approximate to opportunity costs.	Not applicable
	13b	*Model-based economic evaluation*: Describe approaches and data sources used to estimate resource use associated with model health states. Describe primary or secondary research methods for valuing each resource item in terms of its unit cost. Describe any adjustments made to approximate to opportunity costs.	Appendix 2.2.5
Currency, price date, and conversion	14	Report the dates of the estimated resource quantities and unit costs. Describe methods for adjusting estimated unit costs to the year of reported costs if necessary. Describe methods for converting costs into a common currency base and the exchange rate.	Methods, 2nd paragraph + Appendix 2.2.5
Choice of model	15	Describe and give reasons for the specific type of decision-analytical model used. Providing a figure to show model structure is strongly recommended.	Appendix 2.1.1.1 and Appendix Figure 1
Assumptions	16	Describe all structural or other assumptions underpinning the decision-analytical model.	Appendix 2.1
Analytical methods	17	Describe all analytical methods supporting the evaluation. This could include methods for dealing with skewed, missing, or censored data; extrapolation methods; methods for pooling data; approaches to validate or make adjustments (such as half cycle corrections) to a model; and methods for handling population heterogeneity and uncertainty.	Methods section + Appendix 2.1 and 2.2
Results			
Study parameters	18	Report the values, ranges, references, and, if used, probability distributions for all parameters. Report reasons or sources for distributions used to represent uncertainty where appropriate. Providing a table to show the input values is strongly recommended.	Table 1 and 2 + Appendix 2.1 and 2.2
Incremental costs and outcomes	19	For each intervention, report mean values for the main categories of estimated costs and outcomes of interest, as well as mean differences between the comparator groups. If applicable, report incremental cost-effectiveness ratios.	First two paragraphs of Results, Appendix 3.1 and Appendix Tables 18-20
Characterizing uncertainty	20a	*Single study-based economic evaluation*: Describe the effects of sampling uncertainty for the estimated incremental cost and incremental effectiveness parameters, together with the impact of methodological assumptions (such as discount rate, study perspective).	Not applicable.
	20b	*Model-based economic evaluation*: Describe the effects on the results of uncertainty for all input parameters, and uncertainty related to the structure of the model and assumptions.	Results, 3 last paragraphs
Characterizing heterogeneity	21	If applicable, report differences in costs, outcomes, or cost-effectiveness that can be explained by variations between subgroups of patients with different baseline characteristics or other observed variability in effects that are not reducible by more information.	Results 'What makes vaccination optimal in some countries but not in others?'
Discussion			
Study findings, limitations, generalizability, and current knowledge	22	Summarise key study findings and describe how they support the conclusions reached. Discuss limitations and the generalisability of the findings and how the findings fit with current knowledge.	Discussion section
Other			
Source of funding	23	Describe how the study was funded and the role of the funder in the identification, design, conduct, and reporting of the analysis. Describe other non-monetary sources of support.	last sentence of Abstract + 'Role of the

			funding sources' in Methods section
Conflicts of interest	24	Describe any potential for conflict of interest of study contributors in accordance with journal policy. In the absence of a journal policy, we recommend authors comply with International Committee of Medical Journal Editors recommendations.	'Declaration of interests' at the end of the main text

References

1. Pitzer VE, Bowles CC, Baker S, et al. Predicting the impact of vaccination on the transmission dynamics of typhoid in South Asia: a mathematical modeling study. *PLoS neglected tropical diseases* 2014; **8**(1): e2642.
2. Antillon M, Bilcke J, Paltiel AD, Pitzer VE. Cost-effectiveness analysis of typhoid conjugate vaccines in five endemic low- and middle-income settings. *Vaccine* 2017; **35**(27): 3506-14.
3. Britto C, Pollard AJ, Voysey M, Blohmke CJ. An Appraisal of the Clinical Features of Pediatric Enteric Fever: Systematic Review and Meta-analysis of the Age-Stratified Disease Occurrence. *Clinical Infectious Diseases* 2017; **64**(11): 1604-11.
4. Cho JC, Kim SJ. Viable, but non-culturable, state of a green fluorescence protein-tagged environmental isolate of Salmonella typhi in groundwater and pond water. *Fems Microbiology Letters* 1999; **170**(1): 257-64.
5. Lanh MN, Bay PV, Ho VA, et al. Persistent efficacy of Vi conjugate vaccine against typhoid fever in young children. *New England Journal of Medicine* 2003; **349**(14): 1390-1.
6. Voysey M, Pollard AJ. Sero-efficacy of Vi-polysaccharide tetanus-toxoid typhoid conjugate vaccine (Typbar-TCV). *Clin Infect Dis* 2018.
7. Szu SC, Klugman KP, Hunt S. Re-examination of immune response and estimation of anti-Vi IgG protective threshold against typhoid fever-based on the efficacy trial of Vi conjugate in young children. *Vaccine* 2014; **32**(20): 2359-63.
8. UN World Population Prospects. https://esa.un.org/unpd/wpp/DataQuery/
9. Antillon M, Warren JL, Crawford FW, et al. The burden of typhoid fever in low- and middle-income countries: A meta-regression approach. *PLoS Negl Trop Dis* 2017; **11**(2): e0005376.
10. Antillon M, Warren JL, Crawford FW, et al. The burden of typhoid fever in low- and middle-income countries: A meta-regression approach. *PLoS neglected tropical diseases* 2017; **11**(2).
11. Mogasale V, Maskery B, Ochiai RL, et al. Burden of typhoid fever in low-income and middle-income countries: a systematic, literature-based update with risk-factor adjustment. *The Lancet Global health* 2014; **2**(10): e570-80.
12. Ochiai RL, Acosta CJ, Danovaro-Holliday MC, et al. A study of typhoid fever in five Asian countries: disease burden and implications for controls. *Bull World Health Organ* 2008; **86**(4): 260-8.
13. Marks F, von Kalckreuth V, Aaby P, et al. Incidence of invasive salmonella disease in sub-Saharan Africa: a multicentre population-based surveillance study. *Lancet Global Health* 2017; **5**(3): E310-E23.
14. Vos T, Allen C, Arora M, et al. Global, regional, and national incidence, prevalence, and years lived with disability for 310 diseases and injuries, 1990-2015: a systematic analysis for the Global Burden of Disease Study 2015. *Lancet* 2016; **388**(10053): 1545-602.
15. WHO. Typhoid vaccines: WHO position paper – March 2018. 2018. http://apps.who.int/iris/bitstream/handle/10665/272272/WER9313.pdf?ua=1 (accessed June 1st 2018).
16. Jin C, Gibani MM, Moore M, et al. Efficacy and immunogenicity of a Vi-tetanus toxoid conjugate vaccine in the prevention of typhoid fever using a controlled human

infection model of Salmonella Typhi: a randomised controlled, phase 2b trial. *Lancet* 2017; **390**(10111): 2472-80.
17. Mohan VK, Varanasi V, Singh A, et al. Safety and Immunogenicity of a Vi Polysaccharide-Tetanus Toxoid Conjugate Vaccine (Typbar-TCV) in Healthy Infants, Children, and Adults in Typhoid Endemic Areas: A Multicenter, 2-Cohort, Open-Label, Double-Blind, Randomized Controlled Phase 3 Study. *Clinical Infectious Diseases* 2015; **61**(3): 393-402.
18. Meiring JE, Gibani M, Basnyat B, et al. The Typhoid Vaccine Acceleration Consortium (TyVAC): Vaccine effectiveness study designs: Accelerating the introduction of typhoid conjugate vaccines and reducing the global burden of enteric fever. Report from a meeting held on 26-27 October 2016, Oxford, UK. *Vaccine* 2017; **35**(38): 5081-8.
19. Collaborators GBDCoD. Global, regional, and national age-sex specific mortality for 264 causes of death, 1980-2016: a systematic analysis for the Global Burden of Disease Study 2016. *Lancet* 2017; **390**(10100): 1151-210.
20. Pieters Z, Saad NJ, Antillon M, Pitzer VE, Bilcke J. Case fatality rate of enteric fever in endemic countries: A systematic review and meta-analysis. *Clin Infect Dis* 2018.
21. Panzner U, Pak GD, Aaby P, et al. Utilization of Healthcare in the Typhoid Fever Surveillance in Africa Program. *Clinical Infectious Diseases* 2016; **62**: S56-S68.
22. Kaljee LM, Pach A, Thriemer K, et al. Utilization and Accessibility of Healthcare on Pemba Island, Tanzania: Implications for Health Outcomes and Disease Surveillance for Typhoid Fever. *American Journal of Tropical Medicine and Hygiene* 2013; **88**(1): 144-52.
23. Luby SP, Saha S, Andrews JR. Towards sustainable public health surveillance for enteric fever. *Vaccine* 2015; **33**: C3-C7.
24. Mogasale V, Mogasale VV, Ramani E, et al. Revisiting typhoid fever surveillance in low and middle income countries: lessons from systematic literature review of population-based longitudinal studies. *Bmc Infectious Diseases* 2016; **16**.
25. Abboud L. Global hospitalization rate of typhoid fever: a meta-regression. Antwerp: University of Antwerp; 2017.
26. Brooks WA, Hossain A, Goswami D, et al. Bacteremic typhoid fever in children in an urban slum, Bangladesh. *Emerging infectious diseases* 2005; **11**(2): 326-9.
27. Naheed A, Ram PK, Brooks WA, et al. Burden of typhoid and paratyphoid fever in a densely populated urban community, Dhaka, Bangladesh. *International journal of infectious diseases : IJID : official publication of the International Society for Infectious Diseases* 2010; **14 Suppl 3**: e93-9.
28. Ochiai RL, Acosta CJ, Danovaro-Holliday MC, et al. A study of typhoid fever in five Asian countries: disease burden and implications for controls. *Bull World Health Organ* 2008; **86**(4): 260-8.
29. Crump JA, Youssef FG, Luby SP, et al. Estimating the incidence of typhoid fever and other febrile illnesses in developing countries. *Emerging infectious diseases* 2003; **9**(5): 539-44.
30. Srikantiah P, Girgis FY, Luby SP, et al. Population-based surveillance of typhoid fever in Egypt. *The American journal of tropical medicine and hygiene* 2006; **74**(1): 114-9.
31. Punjabi NH, Agtini MD, Ochiai RL, et al. Enteric fever burden in North Jakarta, Indonesia: a prospective, community-based study. *Journal of infection in developing countries* 2013; **7**(11): 781-7.

32. Sinha A, Sazawal S, Kumar R, et al. Typhoid fever in children aged less than 5 years. *Lancet (London, England)* 1999; **354**(9180): 734-7.
33. Bahl R, Sinha A, Poulos C, et al. Costs of illness due to typhoid fever in an Indian urban slum community: implications for vaccination policy. *J Health Popul Nutr* 2004; **22**(3): 304-10.
34. Sur D, von Seidlein L, Manna B, et al. The malaria and typhoid fever burden in the slums of Kolkata, India: data from a prospective community-based study. *Transactions of the Royal Society of Tropical Medicine and Hygiene* 2006; **100**(8): 725-33.
35. Breiman RF, Cosmas L, Njuguna H, et al. Population-based incidence of typhoid fever in an urban informal settlement and a rural area in Kenya: implications for typhoid vaccine use in Africa. *PloS one* 2012; **7**(1): e29119.
36. Owais A, Sultana S, Zaman U, Rizvi A, Zaidi AK. Incidence of typhoid bacteremia in infants and young children in southern coastal Pakistan. *The Pediatric infectious disease journal* 2010; **29**(11): 1035-9.
37. Bank TW. World Development Indicators.
38. J. S. GDL Area Database. Nijmegen, The Netherlands: Institute for Management Research, Radboud Universit, 2016.
39. Sur D, Chatterjee S, Riewpaiboon A, Manna B, Kanungo S, Bhattacharya SK. Treatment cost for typhoid fever at two hospitals in Kolkata, India. *J Health Popul Nutr* 2009; **27**(6): 725-32.
40. Riewpaiboon A, Piatti M, Ley B, et al. Cost of Illness Due to Typhoid Fever in Pemba, Zanzibar, East Africa. *Journal of Health Population and Nutrition* 2014; **32**(3): 377-85.
41. Shahunja KM, Leung DT, Ahmed T, et al. Factors Associated with Non-typhoidal Salmonella Bacteremia versus Typhoidal Salmonella Bacteremia in Patients Presenting for Care in an Urban Diarrheal Disease Hospital in Bangladesh. *PLoS neglected tropical diseases* 2015; **9**(9).
42. Drummond M, Sculpher M, Torrance G, O'Brien B, Stoddart G. Methods for the economic evaluation of health care programmes. 3rd ed. New York: Oxford University Press; 2005.
43. Feasey NA, Gaskell K, Wong V, et al. Rapid Emergence of Multidrug Resistant, H58-Lineage Salmonella Typhi in Blantyre, Malawi. *PLoS neglected tropical diseases* 2015; **9**(4).
44. Stenberg K, Lauer JA, Gkountouras G, Fitzpatrick C, Stanciole A. Econometric estimation of WHO-CHOICE country-specific costs for inpatient and outpatient health service delivery. *Cost Eff Resour Alloc* 2018; **16**: 11.
45. Poulos C, Riewpaiboon A, Stewart JF, et al. Cost of illness due to typhoid fever in five Asian countries. *Tropical Medicine & International Health* 2011; **16**(3): 314-23.
46. Chatterjee S, Levin C, Laxminarayan R. Unit Cost of Medical Services at Different Hospitals in India. *PloS one* 2013; **8**(7).
47. Vuong DA, Flessa S, Marschall P, Ha ST, Luong KN, Busse R. Determining the impacts of hospital cost-sharing on the uninsured near-poor households in Vietnam. *Int J Equity Health* 2014; **13**.
48. Ruhago GM, Ngalesoni FN, Robberstad B, Norheim OF. Cost-effectiveness of live oral attenuated human rotavirus vaccine in Tanzania. *Cost Effect Resour A* 2015; **13**.
49. Ayieko P, Akumu AO, Griffiths UK, English M. The economic burden of inpatient paediatric care in Kenya: household and provider costs for treatment of pneumonia, malaria and meningitis. *Cost Eff Resour Alloc* 2009; **7**: 3.

50. WHO. http://www.who.int/medicines/news/2017/Bharat-Biotech-TypbarTCV-WHO-PQ-Press-Release-Global-Final.pdf (accessed June 1st 2018).
51. Gavi. https://http://www.gavi.org/support/sustainability/transition-process/.
52. Portnoy A, Ozawa S, Grewal S, et al. Costs of vaccine programs across 94 low- and middle-income countries. *Vaccine* 2015; **33**: A99-A108.
53. Madsen LB, Ustrup M, Hansen KS, Nyasulu PS, Bygbjerg IC, Konradsen F. Estimating the costs of implementing the rotavirus vaccine in the national immunisation programme: the case of Malawi. *Tropical Medicine & International Health* 2014; **19**(2): 177-85.
54. Hutton G, Tediosi F. The costs of introducing a malaria vaccine through the expanded program on immunization in Tanzania. *American Journal of Tropical Medicine and Hygiene* 2006; **75**(2): 119-30.
55. Ngabo F, Levin A, Wang SA, et al. A cost comparison of introducing and delivering pneumococcal, rotavirus and human papillomavirus vaccines in Rwanda. *Vaccine* 2015; **33**(51): 7357-63.
56. Atherly DE, Lewis KDC, Tate J, Parashar UD, Rheingans RD. Projected health and economic impact of rotavirus vaccination in GAVI-eligible countries: 2011-2030. *Vaccine* 2012; **30**: A7-A14.
57. Gandhi G, Lydon P. Updating the evidence base on the operational costs of supplementary immunization activities for current and future accelerated disease control, elimination and eradication efforts. *BMC public health* 2014; **14**.
58. Kaucley L, Levy P. Cost-effectiveness analysis of routine immunization and supplementary immunization activity for measles in a health district of Benin. *Cost Effect Resour A* 2015; **13**.
59. Salomon JA, Vos T, Hogan DR, et al. Common values in assessing health outcomes from disease and injury: disability weights measurement study for the Global Burden of Disease Study 2010. *Lancet* 2012; **380**(9859): 2129-43.
60. Salomon JA, Haagsma JA, Davis A, et al. Disability weights for the Global Burden of Disease 2013 study. *Lancet Global Health* 2015; **3**(11): E712-E23.
61. Lo NC, Gupta R, Stanaway JD, et al. Comparison of Strategies and Thresholds for Vi Conjugate Vaccines against Typhoid Fever: A Cost-Effectiveness Modeling Study. *American Journal of Tropical Medicine and Hygiene* 2017; **97**(5): 617-.
62. Briggs AH, Goeree R, Blackhouse G, O'Brien BJ. Probabilistic analysis of cost-effectiveness models: Choosing between treatment strategies for gastroesophageal reflux disease. *Med Decis Making* 2002; **22**(4): 290-308.
63. Claxton K. The irrelevance of inference: a decision-making approach to the stochastic evaluation of health care technologies. *J Health Econ* 1999; **18**(3): 341-64.
64. Thokala P, Ochalek J, Leech AA, Tong T. Cost-Effectiveness Thresholds: the Past, the Present and the Future. *Pharmacoeconomics* 2018; **36**(5): 509-22.
65. Briggs A, Claxton K, Sculpher M. Decision Modelling for Health Economic Evaluation. New York: Oxford University Press; 2006.
66. Strong M, Oakley JE. An Efficient Method for Computing Single-Parameter Partial Expected Value of Perfect Information. *Medical Decision Making* 2013; **33**(6): 755-66.
67. Bertram MY, Lauer JA, De Joncheere K, et al. Cost-effectiveness thresholds: pros and cons. *B World Health Organ* 2016; **94**(12): 925-30.
68. Leech AA, Kim DD, Cohen JT, Neumann PJ. Use and Misuse of Cost-Effectiveness Analysis Thresholds in Low- and Middle-Income Countries: Trends in Cost-per-DALY Studies. *Value Health* 2018; **21**(7): 759-61.

69. Crump JA, Luby SP, Mintz ED. The global burden of typhoid fever. *B World Health Organ* 2004; **82**(5): 346-53.
70. Husereau D, Drummond M, Petrou S, et al. Consolidated Health Economic Evaluation Reporting Standards (CHEERS)--explanation and elaboration: a report of the ISPOR Health Economic Evaluation Publication Guidelines Good Reporting Practices Task Force. *Value Health* 2013; **16**(2): 231-50.
